# Intratumoral Fusogenic Outer Membrane Vesicles Enhance Antitumor Immunity Through Tumor MHC‐I Upregulation and Tumor Bacterization

**DOI:** 10.1002/advs.78279

**Published:** 2026-10-15

**Authors:** Xing Liu, Jiaxin Zhou, Guihong Lu, Chenjing Zhao, Weixiu Wang, Jichuan Chen, Fengqi Zhang, Fan Zhang, Hai‐Yan Xie, Weidong Nie

**Affiliations:** ^1^ Advanced Technology Research Institute (Jinan), School of Life Science Beijing Institute of Technology Beijing People's Republic of China; ^2^ State Key Laboratory of Natural and Biomimetic Drugs School of Pharmaceutical Sciences Chemical Biology Center Peking University Beijing People's Republic of China; ^3^ Department of Pathology Longgang Central Hospital of Shenzhen Shenzhen People's Republic of China; ^4^ Institute of Pediatrics Shenzhen Children's Hospital Affiliated Shenzhen Children's Hospital of Shantou University Medical College Shenzhen People's Republic of China

**Keywords:** antigen presentation, bacterial outer membrane vesicles, immunotherapy, major histocompatibility complex, tumor microenvironment

## Abstract

Cold tumors evade APC and T‐cell surveillance through low immunogenicity, defective antigen presentation, and immunosuppressive tumor microenvironments. We uncovered a non‐canonical function of bacterial outer membrane vesicles (OMVs), whereby OMVs activated the TLR4–NF‐κB–NLRC5 axis to induce tumor MHC I upregulation, thereby restoring tumor visibility to T cells. To further eliminate tumor invisibility to APCs, we reported a bacteria‐inspired strategy that drives APCs to perceive tumor cells as bacteria. By fusing OMVs with fusogenic liposomes, we constructed fusogenic OMVs (FOs) that anchor bacterial membrane components onto tumor surfaces, thereby functionally “bacterizing” tumor cells. This modification markedly increased APC recruitment and effectively reprogrammed APC perception from silent tumor clearance to active microbial‐like immune surveillance. Consequently, APCs exhibited enhanced phagocytic uptake of bacterized tumors, enabling efficient cross‐presentation of the tumor‐antigen repertoires to T cells. Concurrently, OMVs upregulated MHC I expression on tumor cells, which strengthened effector T cell recognition and cytotoxicity toward tumors. Furthermore, by loading 3‐bromopyruvate into the vesicles, we endowed the system with metabolic reprogramming capability, characterized by glycolysis suppression and lactate depletion. Together, the synergistic immune–metabolic modulation resulted in durable suppression of tumor progression following intratumoral administration, offering a bacterization‐inspired nanoplatform to overcome key immunological barriers in immune‐cold tumors.

## Graphical abstract

Tumor bacterization by fusogenic outer membrane vesicles enhances microbial‐like recognition of tumor cells while activating the TLR4–NF‐κB–NLRC5 axis to upregulate MHC class I expression. By simultaneously strengthening APC sensing and T‐cell recognition, this strategy breaks tumor immune invisibility and promotes more effective antitumor immune surveillance.

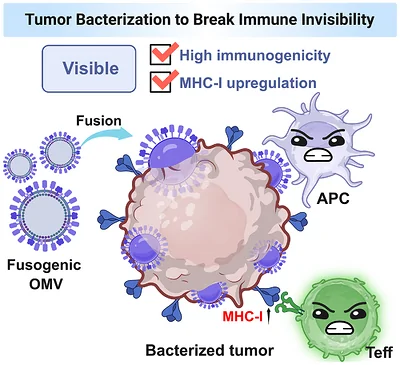

## 1 Introduction

Dual recognition of tumors by APCs and T cells represents a central hub for initiating effective antitumor immunity [1, 2]. As sentinel cells of immune surveillance, dendritic cells (DCs) capture antigens released from apoptotic tumor cells and cross‐present them to T cells, while activated effector T cells (Teffs) recognize and eliminate tumors [3, 4]. However, solid tumors, especially cold tumors, often evade host immune surveillance by establishing numerous barriers that impairDC and T cell recognition [5, 6]. Specifically, DC sensing of dying tumor cells and damage‐associated molecular patterns is impaired by ubiquitous efferocytosis mediated by tumor‐associated macrophages (TAMs) [7]. Concurrently, epigenetic silencing of tumor‐associated antigens (TAAs) and downregulation of MHC I molecules allow tumor cells to evade Teff recognition [8, 9, 10]. More critically, immunosuppressive cell populations, restricted nutrient availability, and accumulation of metabolic waste in the tumor microenvironment (TME), collectively impair the function and activity of tumor‐infiltrating DCs and Teffs [11, 12, 13]. Therefore, the immune resistance of cold tumors reflects the coordinated failure of antigen capture, tumor recognition, and effector function.

Intratumoral immunization and in situ cancer vaccination have emerged as promising strategies to overcome these barriers by converting tumors into endogenous antigen reservoirs while simultaneously providing local immune stimulation [14, 15, 16, 17]. By directly exploiting tumor‐resident antigens, these approaches can enhance APC activation, promote T‐cell priming, and, in some cases, induce systemic antitumor immunity beyond the injected lesion. However, most current strategies primarily emphasize antigen release or local adjuvant stimulation, and fewer are able to simultaneously enhance APC sensing of tumor cells, restore tumor visibility to Teffs, and relieve the suppressive TME. Growing evidence highlights the close interplay between commensal bacteria and antitumor immunity [18, 19]. A key bridge in this interaction is provided by bacterial outer membrane vesicles (OMVs), which naturally carry pathogen‐associated molecular patterns (PAMPs), such as lipopolysaccharides and outer membrane proteins [20, 21, 22, 23]. OMVs can enter the circulation and participate in antitumor immune responses by engaging Toll‐like receptors on APCs [24]. Beyond innate activation, OMVs are capable of remodeling the immunosuppressive TME, thereby facilitating APC‐ and Teff‐mediated antitumor immunity [25, 26, 27]. Owing to their intrinsic immunostimulatory properties that surpass artificial nanocarriers, OMVs have been widely explored as vaccine adjuvants and drug delivery vehicles [28, 29, 30, 31]. However, the systemic immunoregulatory mechanisms through which OMVs overcome the barriers of cold tumor immunity remain insufficiently understood. This gap highlights the need to re‐examine their biological functions and therapeutic potential from a new perspective.

Herein, we identified a previously unrecognized function of OMVs in upregulating tumor MHC I expression and elucidated the underlying mechanism. On this basis, we hybridized OMVs with fusogenic liposomes (Lip) to construct fusogenic OMVs (FOs), designed to “bacterize” tumor cells and thereby disrupt their immune invisibility (Scheme 1A). Upon intratumoral administration, FOs anchored OMVs onto tumor cell membranes via the fusogenic capability of liposomes, which substantially boosted tumor immunogenicity (Scheme 1B). This facilitated efficient APC recruitment and enhanced uptake of bacterized tumor cells, which presented the tumor antigen repertoire together with OMV‐derived adjuvanticity. Following migration to draining lymph nodes, OMV‐activated APCs cross‐presented tumor antigens to T cells, resulting in robust Teff activation. Meanwhile, OMVs upregulated tumor MHC I expression by activating the TLR4–NF‐κB–NLRC5 signaling axis, which in turn strengthened Teff recognition and cytotoxic responses against tumor cells. To strengthen the robustness and durability of therapeutic responses, we further loaded FOs with 3‐bromopyruvate (3BP@FOs). During FO–tumor fusion, 3BP was delivered directly into the tumor cytoplasm, where it bound to its target, hexokinase‐2 (HK‐II), to block glycolysis and reduce lactate accumulation in the TME. The 3BP acted synergistically with OMVs to reprogram the TME, thereby establishing durable APC‐ and Teff‐mediated adaptive immune responses against tumors. As a result, 3BP@FOs efficiently and safely suppressed tumor growth, metastasis, and recurrence, representing a promising immunometabolic nanoplatform to overcome the immunological barriers of cold tumors.

**SCHEME 1:**
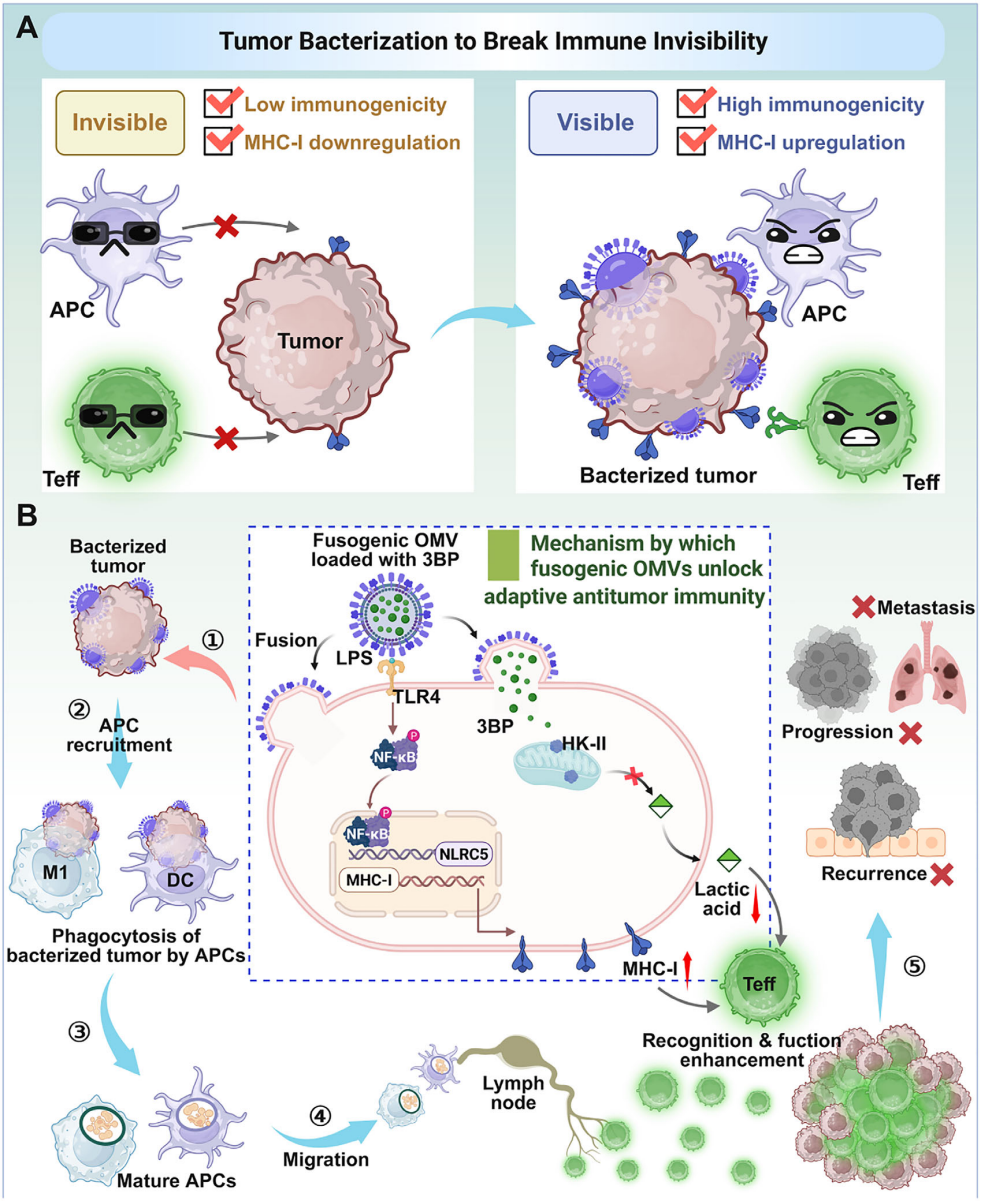
Fusogenic OMVs Break Immune Invisibility toward Tumors. (A) Fusogenic OMVs enhance tumor recognition by APCs and Teffs through tumor bacterization and MHC I upregulation. (B) Mechanism by which fusogenic OMVs unlock adaptive antitumor immunity. Created in BioRender. Zhang, F. (2026) https://BioRender.com/bso7j9c.

## 2 Results

### 2.1 OMVs Upregulate MHC I Expression on Tumor Cell Surface

Downregulation or loss of MHC class I (MHC I) molecules on the tumor cell surface is a common mechanism by which tumors evade recognition by cytotoxic Teff [32]. During co‐incubation of Escherichia coli BL21‐derived outer membrane vesicles (OMVs) with tumor cells, we unexpectedly observed that OMVs increased MHC I expression on B16 melanoma cells. At an OMV concentration of 1440 ng/mL, the mRNA levels of H‐2K and H‐2D were 2‐fold higher than those in the PBS control group (Figure 1A). Consistently, the proportion of MHC I^+^ tumor cells in the 1440 ng/mL OMV group increased by 62.73% compared to PBS, accompanied by a 2.89‐fold elevation in the mean MHC I expression level (Figure 1B,C; Figures S1 and S2A). In addition to melanoma, the OMV‐mediated upregulation of MHC I expression was also observed in MCF‐7 (human breast cancer) and A549 (human lung cancer) cells (Figure S2B,C). Consequently, both the presentation efficiency (percentage of OVA‐presenting cells) and the presentation level (mean presentation intensity) of the OVA model antigen were markedly enhanced on the B16‐OVA cells (Figure S3).

**FIGURE 1:**
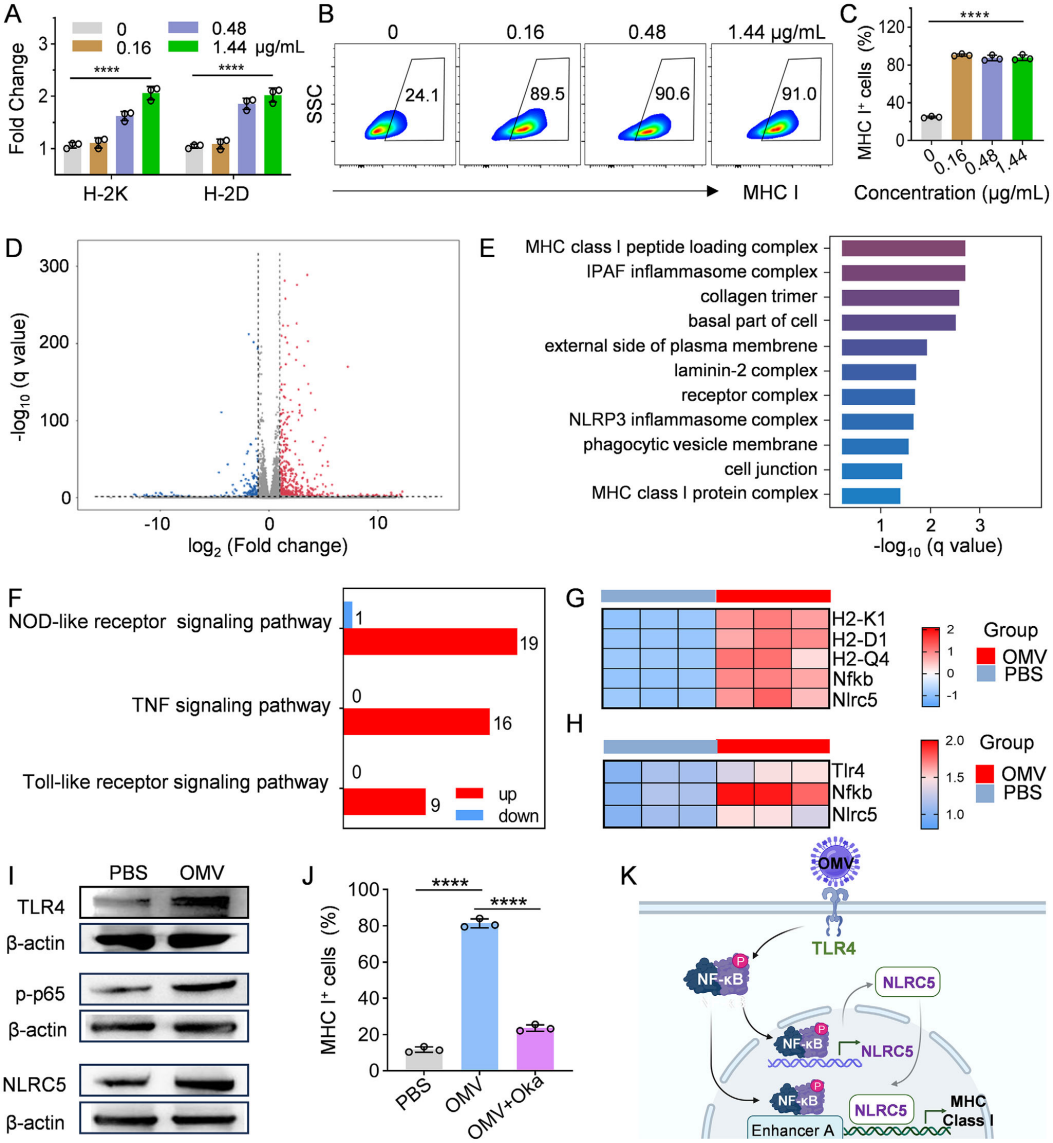
OMVs upregulate tumor MHC I expression via the TLR4–NF‐κB–NLRC5 axis. (A) Relative mRNA expression of H‐2K and H‐2D in B16 cells after 24 h treatment with different OMV concentrations. (B) Representative plots for flow cytometry analysis of MHC‐I⁺ B16 tumor cell. (C) Proportions of MHC I^+^ B16 cells after indicated treatments. (D) Volcano plot of differentially expressed genes in OMV versus control groups (red: significantly upregulated ≥2‐fold; blue: significantly downregulated ≥2‐fold). (E) GO enrichment of upregulated genes. (F) KEGG pathway enrichment of genes associated with innate and adaptive immune responses. (G‐H) Heat maps of MHC I–related genes detected by RNA‐seq (G) and qPCR (H). (I) Western blot analysis of TLR4 (≈100 kDa), p‐p65 (≈70 kDa), NLRC5 (≈225 kDa), and β‐actin (≈48 kDa) in tumor cells. (J) Proportions of MHC I^+^ tumor cells after indicated treatments. (K) Proposed mechanism of OMV‐induced MHC I upregulation through the TLR–NF‐κB–NLRC5 pathway. Created in BioRender. Zhang, F. (2026) https://BioRender.com/97sw6s6. Data are mean ± SD (n = 3). Statistical significance was determined using Student's t‐test or One‐way ANOVA. ****p < 0.0001.

To elucidate the potential mechanism, RNA transcriptome sequencing was performed on tumor cells before and after OMV treatment. Compared with PBS‐treated cells, OMV treatment significantly upregulated 504 genes, including MHC I–related genes such as H‐2K1, H‐2D1, and H2‐Q4 (Figure 1D, G). Gene Ontology (GO) analysis confirmed the enrichment  of MHC I peptide loading complex and MHC I protein complex terms (Figure 1E). Kyoto Encyclopedia of Genes and Genomes (KEGG) pathway enrichment analysis revealed significant enrichment of pathways associated with the activation of innate and adaptive immune responses, including the NOD‐like receptor (NLR) signaling pathway, Toll‐like receptor (TLR) signaling pathway, and TNF signaling pathway (Figure 1F). Among them, NLRs, a subset of cytosolic pattern recognition receptors (PRRs), initiate innate immune responses by detecting a wide range of pathogens and intracellular danger signals [33]. Within this family, NLR family CARD domain containing 5 (NLRC5) is a transcriptional activator specific to MHC I genes, whose expression can be induced by activation of the TLR signaling pathway [34]. Moreover, TLR signaling generally leads to NF‐κB activation, which promotes MHC I transcription initiation by binding to enhancer A within the MHC I promoter [35]. Our transcriptomic analysis showed that OMV treatment markedly increased Nlrc5 and Nfkb gene expression in tumor cells (Figure 1G). Given that OMVs are enriched in the TLR4 ligand, lipopolysaccharide (LPS), we hypothesized that OMVs enhance tumor cell MHC I expression by activating the TLR–NF‐κB–NLRC5 axis.

To verify this, we assessed the expression of key molecules before and after OMV treatment by qPCR and Western Blot analysis. Consistent with RNA‐seq data, OMV exposure significantly upregulated Nlrc5 and Nfkb at both mRNA and protein levels (Figure 1G–I; Figure S4). Additionally, as reported previously, OMV treatment also increased TLR4 expression on tumor cells, likely due to LPS‐mediated activation (Figure 1H,I) [36, 37, 38]. Subsequently, okanin was introduced into the OMV–B16 co‐culture system to inhibit TLR4 and NF‐κB activity [39, 40], which markedly attenuated the OMV‐induced upregulation of surface MHC I expression (Figure 1J; Figure S5). Collectively, these results demonstrate that OMVs enhance tumor cell surface MHC I expression by activating the TLR4–NF‐κB–NLRC5 signaling pathway (Figure 1K).

### 2.2 Construction of Fusogenic OMVs to Bacterize Tumor Cells

The upregulation of tumor MHC I by OMVs suggests their potential to enhance Teff cell‐mediated tumor recognition and cytolytic activity. However, within the TME, DCs are generally reduced in number, functionality, and antigen‐presenting capacity, thereby limiting the supply of Teff cells [2, 4]. Thus, restoring the abundance of functional DCs and their tumor antigen‐presenting ability is critical for sustaining sufficient Teffs within the TME. Pathogens, in contrast, can rapidly mobilize APCs and promote efficient cross‐presentation [41, 42], which inspired us to explore strategies for bacterizing tumor cells. To this end, fusogenic OMVs (FOs) were engineered by hybridizing OMVs with membrane‐fusogenic liposomes, with the aim of incorporating OMVs onto tumor cell membranes to generate bacteria‐mimicking tumor cells (Figure 2A,B). To evaluate the ability of FOs to display OMVs on tumor cell surfaces, liposomes were hybridized with varying amounts of OMVs to prepare FOs, which were then co‐incubated with B16 tumor cells. When the mass ratio of OMVs to liposomes was 4:1, FOs exhibited fusion efficiency with B16 cells comparable to that observed at a 1:1 ratio (Figure S6). Under these conditions, abundant LPS signals were detected on the tumor cell surface. As the proportion of liposomes decreased, the fusion efficiency with tumor cells was correspondingly reduced. In contrast, pristine OMVs and the physical mixture of OMVs and liposomes (1:1) showed only weak adsorption onto the tumor cell surface (Figure S6). At the 4:1 ratio, the two components also exhibited strong colocalization of fluorescence signals, indicating excellent hybridization between OMVs and liposomes (Figure 2C). With the aid of fusogenic lipids, OMVs were effectively presented on B16 tumor cell membranes, thereby generating tumor cells with bacterial‐like features (Figure 2D; Figures S7 and S8). The fusion stability evaluation demonstrated that OMVs could persist on the tumor cell surface, during which the proportion of LPS^+^ tumor cells decreased from ≈85.9% to ≈64.1% within 12 h (Figure S8). Considering that APCs can be recruited to sites of bacterial invasion within as little as 1–3 h, and rapidly recognize and phagocytose bacteria within minutes to 1 h [43, 44, 45], the sustained bacterization of tumors by FOs provides a sufficient time window for enhanced APC recruitment and tumor recognition.

**FIGURE 2:**
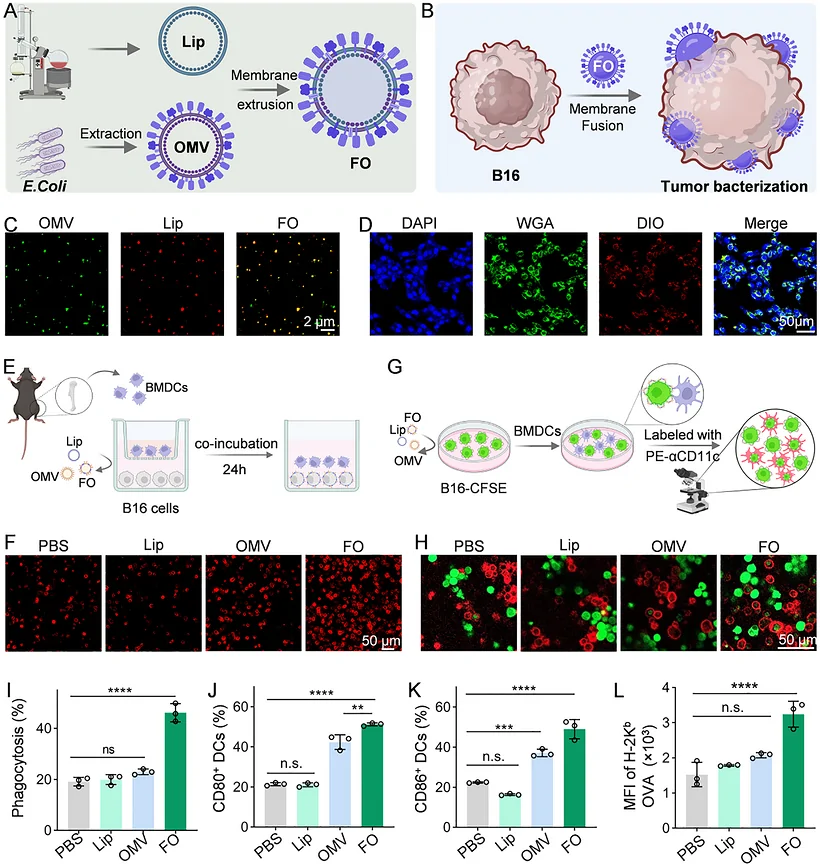
Construction of fusogenic OMVs (FOs) enables tumor bacterization. (A‐B) Schematic illustration of FO preparation (A) and tumor bacterization (B). (C) Fluorescence imaging of FOs (green: LPS antibody–labeled OMVs; red: DiI‐labeled liposomes). (D) Confocal imaging of bacterized tumor cells (green: WGA‐labeled membrane; red: DiO‐labeled FOs; blue: DAPI). (E) Transwell assay schematic for DC recruitment. (F) Fluorescence images of DC recruitment by tumor cells with indicated treatments (red: CD11c antibody–labeled DCs). (G) Schematic illustration of the phagocytosis of tumor cells by DCs. (H, I) Fluorescence images and flow cytometry analysis of the phagocytosis of tumor cells by DCs (red: CD11c antibody DCs; green: CFSE‐labeled tumor cells). (J–K) Proportions of CD80^+^ and CD86^+^ DCs (gated on CD11c^+^ cells) after different treatments. (L) Mean fluorescence intensity of H‐2K^b^ OVA on DCs after different treatments. Data are mean ± SD (n = 3). Statistical significance was determined using Student's t‐test or One‐way ANOVA. ****p < 0.0001, ***p < 0.001, **p < 0.01; n.s., not significant. Figures (A, B, E, and G) were created in BioRender. Zhang, F. (2026) https://BioRender.com/rlrk6bw.

### 2.3 Bacteria‐Mimicking Tumor Cells Mobilize and Promote DC Antigen Presentation

To investigate whether bacterized tumor cells could recruit DCs, carboxyfluorescein succinimidyl ester (CFSE)‐labeled B16 cells were seeded in the lower chamber of a Transwell system and incubated with various nanovesicles for 20 min. After removing free vesicles, bone marrow‐derived DCs (BMDCs) were added to the upper chamber and cultured for 24 h, followed by CD11c staining (Figure 2E). Minimal BMDC recruitment was observed in the PBS and liposome groups (Figure 2F). Pristine OMV pretreatment led to a moderate increase in BMDC recruitment, likely due to the upregulation of tumor‐derived chemokines such as CCL5 (Figure S9) [46]. In contrast, FO‐treated bacteria‐mimicking tumor cells recruited the greatest number of BMDCs. To evaluate BMDC phagocytosis, CFSE‐labeled B16 cells pretreated with different nanovesicles were co‐cultured with BMDCs after removal of free vesicles (Figure 2G). As shown in Figure 2H, strong colocalization of green tumor fluorescence within red BMDCs was observed in the FO group, whereas tumor cells in the PBS, liposome, and OMV groups remained largely separated from BMDCs, with negligible internalization. Quantitative analysis revealed that while OMV‐treated tumor cells induced mild BMDC recruitment, BMDC uptake remained comparable to the PBS group (Figure 2I; Figure S10). By contrast, bacterized tumor cells exhibited more than a 1.9‐fold increase in BMDC uptake efficiency relative to pristine tumor cells.

OMVs are enriched in pathogen‐associated molecular patterns (PAMPs) that engage DC pattern recognition receptors, thereby promoting maturation and serving as adjuvants to enhance tumor antigen presentation [22]. To assess whether bacterized tumor cells could improve these processes, DCs were co‐cultured with B16‐OVA cells pretreated with different nanovesicles. OMV‐treated tumor cells modestly increased the frequency of CD80^+^/CD86^+^ DCs but exhibited OVA antigen presentation efficiency comparable to the PBS group, consistent with the limited internalization of tumor cells and intratumoral OMVs by DCs (Figure 2J–L; Figures S11, S12). In contrast, bacterized tumor cells markedly enhanced both DC maturation and OVA antigen presentation, attributable to greater tumor internalization combined with OMV‐mediated DC activation.

### 2.4 Bacterized Tumor Cells Elicit Robust Adaptive Antitumor Immunity

Consistent with previous findings, DCs rarely phagocytosed intact tumor cells and instead mainly processed free tumor antigens [4]. However, intact tumor cells contain the full antigenic repertoire of tumor cells, offering both greater quantity and diversity compared to isolated antigens [47, 48]. Thus, internalization of bacterized tumor cells by DCs may facilitate more potent Teff activation and expansion against diverse tumor antigens. To confirm this, DCs matured by tumor cells treated with different nanovesicles were co‐cultured with splenic T cells (Figure 3A). The proportion of activated CD8^+^CD69^+^ T cells in the FO group increased by 2.8‐fold compared with PBS, whereas the OMV group achieved only a modest increase due to limited tumor and OMV uptake (Figure 3B, Figures S13 and S14).

**FIGURE 3:**
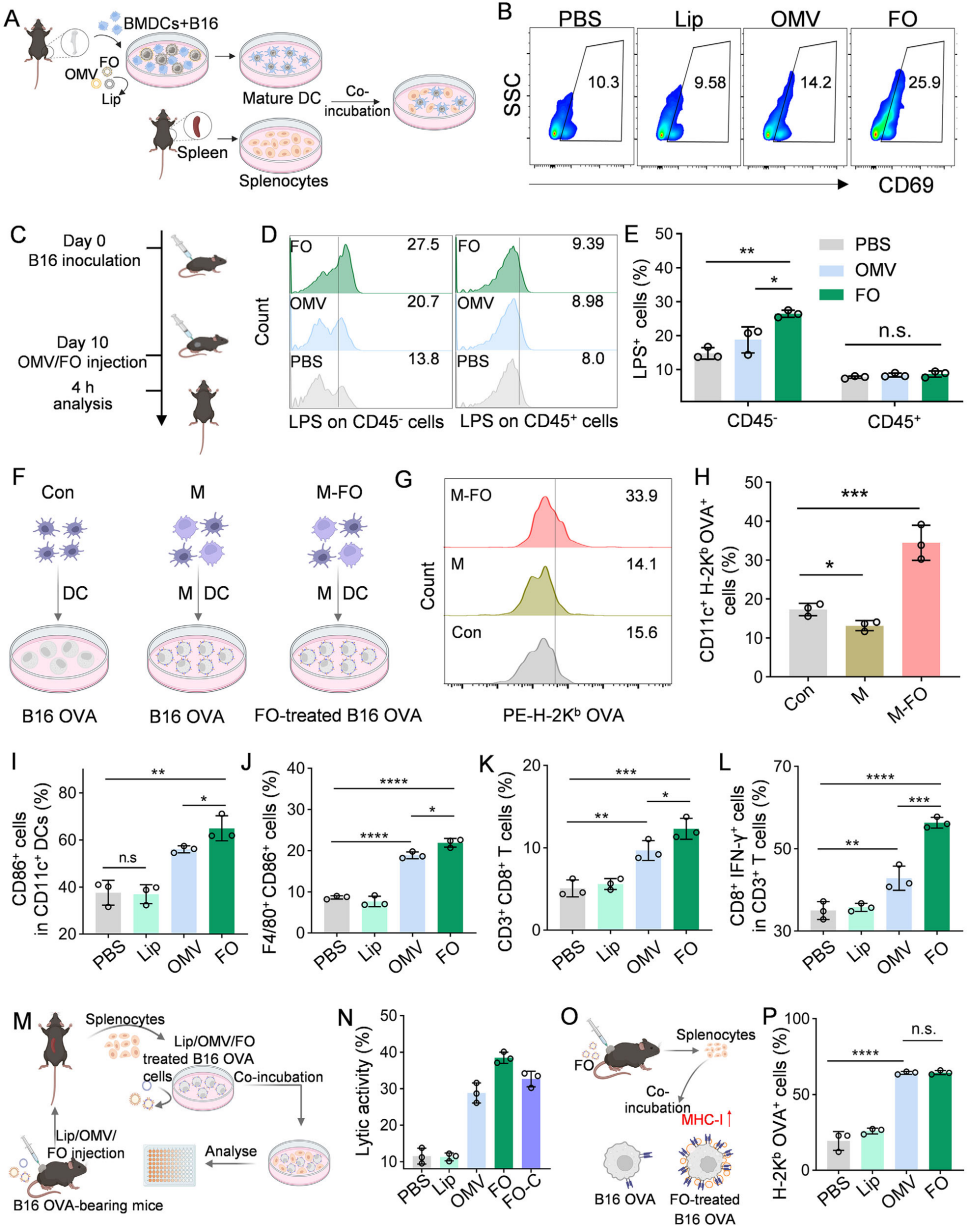
Bacterized tumor cells activate robust adaptive antitumor immunity. (A) Schematic illustration of in vitro T‐cell activation. (B) Representative flow cytometry plots of CD69^+^ T cells (gated on CD3^+^CD8^+^ cells). (C) Experimental design for evaluating tumor bacterization. (D, E) Expression of OMV markers (LPS) on CD45^−^ and CD45^+^ cells. (F) Schematic illustration of the evaluation of M2 macrophage effects on DC antigen presentation. (G) Representative histograms for flow cytometry analysis of DC antigen presentation after M2 macrophage co‐culture. (H) Presentation of OVA antigens on CD11c^+^ DCs. (I, J) Quantification of CD86^+^ DCs (I; gated on CD45^+^CD11c^+^ cells) and CD86^+^ macrophages (J; gated on CD45^+^F4/80^+^ cells) in tumors. (K, L) Frequencies of CD3^+^CD8^+^ T cells and CD8^+^IFN‐γ^+^ T cells (gated on CD3^+^ cells) in lymph nodes. (M) Schematic illustration of T‐cell killing against tumors. (N) Specific lysis of B16‐OVA cells by T cells from spleens after indicated treatments. (O) Schematic illustration of MHC I on tumor cells after indicated treatments. (P) H‐2K^b^ OVA expression on tumor cells after indicated treatments. Data are mean ± SD (n = 3). Statistical significance was determined using Student's t‐test or One‐way ANOVA. ****p < 0.0001, ***p < 0.001, **p < 0.01, *p < 0.05; n.s., not significant. Figures (A, C, F, M and O) were created in BioRender. Zhang, F. (2026) https://BioRender.com/awr2bvm.

Next, the ability of FO to activate adaptive antitumor immune responses in vivo was evaluated (Figure 3C). Consistent with the in vitro findings, FO treatment successfully endowed tumor cells with bacteria‐like features, as evidenced by the appearance of LPS on the membrane of CD45^−^ tumor cells (Figure 3D,E; Figure S15). Quantitative analysis revealed that the proportion of LPS^+^ tumor cells (CD45^−^) in the FO group was 1.8‐fold higher than in the PBS group, whereas the proportion of LPS^+^ cells within the CD45^+^ immune population remained largely unchanged. Upon phagocytosis of these bacteria‐mimicking tumor cells by APCs, robust APC maturation will be induced.

Macrophages, one of the major APC populations, can constitute over half of the tumor mass [49]. However, TAMs typically exhibit an immunosuppressive M2 phenotype, driving T cell exhaustion by cross‐presenting tumor antigens and even suppressing DC antigen uptake via efferocytosis [7, 50]. Our prior work demonstrated that reprogramming TAMs to the M1 phenotype restores antigen‐presenting function and enhances DC antigen uptake [51, 52]. As expected, co‐culture of FO‐treated B16‐OVA cells with M2 macrophages reduced expression of the M2 marker CD206 while increasing expression of the M1 marker CD86 (Figure S16). These reprogrammed macrophages displayed robust phagocytic activity and enhanced antigen‐presenting capacity toward bacteria‐mimicking tumor cells (Figures S17, S18). Furthermore, given that M2 macrophages can clear tumor antigens through efferocytosis, their reprogramming to the M1 phenotype was expected to facilitate DC‐mediated antigen presentation. To verify this, pristine M2 macrophages or FO‐reprogrammed M2 macrophages were separately introduced into a B16‐OVA/BMDC co‐culture system, after which OVA antigen presentation by BMDC was evaluated (Figure 3F). As shown in Figure 3G,H, the presence of M2 macrophages reduced DC antigen presentation efficiency by 24% (Figure S19). In contrast, replacing M2 macrophages with their M1‐reprogrammed counterparts restored DC antigen presentation efficiency from 17.3% to 34.5%.

Consistent with the in vitro findings, FO treatment induced higher levels of mature APCs in TME (Figure 3I,J; Figures S20, S21). These mature APCs subsequently migrated to the draining lymph nodes for initiating robust adaptive immune responses (Figures S22, S23). As a result, FO treatment yielded the greatest proportions of both CD4^+^ and CD8^+^ T cells in the draining lymph nodes, which increased by 2.1‐ and 2.4‐fold, respectively, relative to the PBS group (Figure 3K; Figures S24, S25). In particular, the proportion of cytotoxic T lymphocytes (CD8^+^ IFNγ^+^) was elevated by 1.6‐fold (Figure 3L; Figure S25). We next evaluated the lytic activity of these activated T cells against tumor cells. Splenic T cells were isolated from B16‐OVA tumor‐bearing mice after treatment with the different nanovesicles and co‐cultured for 24 h with B16‐OVA cells subjected to treatments consistent with the in vivo administration hydrodynamic (Figure 3M). Tumor cell lysis was assessed by LDH release. As expected, splenic T cells from the FO group exhibited the strongest tumoricidal activity among all groups (Figure 3N). Importantly, OMVs upregulated tumor surface MHC I expression and enhanced antigen presentation, which were retained in liposome‐hybridized FOs (Figure 3P; Figures S26, S27). Consequently, splenic T cells from FO‐treated mice displayed significantly greater cytotoxicity against FO‐bacterized tumor cells (FO group) than against pristine tumor cells (FO‐C group), highlighting the critical role of MHC I upregulation in enhancing T cell‐mediated tumor recognition and killing (Figure 3N,O).

### 2.5 Metabolic Inhibitor–Loaded FOs Reprogram the TME for Durable Antitumor Immunity

The pervasive immunosuppressive signals within the TME drive functional exhaustion of both APCs and Teffs [53]. Significantly, membrane fusion not only enables lipid and protein intermixing between two membranes but also mediates the exchange of their encapsulated cargo [54]. This feature provides an opportunity to enhance FO‐mediated antitumor immunity by incorporating therapeutic agents that sustain APC and Teff viability. Given that lactate accumulation in the TME suppresses APC and Teff survival and function while promoting regulatory T cell (Treg) proliferation, 3BP was selected as a hexokinase II (HK‐II) inhibitor to block tumor glycolysis and thereby alleviate lactate‐driven immunosuppression [55, 56]. UV–vis spectroscopy confirmed successful 3‐BP encapsulation within FOs, with a loading efficiency of 20.9% (Figure S28). The resulting 3BP@FOs exhibited ahydrodynamic diameter of ∼134.7 nm and maintained excellent colloidal stability for at least 5 days (Figure S29). The protein composition of OMVs was preserved during fabrication (Figure S30). In addition to presenting OMVs on the tumor cell surface, fusogenic 3BP@FOs efficiently delivered 3‐BP into the tumor cytoplasm, where it directly interacted with HK‐II to inhibit glycolytic activity (Figure 4A,B). Consequently, 3BP@FOs exhibited markedly greater tumor uptake of 3‐BP (CD45^−^FITC^+^) and stronger inhibition of HK‐II compared with free 3‐BP, which suffers from poor membrane permeability (Figure 4C; Figures S31, S32). Notably, due to strong phagocytic capacity exhibited by APCs, drug‐derived fluorescence was also detected in DCs (CD45^+^CD11c^+^) and macrophages (CD45^+^CD11b^+^) (Figures S33, S34). Since LPS was absent from the surface of these immune cells (Figure 3E), we infer that 3BP@FOs were predominantly internalized by APCs via phagocytosis. Although this potentially reduces the likelihood of 3BP@FOs fusing with tumors, successful fusion was nevertheless observed in Figure 3E upon intratumoral injection. Consequently, intratumoral lactate levels were reduced by 1.5‐fold and 1.3‐fold in the 3BP@FO group relative to PBS and free 3‐BP, respectively, thereby facilitating immune cell recruitment, infiltration, and activation (Figure 4D) [57].

**FIGURE 4:**
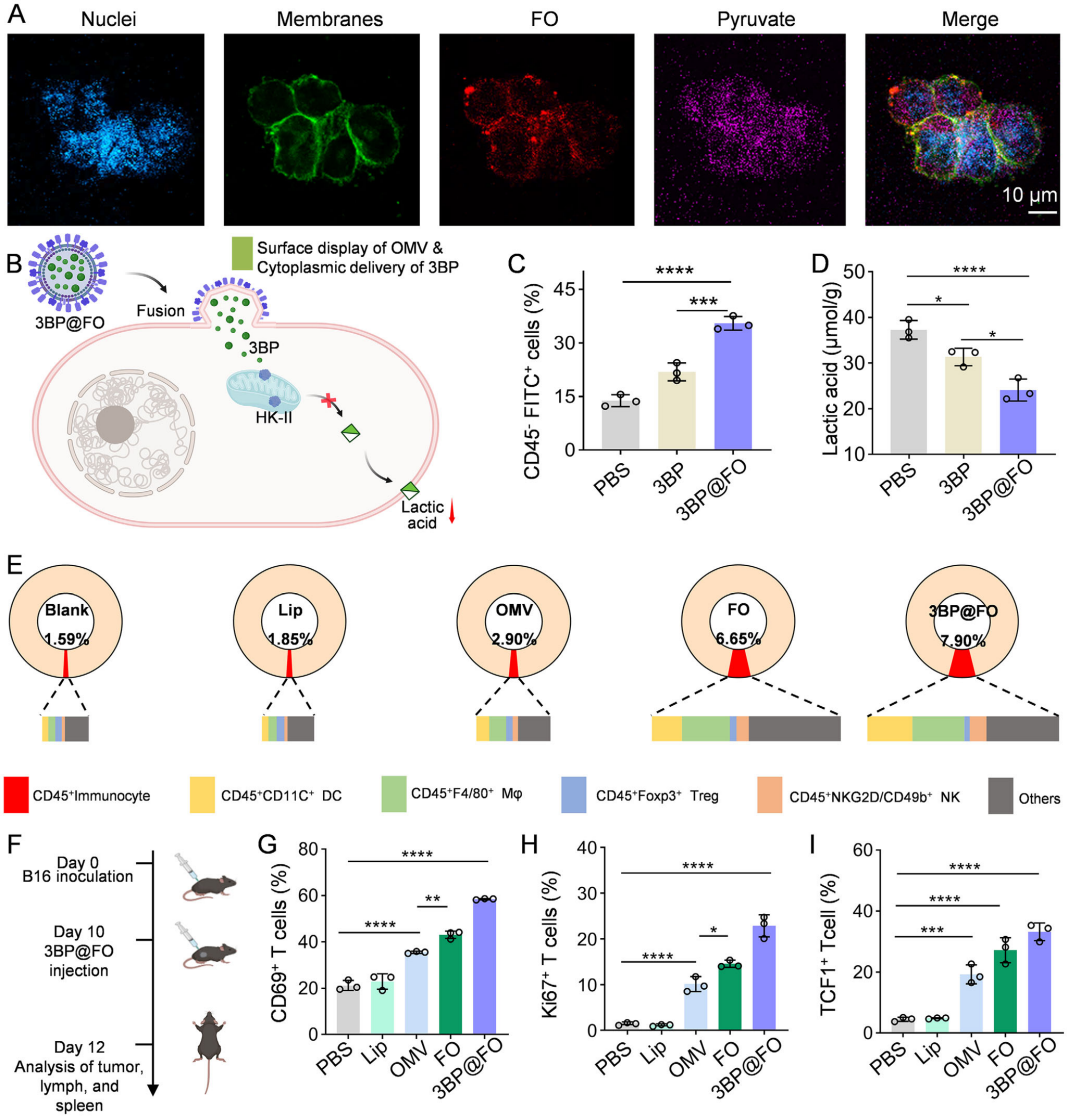
3BP@FOs remodel the immunosuppressive TME to promote sustained antitumor immunity. (A) Confocal imaging of B16 cells incubated with 3BP@FOs (green: DiI‐labeled membranes; red: DiD‐labeled FOs; purple: FITC‐pyruvate). (B) Schematic illustration of membrane fusion and cytoplasmic delivery. (C) Tumor uptake of FITC‐pyruvate by flow cytometry. (D) Lactate levels in tumor tissues after treatments. (E) Flow cytometry analysis of tumor‐infiltrating immune cells. (F) Experimental design for evaluating immune infiltration. (G–I) Proportions of CD8^+^CD69^+^, CD8^+^Ki67^+^, and CD8^+^TCF1^+^ T cells. Data are mean ± SD (n = 3). Statistical significance was determined using Student's t‐test or One‐way ANOVA. ****p < 0.0001, ***p < 0.001, **p < 0.01, *p < 0.05. Figure (B and F) were created in BioRender. Zhang, F. (2026) https://BioRender.com/ktgaeoo.

To further evaluate the impact of tumor bacterization combined with metabolic reprogramming on TME remodeling, we characterized the intratumoral immune cell landscape across the different treatment groups (Figure 4F). Significantly, all OMV‐containing treatments markedly increased the overall intratumoral immune cell (CD45^+^) content compared with the untreated group (Figure 4E; Figure S35). As a synergistic outcome of OMV integration and glycolysis inhibition, the numbers of DCs (CD45^+^CD11c^+^) and macrophages (CD45^+^F4/80^+^) recruited to the tumor site in the 3BP@FO group increased by 1.9‐fold and 3.4‐fold, respectively, relative to PBS, and exceeded those observed in both the FO and OMV groups (Figure 4E; Figures S36, S37). As a result, 3BP@FO treatment led to the highest level of CD8^+^ T cell infiltration within tumor tissues, and the CD8^+^/CD4^+^ T cell ratio, a key prognostic indicator of immunotherapeutic efficacy, was significantly increased in the 3BP@FO group compared with the PBS group (01.3 vs. 0.5) (Figure S38).

In addition to APCs and T cells, NK cell abundance in the 3BP@FO group increased by 3.1‐fold compared with PBS, largely attributable to OMV‐mediated recruitment (Figure 4E; Figure S39) [58]. Importantly, the abundance of Treg cells, potent suppressors of T cell activity, was markedly reduced across OMV‐containing groups. In particular, Treg cell numbers in the 3BP@FO group were decreased by 6.2‐fold and 3.2‐fold compared with PBS and OMV groups, respectively, further demonstrating the synergistic effects of OMV‐driven bacterization and 3BP‐mediated lactate depletion (Figure 4E; Figure S40). As a result, the activation level of intratumoral CD8^+^ T cells, measured by CD69 expression, rose from 21.1% in PBS to 58.4% in the 3BP@FO group (Figure 4G; Figure S41). Moreover, comprehensive TME remodeling by 3BP@FO sustained long‐term CD8^+^ T cell functionality, as evidenced by elevated Ki‐67 (proliferative capacity) and TCF‐1 (stem‐like potential) expression (Figure 4H,I; Figures S42, S43). Collectively, 3BP@FOs not only enhanced tumor immunogenicity but also remodeled the TME into an immune‐permissive state, thereby promoting sustained recruitment and activation of effector immune cells.

### 2.6 3BP@FO Suppresses Tumor Progression and Enhances Checkpoint Blockade Responsiveness

Given the improvement in long‐term T cell functionality through comprehensive TME remodeling by 3BP@FO, we next evaluated its therapeutic efficacy against immune‐cold melanoma (Figure 5A). The results showed that Lip alone did not significantly inhibit tumor growth (Figure 5B,C). OMV and FO treatments achieved tumor inhibition rates of 59.5% and 72.3%, respectively, confirming that OMVs can elicit potent antitumor immunity in vivo (Figure 5D). In addition, OMVs upregulated tumor MHC I expression, thereby enhancing immune cell recognition and cytotoxicity (TUNEL assay) toward tumors (Figure 5F; Figure S44). FO, by strengthening both APC‐ and T cell–mediated recognition of tumor cells, exhibited superior tumor suppression compared with OMV alone (Figure 5B–D). Notably, 3BP@OMV provided additional tumor suppression relative to OMV, yet this effect remained weaker than that achieved by 3BP@FO. Correspondingly, 3BP@FO, which integrates the immune‐priming effects of FO with glycolysis blockade by 3‐BP, achieved optimal maintenance of CD8^+^ T cell activity and the most pronounced TME remodeling. Consequently, 3BP@FO induced marked tumor cell apoptosis (TUNEL assay) and inhibition of tumor proliferation (Ki‐67 staining) (Figure 5E). Despite a tumor control rate of 81.62%, complete tumor regression was not achieved, likely due to the inherently immune‐cold nature of melanoma. The median survival in the 3BP@FO group was 38 days, representing a 12‐day extension compared with the PBS group (Figure 5F). These results suggested that further enhancement of antitumor T‐cell responses might be required, which prompted us to explore the potential of 3BP@FO to synergize with immune checkpoint blockade therapy. To this end, anti–PD‐1(αP)‐based combination regimens were compared in the B16 model. Among all treatments evaluated, 3BP@FO+αP achieved the strongest tumor suppression and the most pronounced survival benefit, with a tumor suppression efficiency of 91.09% and 50% of mice surviving beyond day 50, whereas all mice in the PBS group died within 27 days (Figure 5G–I). Notably, as expected for the immune‐cold B16 model, αP alone conferred only a limited survival benefit over PBS, extending median survival by 1.5 days. These results demonstrate the strong potential of 3BP@FO to simultaneously enhance APC‐ and T cell–mediated tumor recognition, leading to enhanced responsiveness to checkpoint blockade therapy. During treatment, no significant body weight loss was observed in any group, indicating good tolerability in animals (Figure S45). Moreover, in vivo fluorescence imaging was performed after intratumoral administration of DiR‐labeled OMV and 3BP@FO. The fluorescence signals remained largely confined to the tumor region for up to 72 h and showed no evident accumulation in major organs (Figure S46), suggesting favorable tumor retention and minimal off‐target exposure. In addition, H&E staining of major organs, serum inflammatory cytokine levels, and biochemical markers of tissue injury revealed no abnormalities of concern, further confirming the safety of the 3BP@FO‐based therapy (Figures S47, S48).

**FIGURE 5:**
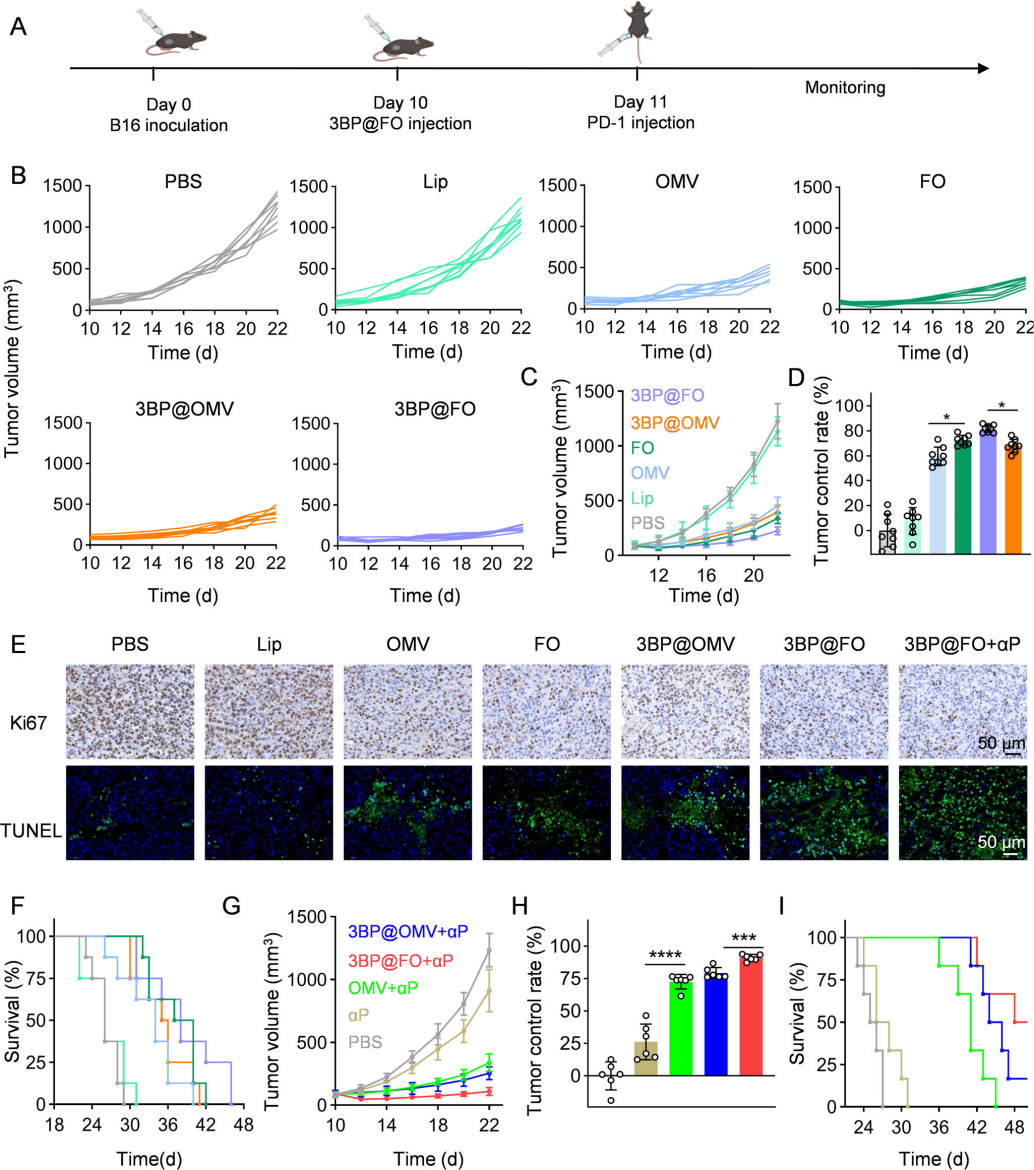
3BP@FOs suppress melanoma progression in vivo. (A) Schematic illustration of B16 tumor model and therapy. Figure was created in BioRender. Zhang, F. (2026) https://BioRender.com/ybu38rl. (B, C) Tumor growth curves. (D) Tumor inhibition rates. (E) Ki67 and TUNEL staining of tumor tissues at the endpoint. (F) Survival percentages of mice. (G–I) Tumor growth curves (G), tumor inhibition rates (H), and survival percentages (I) of mice receiving the indicated αP‐based combination treatments. Data in (B–F) are mean ± SD (n = 8). Data in (G–I) are mean ± SD (n = 6). Statistical significance was determined using Student's t‐test or One‐way ANOVA. ****p < 0.0001, ***p < 0.001, *p < 0.05.

Given the limited monotherapy efficacy of 3BP@FO in the highly immune‐cold B16 model, we next evaluated its efficacy in CT26 colon carcinoma, a relatively more immunotherapy‐responsive tumor model (Figure S49). Compared with the B16 model, 3BP@FO showed a more pronounced therapeutic effect in CT26. Specifically, all mice in the PBS group died within 32 days. OMV and FO monotherapy extended median survival by 6.5 and 10 days, respectively. By contrast, 3BP@FO achieved the strongest tumor suppression and the most pronounced survival benefit in CT26, with a tumor suppression efficiency of 88.71% and 33.3% of mice surviving beyond day 60. These findings support the broader applicability of the 3BP@FO‐based intratumoral immunometabolic strategy.

### 2.7 3BP@FO Induces Short‐ and Long‐Term Immune Memory to Prevent Melanoma Metastasis and Recurrence

Melanoma is highly susceptible to metastasis and recurrence following surgical resection [59]. To assess whether 3BP@FO could induce protective antitumor memory, splenic memory T cell differentiation was analyzed 48 h after administration. Notably, 3BP@FO treatment increased the proportion of effector memory T cells (CD44^+^CD62L^−^) by 5.7‐fold compared with PBS group (Figure 6A,B; Figure S50). Given that lung metastases are among the most common distant lesions in melanoma, a B16 pulmonary metastasis model was established (Figure 6C). Significantly, 3BP@FO treatment displayed a marked increase in CD8^+^ T cells in the lung, along with reduced pulmonary tumor colonization, with the number of metastatic foci being only one‐fifth of that in the PBS group (Figure 6D–F; Figure S51). These results demonstrate that 3BP@FO limits melanoma metastasis through the induction of immunological memory. We next evaluated the ability of 3BP@FO to prevent tumor recurrence in a postoperative rechallenge model, wherein B16 cells were re‐inoculated into the surgical resection site (Figure 6G). As expected, tumors in the PBS group rapidly recurred within 22 days post‐rechallenge, whereas 3BP@FO treatment conferred strong resistance to recurrence (Figure 6H). Consistently, Ki67 staining of recurrent tumor tissues revealed that the 3BP@FO group exhibited the weakest proliferative signal among all treatment groups (Figure S52). At the experimental endpoint, splenic T cell analysis revealed that OMV, FO, 3BP@OMV, and 3BP@FO treatments increased the proportion of CD8^+^ T cells by 21.66%, 32.36%, 27.3%, and 36.38%, respectively, compared with PBS (Figure 6I; Figure S53). Notably, 3BP@FO induced the highest levels of effector memory T cells, which were 3.76‐, 2.21‐, 1.75‐, and 1.83‐fold higher than those in the PBS, OMV, FO, and 3BP@OMV groups, respectively (Figure 6J,K; Figure S54). Collectively, these findings indicate that 3BP@FO can effectively elicit both short‐ and long‐term immune memory responses, thereby enhancing the therapeutic durability of immunotherapy in immunologically cold tumors while preventing melanoma metastasis and recurrence.

**FIGURE 6:**
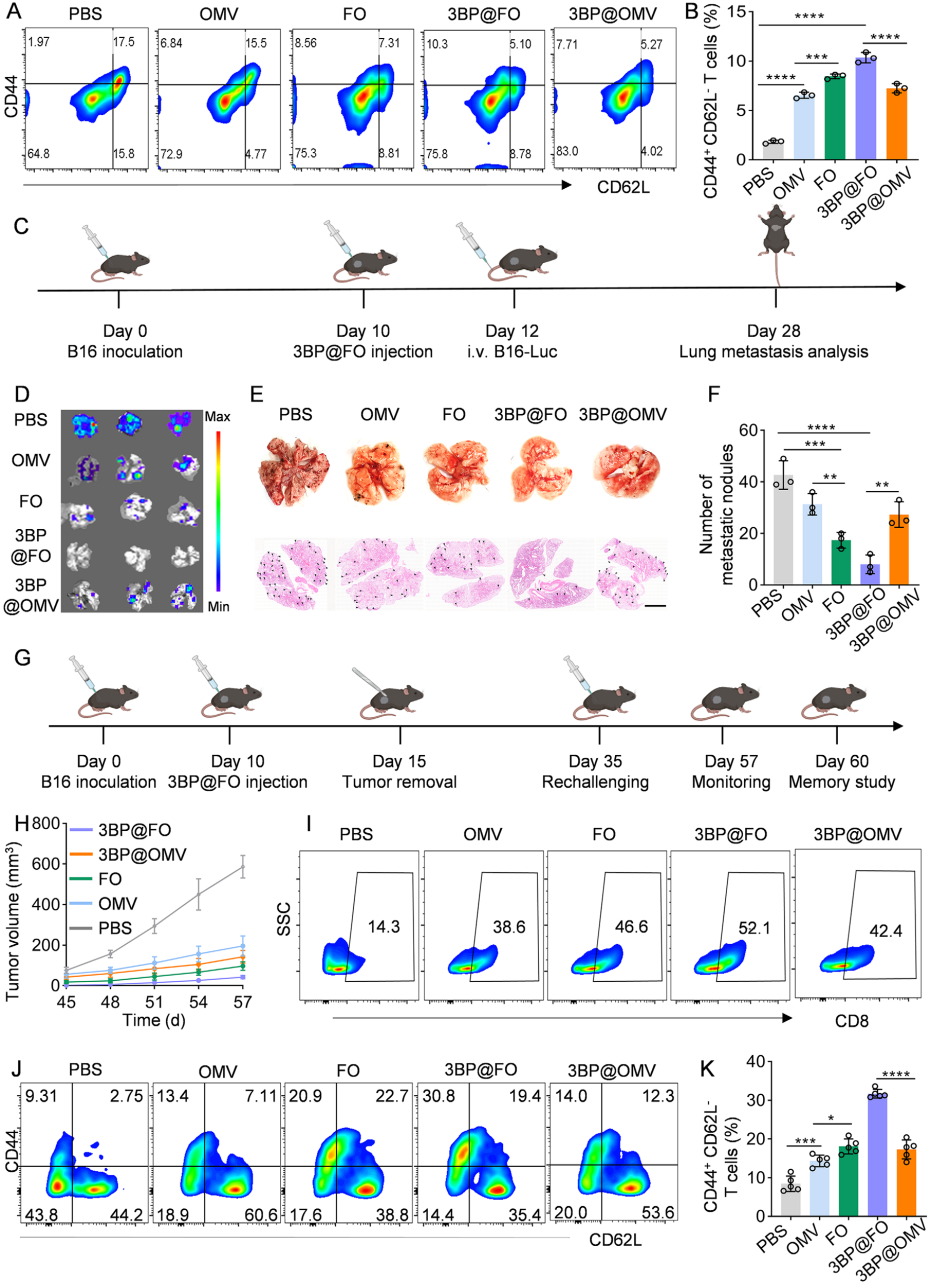
3BP@FOs prevent melanoma metastasis and recurrence. (A, B) Frequencies of CD44^+^CD62L^−^ effector memory T cells (gated on CD3^+^CD8^+^ cells) in draining lymph nodes. Data are mean ± SD (n = 3). (C) Schematic illustration of B16 lung metastasis model. (D, E) Representative lung bioluminescence images, photographs and H&E‐staining with metastatic nodules (arrows). Scale bar: 3 mm. (F) Quantification of lung nodules. Data are mean ± SD (n = 3). (G) Schematic illustration of B16 tumor rechallenge model. (H) Growth curves of recurrent tumors. (I–K) Flow cytometry analysis of CD8^+^ T cells (I) and effector memory T cells (J, K) in spleens at the endpoint. Data in H are mean ± SD (n = 6). Statistical significance was determined using Student's t‐test or One‐way ANOVA. ****p < 0.0001, ***p < 0.001, **p < 0.01, *p < 0.05. Figure (C and G) were created in BioRender. Zhang, F. (2026) https://BioRender.com/yp4y0zj.

## 3 Discussion

Many solid tumors are characterized by low immunogenicity and inefficient antigen presentation, which severely limits the ability of APCs and Teffs to recognize tumor cells. Considerable progress has been made in improving APC‐ or T cell–mediated tumor recognition [60, 61, 62, 63]. However, approaches limited to a single recognition pathway fail to fully activate the cancer–immune cycle required for effective tumor elimination [64, 65]. In this study, we constructed fusogenic OMVs to bacterize tumor cells while upregulating tumor surface MHC I expression. The bacterization reprogrammed intrinsic tumor cell features, enabling them more recognizable as “non‐self” by APCs. At the same time, OMVs activated the TLR4/NF‐κB/NLRC5 signaling axis, which induced MHC I upregulation and promoted Teff recognition of tumors. By concurrently enhancing both APC‐ and T cell–mediated tumor recognition, fusogenic OMVs effectively disrupted the immune‐evasive state of cold tumors.

Upon loading with 3BP, the resulting 3BP@FOs not only retained these immunogenic effects but also remodeled the intratumoral immune composition, thereby reducing immunosuppressive factors in the TME. The combined effect reinforced the long‐term activity of APCs and Teffs, which in turn promoted durable immune memory formation. Consequently, 3BP@FOs achieved potent suppression of tumor growth, metastasis, and recurrence, with favorable safety and survival benefits. Importantly, through simultaneous intervention at the cell membrane and cellular metabolism, 3BP@FOs create a multidimensional regulatory system that strengthens the robustness and durability of therapeutic responses. Moreover, the platform can be readily integrated with immune checkpoint inhibitors, adoptive T cell transfer, or cancer vaccines, providing a versatile strategy to enhance therapeutic responses in cold tumors.

Intratumoral administration of this nanoplatform further enhanced local bioavailability while minimizing systemic exposure, thereby reducing the risk of immune‐related adverse events [66]. Our study revealed that FOs preferentially fused with CD45^−^ tumor cells rather than with CD45^+^ immune cells (Figure 3E; Figures S33–S56). This selectivity was likely attributable to differences in phagocytic capacity and membrane charge [67]. Nevertheless, the sequestration of some FOs by APCs was unavoidable, which compromised their ability to fuse with tumor cells. Importantly, this non‐tumor‐cell‐specific uptake also raises the possibility that 3BP may directly affect APC metabolism and function [68]. Although our data support inhibition of tumor‐cell glycolysis and reduced lactate accumulation as an important mechanism of 3BP@FO treatment, direct metabolic effects of 3BP on APCs cannot be excluded. The extent of these effects is likely to depend on the overall intracellular exposure of APCs to 3BP. Cell type‐specific metabolic profiling and targeted perturbation will be needed to distinguish tumor‐cell‐mediated effects from direct immune‐cell effects and to better define the therapeutic window of 3BP@FO [69]. Additionally, the possible participation of other intratumoral cell types in the fusion process remains largely unexplored. Future efforts to decorate FO surfaces with tumor‐targeting ligands may further improve selective fusion and enhance localized immunoactivation. Another limitation is that direct assessment of antigen cross‐presentation in the present study was primarily based on the B16‐OVA model and detection of H‐2K^b^–SIINFEKL complexes. Thus, although whole tumor cells provide access to a broad repertoire of endogenous antigens, our current data directly demonstrate enhanced presentation of the model antigen OVA rather than simultaneous presentation of multiple endogenous tumor antigens. Immunopeptidomic analysis and antigen‐specific T‐cell profiling will be required to further define the breadth of endogenous antigen presentation induced by FOs. In addition, systematic analysis of microenvironmental regulation, multi‐model validation, and rational integration with other immunotherapeutic modalities will be essential to unlock the translational potential of this platform in the treatment of immunologically cold tumors.

## 4 Methods

### 4.1 Materials and Reagents

RPMI‐1640 medium, penicillin–streptomycin, and 0.25% trypsin–EDTA were purchased from Macgene (China). Fetal bovine serum (FBS) was obtained from PAN‐Seratech (Germany). L‐lactate assay kit, micro‐BCA protein assay kit, SDS–PAGE loading buffer, and pre‐stained protein marker were purchased from Solarbio (China). DMPC (CAS No. 18194‐24‐6) and DOTAP (CAS No. 144189‐73‐1) were obtained from Aladdin (China). DSPE‐ mPEG2000 (CAS No. ‌147867‐65‐0) was supplied by Ponsure (China). Chloroform (CAS No. 865‐49‐6) was obtained from Mreda (China). Okanin (CAS No. 484‐76‐4) and 3‐bromopyruvate (CAS No. 1113‐59‐3) were purchased from MedChemExpress (USA). Hoechst 33342 was purchased from Life Technologies (USA). iFluor555 wheat germ agglutinin (WGA) conjugate was obtained from AAT Bioquest (USA). DIO (Cat. No. SR3831) and DID (Cat. No. SR3032) were purchased from Harveybio. The CFSE Cell Proliferation Kit was obtained from Invitrogen (USA). Recombinant mouse IL‐4 and GM‐CSF were purchased from Novoprotein (China). AF647‐conjugated anti‐Escherichia coli LPS antibody (Cat. No. bs‐8000R‐AF647), anti‐NFκB p65 (p‐S536) antibody (Cat. No. bs‐0982R), and anti‐Hexokinase 2 antibody (Cat. No. bs‐9455R) were obtained from Bioss (China). Anti‐NLRC5 antibody (Cat. No. 72379) was purchased from Cell Signaling Technology (USA). Anti‐actin antibody (Cat. No. 20536‐1‐AP), HRP‐conjugated goat anti‐rabbit IgG antibody (Cat. No. SA00001‐2), and HRP‐conjugated goat anti‐mouse IgG antibody (Cat. No. SA00001‐1) were obtained from Proteintech. The staining buffer for flow cytometry was obtained from eBioscience (USA). The True‐Nuclear Transcription Factor Buffer Set and antibodies for flow cytometry were purchased from BioLegend (USA), including FITC anti‐mouse CD45 (Cat. No. 157213), APC‐Cy7 anti‐mouse CD11c (Cat. No. 117324), APC anti‐mouse CD86 (Cat. No. 159216), APC anti‐mouse CD3 (Cat. No. 100236), PE anti‐mouse CD8a (Cat. No. 162304), BV605 anti‐mouse CD69 (Cat. No. 104529), FITC anti‐mouse Ki67 (Cat. No. 151212), Pacific Blue anti‐mouse CD49b (Cat. No. 108918), PE anti‐mouse NKG2D (Cat. No. 115606), APC‐Cy7 anti‐mouse CD25 (Cat. No. 101918), PE anti‐mouse Foxp3 (Cat. No. 118904), PE anti‐mouse IFN‐γ (Cat. No. 505808), FITC anti‐mouse CD44 (Cat. No. 156008), Pacific Blue anti‐mouse CD62L (Cat. No. 161207), FITC anti‐mouse H‐2K^b^/H‐2D^b^ (Cat. No. 114606), Pacific Blue anti‐mouse CD4 (Cat. No. 100428), and PE anti‐mouse H‐2K^b^ OVA (Cat. No. 141603).

### 4.2 Bacterial Strains, Cell Lines, and Animals

Escherichia coli BL21 strain, murine melanoma B16, and B16‐OVA cell lines were kindly provided by Peking University Health Science Center. All cell lines were cultured in RPMI‐1640 medium supplemented with 10% fetal bovine serum (FBS) and 1% penicillin–streptomycin, and maintained at 37°C in a humidified incubator with 5% CO_2_.

Female C57BL/6N mice were purchased from Peking University Health Science Center. Mice were maintained under specific pathogen‐free conditions with controlled temperature (20–22°C) and humidity (30%–70%), and provided with sufficient food and water. All animal experiments were approved by the Animal Ethics Committee of Beijing Institute of Technology (approval no. 2019‐0011‐176) and were conducted in accordance with all relevant ethical guidelines and regulations.

### 4.3 Culture of E. coli BL21 and Extraction of OMVs

E. coli BL21 was cultured in LB medium containing 5 g/L yeast extract, 10 g/L tryptone, and 10 g/L NaCl. Briefly, 20 µL of frozen bacterial stock was inoculated into 3 mL of LB medium and incubated at 37°C with shaking at 220 rpm for 12 h. The activated culture was then transferred into 1 L of fresh LB medium and further grown under the same conditions for 6 h until the optical density at 600 nm (OD_600_) reached 1.0.

OMVs were isolated from the E. coli BL21 culture. Briefly, the bacterial culture was centrifuged at 8,000 × g for 10 min using a Sorvall ST16R centrifuge (Thermo Fisher Scientific, USA) to remove bacterial cells. The supernatant was collected and filtered through a 0.45 µm acetate membrane (Corning, USA), followed by concentration using a 100 kDa molecular weight cut‐off centrifugal filter (Merck Millipore, USA). The concentrated supernatant was then passed through a second 0.45 µm membrane to further remove residual contaminants and bacteria. The filtrate was subsequently ultracentrifuged at 150,000 × g for 3 h at 4°C using a Beckman Coulter XPN‐100 ultracentrifuge (USA). The resulting OMV pellet was resuspended in PBS, stored at −80°C for further use, and quantified using a Micro BCA Protein Assay Kit. Under these culture and purification conditions, the OMV yield was approximately 1.0–1.33 mg per liter of bacterial culture.

### 4.4 Mechanistic Study of OMV‐Induced Upregulation of Tumor MHC Expression

B16, B16‐OVA, MCF‐7, and A549 Cells (1 × 10^5^ Cells per Well) Were Seeded Into 24‐well Plates and Cultured Overnight. Cells Were Then Treated With Different Concentrations of OMVs (0, 160, 480, or 1440 Ng/mL) for 24 h at 37°C. For Flow Cytometry Analysis, Cells Were Stained With FITC‐conjugated Anti‐H‐2K^b^/H‐2D^b^ and PE‐conjugated Anti‐H‐2K^b^ OVA Antibodies on Ice for 30 Min in the Dark, Washed Three Times With PBS to Remove Unbound Antibodies, and Analyzed Using a BD FACS Aria III Flow Cytometer (Bd Biosciences, Usa). To Further Investigate the Mechanism by Which OMVs Upregulate Tumor MHC Expression, B16 Cells (1 × 10^6^) Were Seeded Into Six‐well Plates and Cultured Overnight. Cells Were Treated With OMVs (0 or 1440 Ng/mL) for 24 h at 37°C. Total RNA Was Extracted Using TransZol Up Reagent, and Lysed Samples Were Incubated on Ice for 10 Min. Cell Lysates Were Transferred Into RNase‐free Tubes and Stored at −80°C for Subsequent Transcriptomic Analysis. For Transcriptomic Analysis, P Values Were Adjusted for Multiple Testing Using the Benjamini–Hochberg Procedure to Control the False Discovery Rate (FDR). Differentially Expressed Genes Were Identified Using a Q Value < 0.05 and |log2 Fold Change| ≥ 1. The Same Multiple‐testing Correction Was Applied to GO Enrichment Analysis, and GO Terms With Q < 0.05 Were Considered Significantly Enriched. For qPCR Analysis, 20 µL Chloroform Was Added to each Lysate, Followed by Vigorous Shaking and a 10 Min Incubation. Samples Were Centrifuged at 12,000 × G for 15 Min, and 500 µL of the Upper Aqueous Phase Was Collected and Mixed With 500 µL Isopropanol. After a 30 Min Incubation on Ice, Samples Were Centrifuged at 12,000 × g for 15 Min. The Supernatant Was Discarded, and the RNA Pellet Was Washed With 70% Ethanol, Gently Mixed, and Centrifuged Again at 12,000 × G for 5 Min. The Pellet Was Air‐dried and Dissolved in 20 µL DEPC‐treated Water. RNA Concentration Was Determined Using a NanoDrop Spectrophotometer. cDNA Was Synthesized Using the TransScript One‐Step gDNA Removal and cDNA Synthesis SuperMix Kit According to the Manufacturer's Instructions and Stored at −80°C. The Resulting cDNA Was Used for Subsequent qPCR Analysis Using the PerfectStart Green qPCR SuperMix Kit. For Western Blot Analysis, Protein Samples Extracted From the Lysed Cells Were Separated by SDS–polyacrylamide Gel Electrophoresis (SDS–PAGE) and Transferred Onto Nitrocellulose (Nc) Membranes (Millipore, Usa). The Membranes Were Blocked With 5% Bovine Serum Albumin (BSA) for 3 h at Room Temperature, Followed by Overnight Incubation at 4°C With the Primary Antibodies, Including Anti‐TLR4 (1:1000), Anti‐NLRC5 (1:1000), Anti‐NF‐κB (1:1000), and Anti‐β‐actin (1:5000). After Three Washes With PBST (PBS Containing 0.05% Tween‐20), the Membranes Were Incubated With Horseradish Peroxidase (HRP)‐conjugated Goat Anti‐rabbit IgG (1:10000) or Goat Anti‐mouse IgG (1:10000) Secondary Antibodies for 2 h at Room Temperature. After another Three PBST Washes, Protein Bands Were Visualized Using Enhanced Chemiluminescence (ECL) Reagents and Imaged on a Bio‐Rad Chemiluminescence System. For Functional Inhibition Studies, B16 Cells (1 × 10^6^ per Well) Were Seeded in Six‐well Plates and Cultured Overnight. Cells Were Then Treated With OMVs (0 or 1440 Ng/mL) for 24 h at 37°C, Followed by Incubation With Okanin (a TLR4/NF‐κB Inhibitor, 6 µM) for 4 h. The Treated Cells Were Stained With PE–Cy7–conjugated Anti–MHC I Antibody, Washed Three Times With PBS, and Analyzed by Flow Cytometry

### 4.5 Synthesis of Liposomes

DMPC, DOTAP, and DSPE–mPEG2000 Were Dissolved in Chloroform to Prepare 1 mg/mL Stock Solutions. Liposomes Were Prepared by Mixing the Three Lipids at a Molar Ratio of 76.2:3.8:20 (DMPC:DSPE–mPEG:DOTAP) With a Total Mass of 10 mg in a 100 mL Round‐bottom Flask. The Mixture Was Stirred Magnetically for 30 Min at 37°C, While Chloroform Was Evaporated Using a Rotary Evaporator (Shanghai Yarong Biochemical Instrument Co., China) at 70–80 Rpm. After Complete Solvent Removal, a Uniform Lipid Film Formed on the Flask Wall, Which Was Further Dried Overnight Under Vacuum to Eliminate Residual Chloroform. The Dried Lipid Film Was Rehydrated in PBS and Sequentially Extruded Through 800 Nm, 400 Nm, and 100 Nm Polycarbonate Membranes to Obtain Liposomes. The Liposomes Were Stored at −80°C Until Use. For Fluorescent Labeling, DiI or DiO Dye Was Added to the Lipid Mixture at a 1:500 Molar Ratio During Preparation, and all Steps Were Performed Under Light‐protected Conditions

### 4.6 Preparation of FO and Tumor Bacterization

OMVs and DiO‐labeled Liposomes Were Mixed at Mass Ratios of 0:1, 1:1, 4:1, or 8:1 (OMV:Lip), Ultrasonicated for 30 Min, and Incubated at 37°C for 1 h in the Dark. The Mixtures Were Repeatedly Extruded Through a Membrane Extruder With 100 Nm Polycarbonate Membranes to Obtain Fusogenic OMVs (FOs). For Tumor Cell Bacterization, B16 Cells (2 × 10^5^ per Well) Were Seeded Into Glass‐bottom 24‐well Plates and Cultured Overnight in RPMI‐1640 Medium Containing 10% FBS. Cells Were Then Treated With 50 µg of FOs With Different OMV‐to‐liposome Ratios at 37°C for 20 Min in the Dark. During this Process, the FOs Can Successfully Fused With the Tumor Cell Membrane, While Effectively Preventing the Internalization and Nonspecific Adsorption of Pristine OMVs by Tumor Cells. After Incubation, OMVs on the Tumor Surfaces and Nuclei Were Stained With AF647‐conjugated Anti‐E. coli LPS Antibodies and Hoechst 33342/DAPI, Respectively. After Three PBS Washes, the Cells Were Imaged by Confocal Laser Scanning Microscopy (CLSM; Nikon, Japan) to Assess Membrane Fusion Efficiency. For OMV Uptake Analysis, OMVs Were Labeled With DiO Dye and Incubated at 37°C for 30 Min. Unbound Dye Was Removed Using NapTM‐5 SephadexTM Columns. Labeled OMVs (10 µg/mL) Were Added to B16 Cells and Incubated for 20 Min at 37°C. Cells Were Washed Three Times With PBS, Stained With 2 µL WGA–iFluor555 for 20 Min in the Dark, Washed Again, and Imaged by CLSM to Visualize OMV Distribution Within Tumor Cells

### 4.7 Evaluation of OMV Retention on Tumor Cell Surfaces

B16 Cells (2 × 10^5^ per Well) Were Seeded Into Six‐well Plates and Cultured Overnight. Cells Were Incubated With FOs (OMV:Lip = 4:1) at 37°C for 20 Min to Facilitate Membrane Fusion. Excess FOs Were Removed, and the Cells Were Further Incubated for Various Durations (0, 4, 6, and 12 h). The OMVs Retained on the Tumor Cell Surface Were Labeled With AF647–conjugated Anti–E. coli LPS Antibody. After Three PBS Washes, the Cells Were Analyzed by Flow Cytometry

### 4.8 Recruitment of Dendritic Cells by Bacterized Tumors

Bone Marrow–derived Dendritic Cells (BMDCs) Were Obtained From the Femurs and Tibias of C57BL/6 Mice and Cultured for 7 Days in Medium Supplemented With GM‐CSF (20 Ng/mL) and IL‐4 (20 Ng/mL). For DC Recruitment Assays, B16 Cells (2 × 10^5^ per Well) Were Seeded Into the Lower Chambers of 24‐well Transwell Plates and Cultured Overnight. Cells Were Treated With PBS, OMVs, Lip, or FO at 37°C for 20 Min to Allow Membrane Fusion, Followed by Three PBS Washes to Remove Unbound Vesicles. BMDCs Were Then Added to the Upper Chambers at a 1:1 Ratio With B16 Cells and Cocultured for 24 h at 37°C. BMDCs in the Lower Chamber Were Subsequently Stained With APC‐conjugated Anti‐CD11c Antibody and Imaged by CLSM

### 4.9 In Vitro Phagocytosis of Tumor and Antigen Presentation by Antigen‐Presenting Cells

For the in Vitro Phagocytosis Assay, B16 Cells (5 × 10^4^ Cells per Well) Were Seeded Into 24‐well Plates and Cultured Overnight. The Cells Were Then Labeled With 5 µM CFSE Dye at Room Temperature for 10 Min and Washed Three Times With PBS. Subsequently, PBS, OMVs, Lip, or FO Vesicles Were Added to the CFSE‐labeled Cells and Incubated at 37°C for 20 Min to Facilitate Membrane Fusion. The Treated Tumor Cells Were Mixed With Bone Marrow‐derived Dendritic Cells (BMDCs) or M2‐type Macrophages at a 1:1 Ratio and co‐cultured for 24 h. The Mixed Cells Were Stained With PE‐CD11c and PerCP‐CD11b Antibodies to Label BMDCs and M2 Macrophages, Respectively. Flow Cytometry Was Then Performed to Analyze the CFSE Fluorescence Intensity Within the CD11c^+^ and CD11b^+^ Gated Populations. The Phagocytosis Efficiency of APCs Toward B16 Cells Was Further Evaluated Using a High‐content Imaging System (CellVue T2000, MegaRobo). To Further Assess Dendritic Cell Maturation, M2 Macrophage Repolarization, and the Antigen‐presenting Efficiency of these APCs, B16‐OVA Cells (2 × 10^5^ per Well) Were Seeded Into 24‐well Plates and Cultured Overnight. After Treatment With PBS, OMVs, Lip, or FO Vesicles at 37°C for 20 Min to Promote Membrane Fusion, Excess Vesicles Were Removed, and BMDCs or M2 Macrophages Were Added for co‐culture Over 48 h. BMDCs Were Stained With PerCP‐CD11c, FITC‐CD80, APC‐CD86, and PE‐H‐2K^b^ OVA Antibodies (1:100 Dilution) to Evaluate Maturation and Antigen Presentation. M2 Macrophages Were Stained With PerCP‐CD11b, Pacific Blue‐CD206, APC‐CD86, and PE‐H‐2K^b^ OVA Antibodies to Analyze Phenotype Repolarization and Antigen‐presenting Activity

### 4.10 Macrophage Phenotype Reprogramming Enhances DC‐Mediated Antigen Presentation

Mouse Peritoneal Macrophages (1 × 10^5^ Cells per Well) Were Seeded in 24‐well Plates and Treated With IL‐4 (100 Ng/mL) for 48 h to Induce M2 Polarization. The M2 Macrophages and BMDCs Were Then co‐cultured With either Untreated B16‐OVA Cells (M Group) or FO‐treated B16‐OVA Cells (M–FO Group) for 24 h. BMDCs Were Stained With APC‐CD11c and PE‐H‐2K^b^ OVA Antibodies, Followed by Flow Cytometric Analysis to Quantify OVA Antigen Presentation Across Groups

### 4.11 In Vitro T Cell Activation Assay

B16 Cells (5 × 10^4^ per Well) Were Seeded Into 24‐well Plates and Cultured Overnight. The Cells Were Treated With PBS, OMVs, Lip, or FO Vesicles at 37°C for 20 Min to Induce Membrane Fusion, and Then co‐cultured With BMDCs at a 1:1 Ratio for 48 h. Splenocytes Were Isolated From 8‐week‐old C57BL/6 Mice and co‐incubated With the Pretreated BMDCs at a 10:1 Ratio for 24 h. After Incubation, Cells Were Harvested and Stained With APC‐CD3, FITC‐CD8, and PE‐CD69 Antibodies for Flow Cytometric Analysis to Evaluate Activation Efficiency of T Cells

### 4.12 In Vivo Membrane Fusion of FO Vesicles With Tumor Cells

B16 Tumor Cells (5 × 10^5^ Cells/100 µL) Were Suspended in PBS and Subcutaneously Injected Into Female C57BL/6N Mice. When the Tumor Volume Reached Approximately 300 Mm^3^, the Mice Were Randomly Divided Into Three Groups. Each Group Received an Intratumoral Injection of 25 µL PBS, OMVs, or FO Vesicles (OMV Protein Content: 5 µg per Injection). Four Hours Post‐injection, the Mice Were Euthanized, and the Tumors Were Excised and Dissociated Into Single‐cell Suspensions. The Cells Were Stained With FITC‐CD45 and AF647‐labeled Anti‐E. coli LPS Antibodies and Analyzed by Flow Cytometry to Determine FO Fusion Efficiency With Different Cell Populations in Vivo

### 4.13 In Vivo Immune Activation Assay

B16 Tumor Cells (5 × 10^5^ Cells/100 µL) Were Subcutaneously Injected Into Female C57BL/6N Mice to Establish a Subcutaneous Tumor Model. When Tumors Reached Approximately 300 Mm^3^, the Mice Were Randomly Divided Into Four Groups and Intratumorally Injected With 25 µL PBS, OMVs, Lip, or FO Vesicles (OMV Protein Content: 5 µg per Injection). After 48 h, the Mice Were Euthanized, and Tumors, Spleens, and Tumor‐draining Lymph Nodes (TDLNs) Were Collected, Homogenized, and Analyzed. For Tumor Tissues, Dendritic Cells Were Stained With FITC‐CD45, APC‐Cy7‐CD11c, and APC‐CD86 Antibodies, While Macrophages Were Stained With FITC‐CD45, PE‐F4/80, and APC‐CD86 Antibodies. Tumor Cells Were Stained With FITC‐CD45 and PE‐Cy7‐MHC I Antibodies to Assess MHC I Expression. For Lymphoid Tissues, DCs Were Stained With APC‐Cy7‐CD11c and APC‐CD86 Antibodies; Macrophages With PE‐F4/80 and APC‐CD86 Antibodies; and T Cells With APC‐CD3, Pacific Blue‐CD4, APC‐Cy7‐CD8, and PE‐IFN‐γ Antibodies. After Three Washes With PBS, Cells Were Analyzed by Flow Cytometry. To Evaluate the Tumor‐killing Capacity of in Vivo–activated T Cells, Splenocytes From each Treatment Group Were co‐cultured With Their Corresponding B16 Tumor Cells for 24 h, and Cytotoxicity Was Assessed Using a Lactate Dehydrogenase (LDH) Assay. To Further Investigate the Impact of MHC I Upregulation on T‐cell‐mediated Lysis, T Cells From FO‐treated Mice Were co‐cultured With either Untreated B16 Cells (FO–C Group) or FO‐pretreated B16 Cells, and Tumor Cell Viability Was Determined Using the LDH Assay Kit

### 4.14 Preparation and Characterization of 3BP@FO

3BP and FO Were Mixed in PBS at Mass Ratios of 4:1, 2:1, 1:1, 1:2, and 1:4 to a Final Volume of 200 µL. The Mixtures Were Transferred Into Electroporation Cuvettes and Subjected to Electroporation Under the Following Parameters: 100 V, 200 Ω, and 100 µF. After Electroporation, the Samples Were Incubated at 37°C for 30 Min. Ultrafiltration (100 kDa Filter, 2500 G, 20 Min) Was Performed to Collect Retentate, Which Was Repeatedly Washed With PBS to Remove Unbound 3BP. The Ultraviolet Absorption Spectra of 3BP Were Measured to Establish a Standard Curve, and the Concentration of 3BP in the Filtrate Was Quantified Using a UV Spectrophotometer. The Loading Efficiency and Encapsulation Efficiency of 3BP@FO Were Calculated as Follows:

Loadingefficiency(%)=3BPload/(FOcontent+3BPload)×100

EncapsulationEfficiency(%)=3BPLoad/3BPInput×100

The hydrodynamic size and zeta potential were measured using dynamic light scattering (DLS, Malvern Instruments). Protein samples from 3BP@FO were separated by SDS‐PAGE. The gels were stained with Coomassie Brilliant Blue at 70 rpm for 8 h, destained, and imaged using an iBright imaging system (Thermo Fisher Scientific, USA).

### 4.15 Intratumoral Drug Distribution and Lactate Quantification

B16 tumor cells (5 × 10^5^ cells in 100 µL PBS) were subcutaneously inoculated into female C57BL/6N mice. When the tumor volume reached approximately 300 mm^3^, the mice were randomly divided into three groups and intratumorally injected with 25 µL of PBS, FITC–pyruvate, or FO‐loaded FITC–pyruvate. Four hours after injection, the mice were euthanized, and tumor tissues were collected and mechanically dissociated into single‐cell suspensions. For flow cytometry analysis, the cells were stained with APC‐Cy7–CD45, Pacific Blue–CD11b, APC–CD11c, PE–NKG2D, and PE–CD3 antibodies to identify total immune cells, macrophages, dendritic cells (DCs), natural killer (NK) cells, and T cells, respectively. For intratumoral lactate quantification, a separate cohort of B16 tumor‐bearing mice received intratumoral injections of 25 µL of PBS, free 3BP, or 3BP@FO. The 3BP dose was 1.7 µg per mouse, and the 3BP@FO formulation contained an equivalent amount of 3BP together with 5 µg of OMV protein. Twenty‐four hours after treatment, tumors were harvested, weighed, and homogenized in PBS. The homogenates were centrifuged, and the resulting supernatants were collected. Lactate concentrations were quantified using an L‐lactate assay kit (Solarbio, China) according to the manufacturer's instructions. Tumor lactate content was calculated according to the formula: lactate (µmol/g tissue) = C × V / m, where C was the measured lactate concentration (µmol/mL), V was the total volume of the tumor homogenate supernatant (mL), and m was the wet weight of the corresponding tumor tissue (g).

### 4.16 Antitumor Activity in Solid Tumor Model

B16 or CT26 tumor cells (5 × 10^5^ cells in 100 µL PBS) were inoculated subcutaneously into female C57BL/6N or BALB/C mice, respectively. When the tumor volume reached approximately 80 mm^3^, mice were randomly assigned to the indicated treatment groups. For monotherapy studies, mice received intratumoral injection of 25 µL of PBS, OMV, Lip, FO, 3BP@OMV, or 3BP@FO (all groups contained an equivalent OMV protein amount of 5 µg). For combination studies, B16‐bearing mice received intratumoral injection of 25 µL PBS, OMV, FO, 3BP@OMV, or 3BP@FO, followed by intraperitoneal administration of αPD‐1 (100 µg per mouse) one day after treatment. Tumor length (L), width (W), and body weight were measured every other day, and tumor volume was calculated using the formula (L×W^2^)/2. Mice were euthanized when the tumor volume exceeded 1500 mm^3^. Heart, liver, spleen, lung, kidney, and tumor tissues were collected, fixed in 4% paraformaldehyde, and sectioned for hematoxylin–eosin (H&E) staining, Ki67, and TUNEL immunohistochemistry. For biosafety evaluation, serum levels of IL‐6, alkaline phosphatase (ALP), alanine aminotransferase (ALT), aspartate aminotransferase (AST), blood urea nitrogen (BUN), lactate dehydrogenase (LDH), and creatinine (CREA) were measured at the experimental endpoint. To evaluate tumor retention and potential off‐target exposure of intratumorally administered OMV‐based formulations, DiR‐labeled OMV and 3BP@FO were intratumorally injected into B16 tumor‐bearing mice. At 72 h post‐injection, mice were euthanized, and tumors together with major organs were collected for ex vivo fluorescence imaging to analyze the distribution of fluorescence signals in each tissue.

### 4.17 Intratumoral Immune Cell Profiling

B16 tumor cells (5 × 10^5^ cells in 100 µL PBS) were subcutaneously inoculated into female C57BL/6N mice. When tumor volumes reached approximately 300 mm^3^, mice were randomly divided into five groups and intratumorally injected with 25 µL of PBS, OMV, Lip, FO, or 3BP@FO (5 µg OMV protein). Forty‐eight hours after treatment, mice were euthanized, and tumors were collected, dissociated, and processed into single‐cell suspensions. The following antibodies were used for flow cytometry staining: FITC‐CD45, APC‐Cy7‐CD11c, PE‐F4/80, and APC‐CD86 for DCs or macrophages; FITC‐CD45, APC‐CD3, Pacific Blue‐CD49b, and PE‐NKG2D for NK cells; FITC‐CD45, APC‐CD3, PE‐CD8, and Pacific Blue‐CD4 for T cells; FITC‐CD45, APC‐CD3, Pacific Blue‐CD4, APC‐Cy7‐CD25, and PE‐Foxp3 for Treg cells; and APC Cy7‐CD45, APC‐CD3, PE‐CD8, BV605‐CD69, FITC‐Ki67, and BV421‐TCF1 for activated (CD69^+^), proliferating (Ki67^+^), and stem‐like (TCF1^+^) T cells. After staining, cells were washed three times with PBS and analyzed by flow cytometry.

### 4.18 Short‐Term Memory Formation and Anti‐Metastasis Evaluation

B16 tumor cells (5×10^5^ cells in 100 µL PBS) were subcutaneously inoculated into female C57BL/6N mice. When the tumor volume reached approximately 100 mm^3^, mice were randomly divided into five groups and intratumorally injected with 25 µL of PBS, OMV, FO, 3BP@OMV, or 3BP@FO (each containing 5 µg vesicle protein). After 48 h, a subset of mice was analyzed for lymph node memory T cell populations using APC‐CD3, PE‐CD8, FITC‐CD44, and Pacific Blue‐CD62L antibodies. Another subset of mice was euthanized 96 h thereafter, and lung tissues were harvested for CD8^+^ T‐cell immunofluorescence staining. The remaining mice received a tail vein injection of B16‐Luc cells (3 × 10^5^ cells in 100 µL PBS). On day 28 post initial tumor inoculation, mice were euthanized, lungs were collected, and imaged using a small animal in vivo imaging system. Lungs were then fixed in 4% paraformaldehyde and subjected to H&E staining, and the number of metastatic nodules was counted.

### 4.19 Long‐Term Memory Formation and Anti‐Recurrence Evaluation

In the long‐term memory model, B16 tumor cells (5×10^5^ cells in 100 µL PBS) were subcutaneously injected into female C57BL/6N mice. When tumors reached approximately 100 mm^3^, mice were divided into five groups and intratumorally injected with 25 µL of PBS, OMV, FO, 3BP@OMV, or 3BP@FO (5 µg OMV protein). On day 15 after tumor inoculation, the primary tumors were surgically removed. On day 35, 5×10^5^ B16 cells were re‐inoculated at the surgical site. Tumor size was recorded. On day 60 after the initial inoculation, mice were euthanized, spleens were collected, and single‐cell suspensions were prepared using PBS. After RBC lysis, cells were stained with PerCP‐CD3, FITC‐CD8, APC‐CD44, and PE‐CD62L antibodies to identify memory T cell populations in the spleen. Tumor tissues were also harvested and subjected to Ki67 staining for histopathological evaluation of tumor proliferative activity.

### 4.20 Statistical Analysis

Data were reported as mean ± standard deviation. Sample sizes (n) and the number of independent biological replicates were specified in the corresponding figure legends. No data were excluded unless predefined experimental exclusion criteria were met. Comparisons between two groups were performed using a two‐tailed unpaired Student's t‐test. Comparisons among multiple groups were performed using one‐way analysis of variance (ANOVA) followed by Tukey's multiple‐comparison test. *p < 0.05; **p < 0.01; ***p < 0.001; ****p < 0.0001; n.s. (not significant). Statistical analyses were performed using GraphPad Prism version 10.6 (GraphPad Software, USA).

## Author Contributions

**W.D.N**., **X.L**., and **J.X.Z**., conceptualized and designed the research. **X.L**., **J.X.Z**., and **F.Z**. performed the experiments. **X.L**., **J.X.Z**., and **F.Z**. collected and processed the data. All authors analyzed and interpreted the data. **X.L**., **J.X**., **W.D.N**., and **H.‐Y.X**. wrote the manuscript. **X.L**. and **J.X.Z**. contributed equally to this work. All authors discussed the results and commented on the manuscript.

## Ethics Approval

All animal experiments were approved by the Animal Ethics Committee of Beijing Institute of Technology (approval no. 2019‐0011‐176) and were conducted in accordance with all relevant ethical guidelines and regulations.

## Conflicts of Interest

The authors declare no conflicts of interest.

## Supporting information




**Supporting File**: advs78279‐sup‐0001‐SuppMat.pdf.

## Data Availability

The data that support the findings of this study are available from the corresponding author upon reasonable request.
