# Supplementary material for: Intratumoral Fusogenic Outer Membrane Vesicles Enhance Antitumor Immunity Through Tumor MHC‐I Upregulation and Tumor Bacterization
Source: Adv Sci (Weinh). 2026 Oct 15:e78279. Online ahead of print. doi: 10.1002/advs.78279 (PMC13672686; doi:10.1002/advs.78279)
Supplement: Supplementary file 1 [file ADVS-9999-e78279-s001.pdf]

Supporting Information

Intratumoral Fusogenic Outer Membrane Vesicles Enhance Antitumor Immunity through Tumor MHC-I Upregulation and Tumor Bacterization

Xing Liu[1], Jiaxin Zhou[1], Gui-Hong Lu[4], Chenjing Zhao[1], Weixiu Wang[1], Jichuan Chen[1], Fengqi Zhang[1], Fan Zhang[3,] *, Hai-Yan Xie[2,] *, Weidong Nie[1,] *

[1] Advanced Technology Research Institute (Jinan), School of Life Science, Beijing Institute of Technology, Beijing, People's Republic of China

[2] State Key Laboratory of Natural and Biomimetic Drugs, School of Pharmaceutical Sciences, Chemical Biology Center, Peking University, Beijing, People's Republic of China

[3] Department of Pathology, Longgang Central Hospital of Shenzhen, Shenzhen, People's Republic of China

[4] Institute of Pediatrics, Shenzhen Children's Hospital, Affiliated Shenzhen Children's Hospital of Shantou University Medical College, Shenzhen, People's Republic of China

E-mail: hljszf@163.com; hyanxie@bjmu.edu.cn; victornia@bit.edu.cn

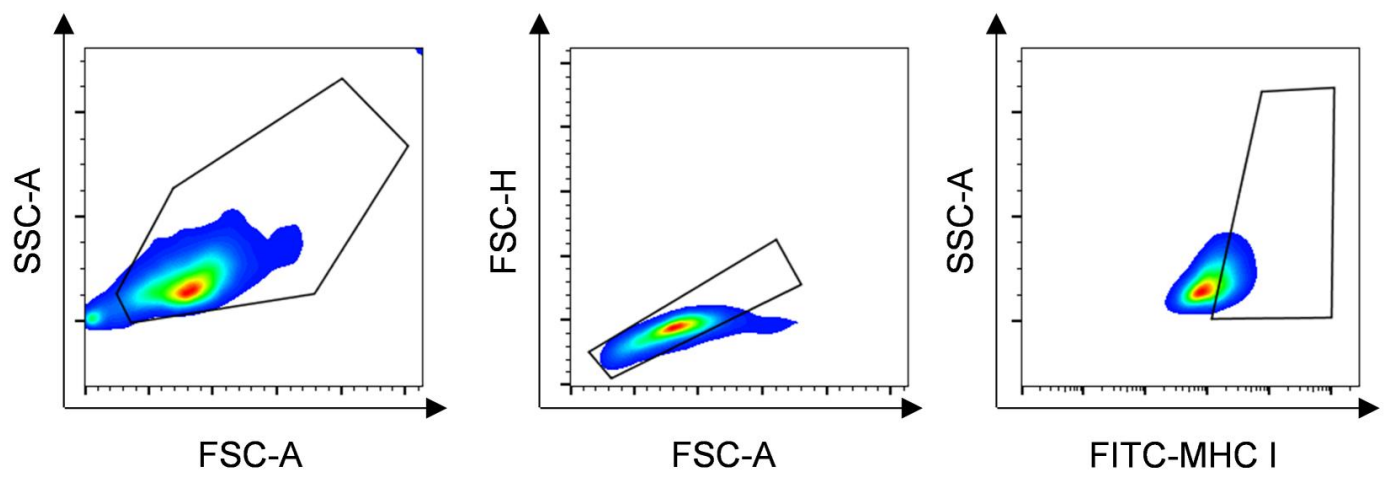

Fig. S1. Gating strategy for flow cytometry analysis of MHC-I⁺ B16 tumor cells.

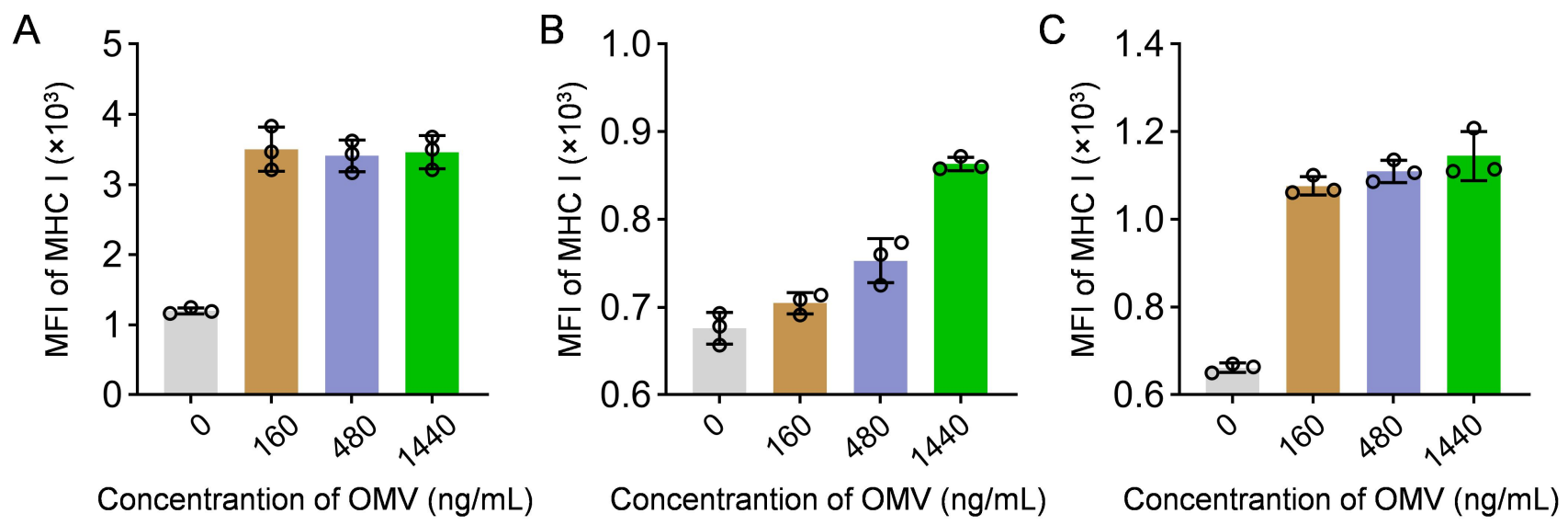

Fig S2. Quantification of the mean fluorescence intensity (MFI) of MHC-I on B16 (A), MCF-7(B), and A549 (C) tumor cells. Data are shown as mean ± SD (n = 3).

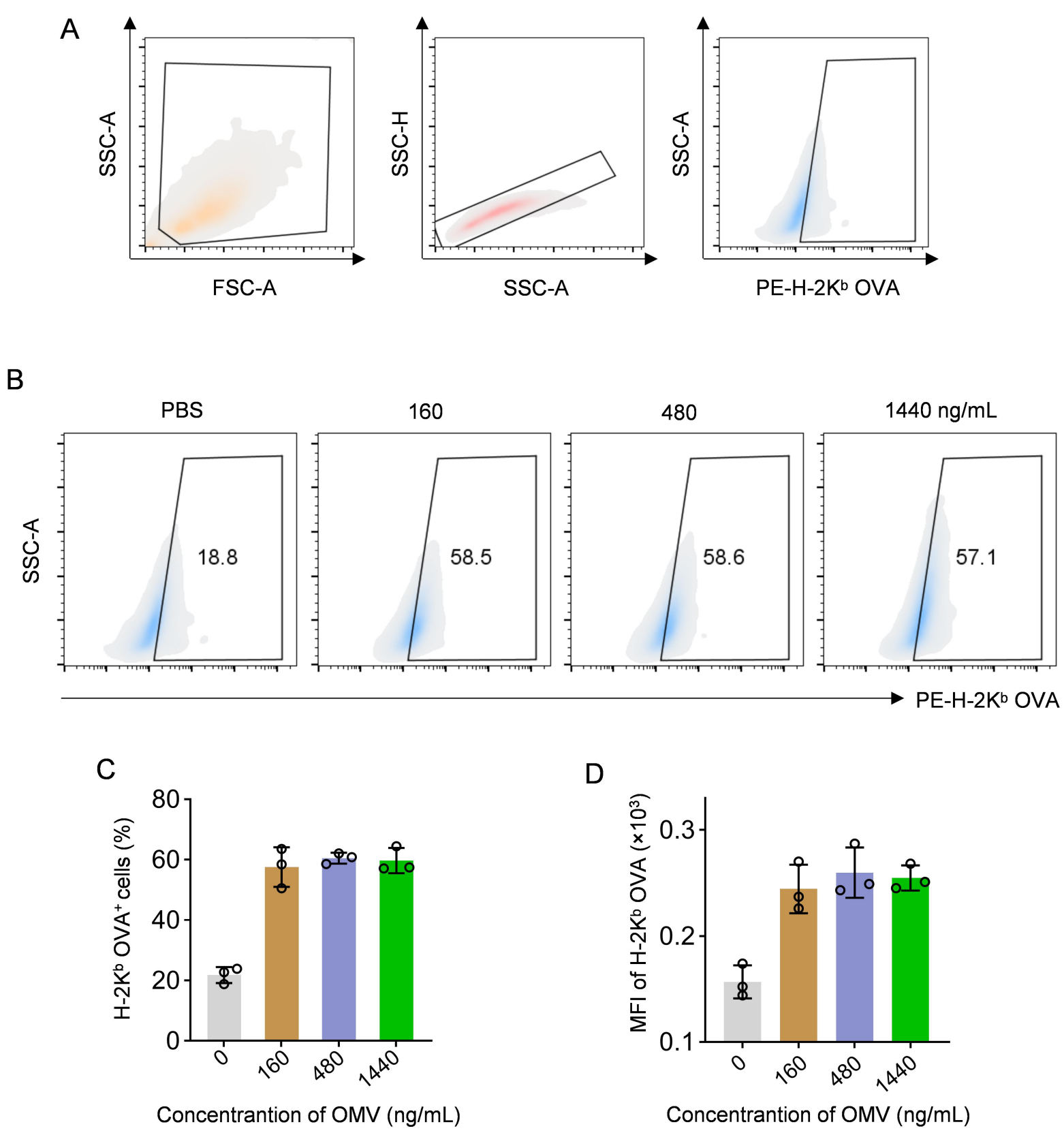

Fig S3. (A) Gating strategy for flow cytometry analysis of H-2K^b OVA$^+$ tumor cells. (B) Representative flow cytometry plots. (C) Quantification of H-2K^b OVA$^+$ tumor cells. (D) Quantification of the MFI of H-2K^b OVA on tumor cells. Data are mean ± SD (n = 3).

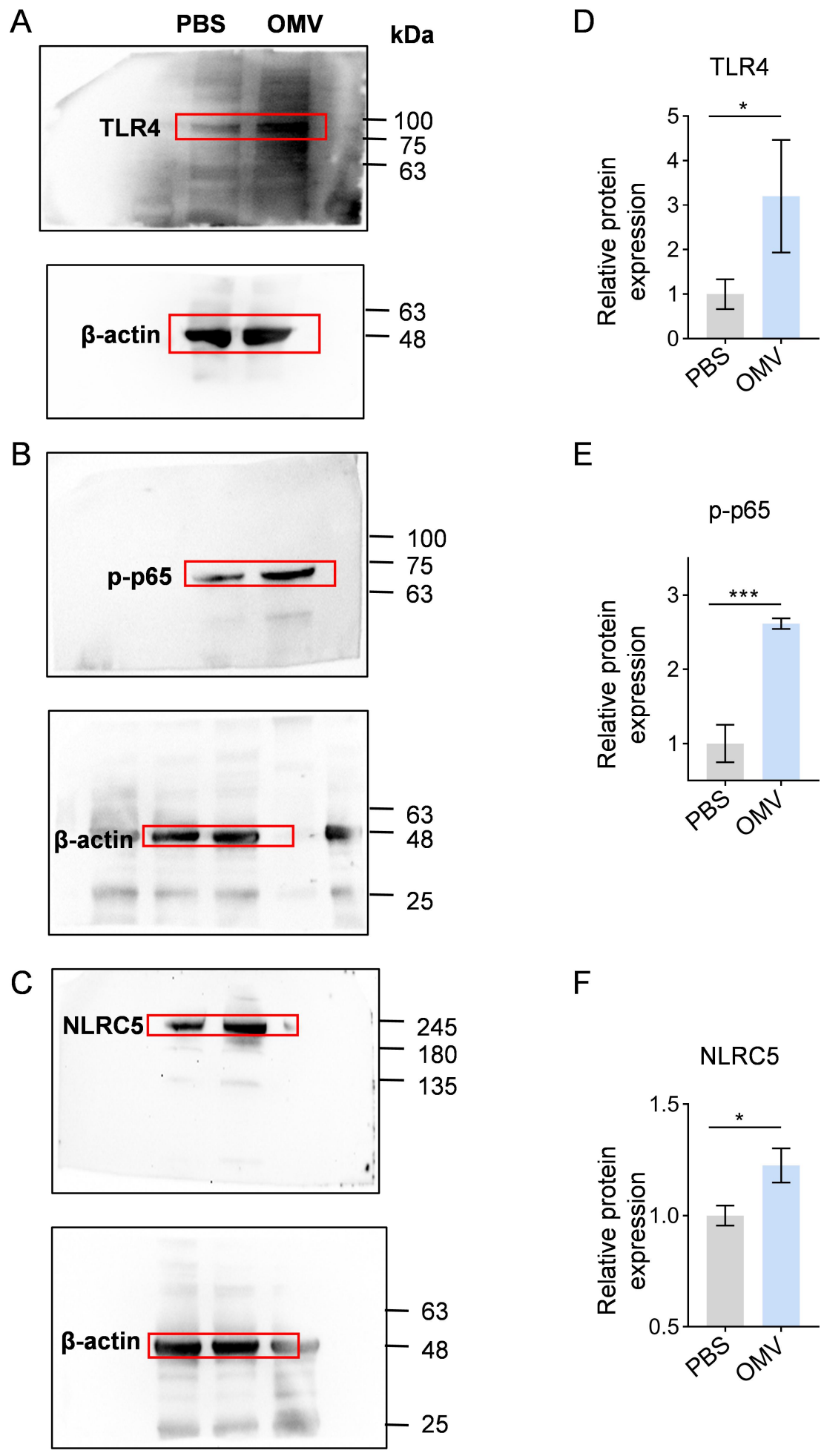

Fig. S4. (A–C) Representative Western blot images of TLR4 (~100 kDa), p-p65 (~70 kDa), NLRC5 (~225 kDa), and β-actin (~48 kDa) in tumor cells. (D–F) Relative densitometric quantification of TLR4, p-p65, and NLRC5. Protein levels were normalized to β-actin and expressed relative to the control group. Data are presented as mean ± SD (n = 3). Statistical significance was determined using Student's t-test.*P < 0.05, ***P < 0.001.

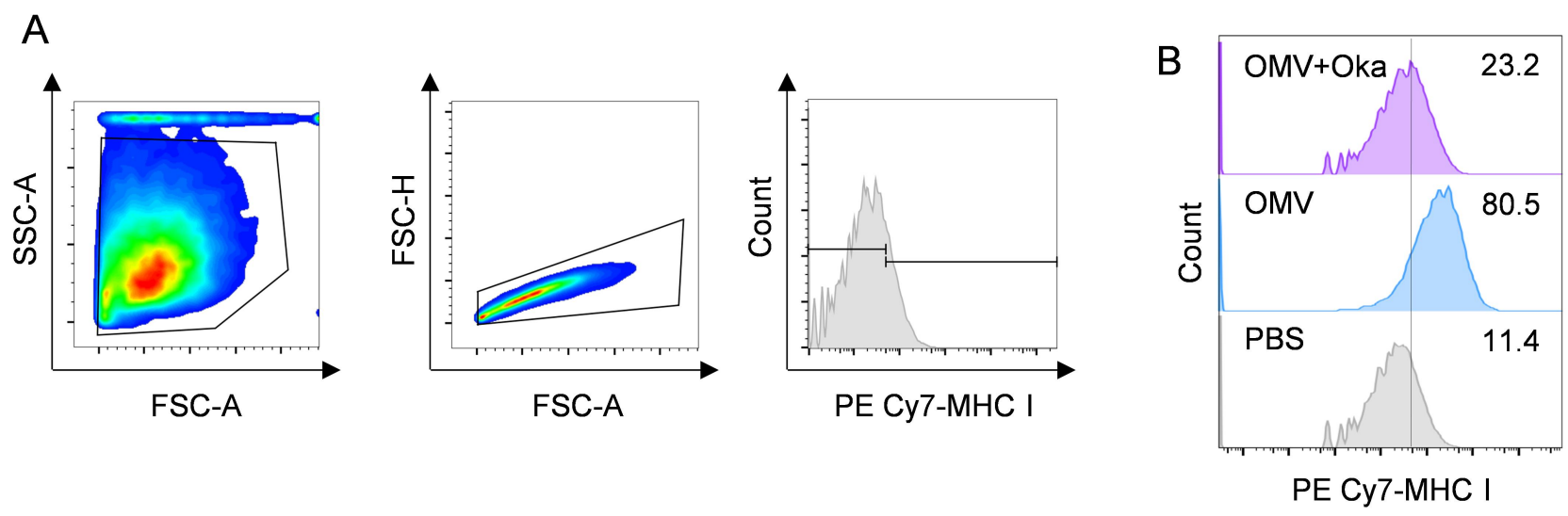

Fig. S5. (A) Gating strategy and (B) representative histograms for flow cytometry analysis of MHC-I$^+$ B16 tumor cells.

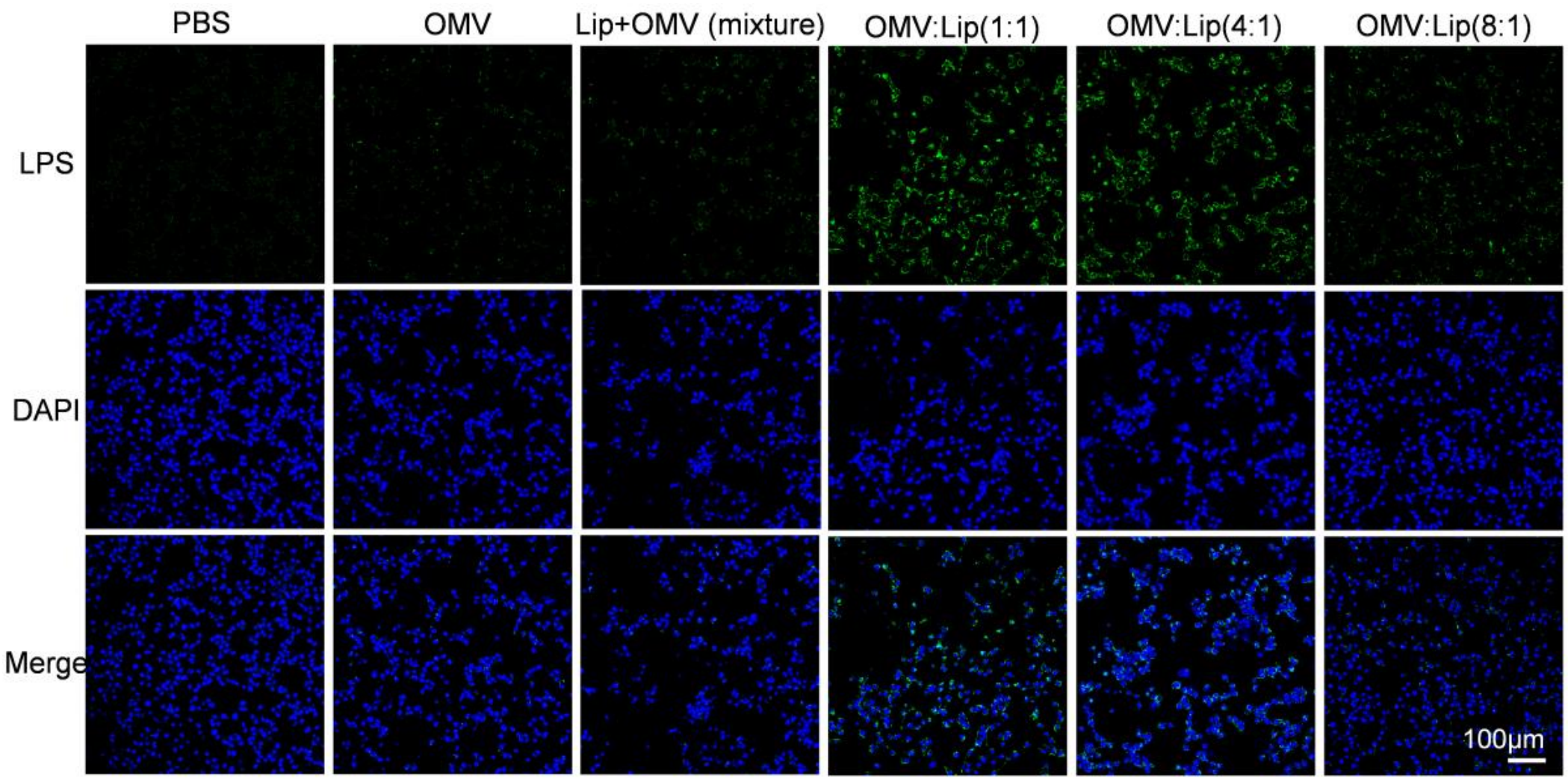

Fig. S6. Confocal laser scanning microscopy (CLSM) images of B16 cells treated with different FO formulations. Green, antibody-labeled LPS; blue, DAPI-labeled nuclei. The Lip+OMV group represents a physical mixture of liposomes and OMVs at a mass ratio of 1:1.

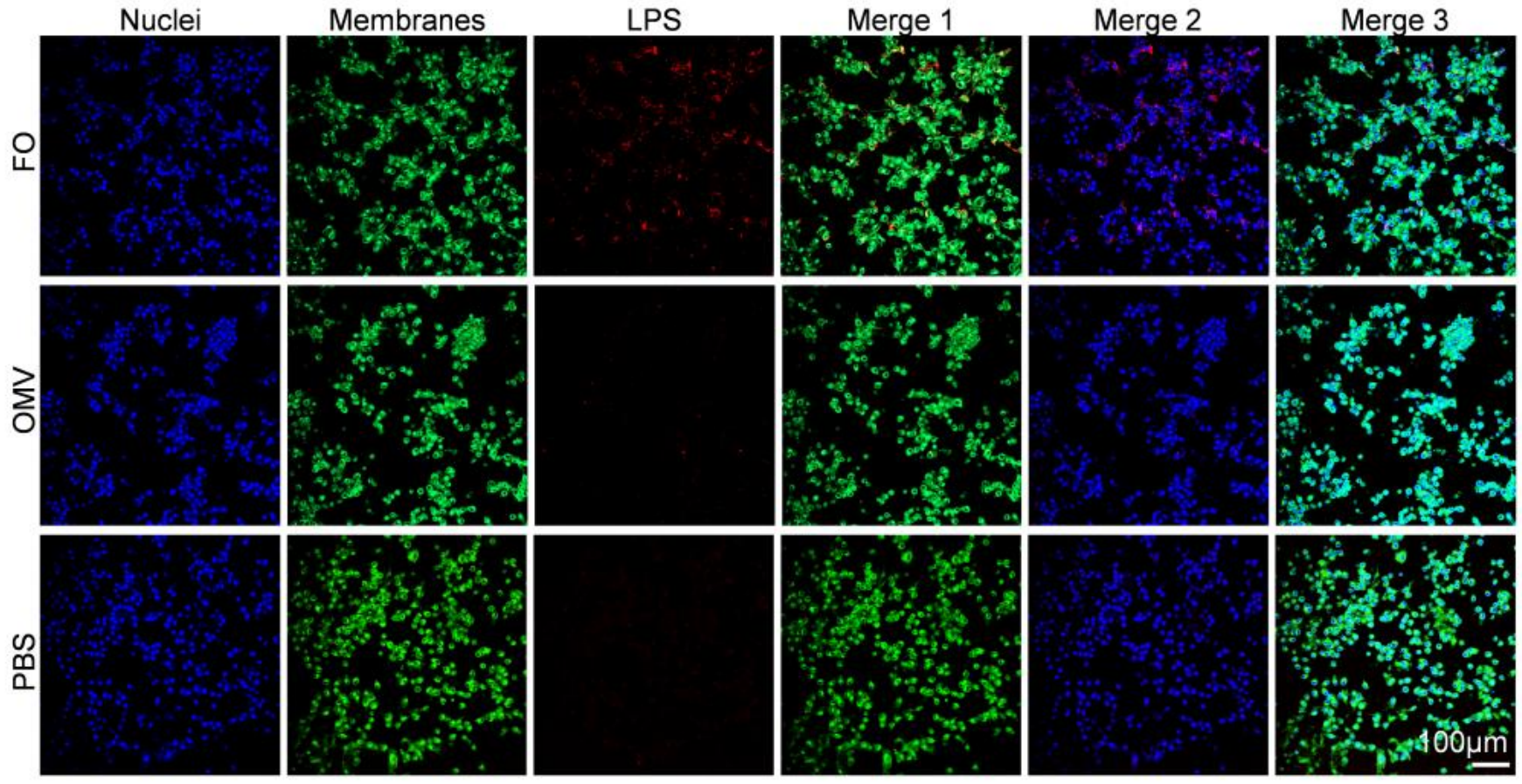

Fig. S7. Confocal laser scanning microscopy (CLSM) images of B16 cells treated with PBS, OMV, and FO. Green, WGA-labeled membrane; red, antibody-labeled LPS; blue, DAPI-labeled nuclei.

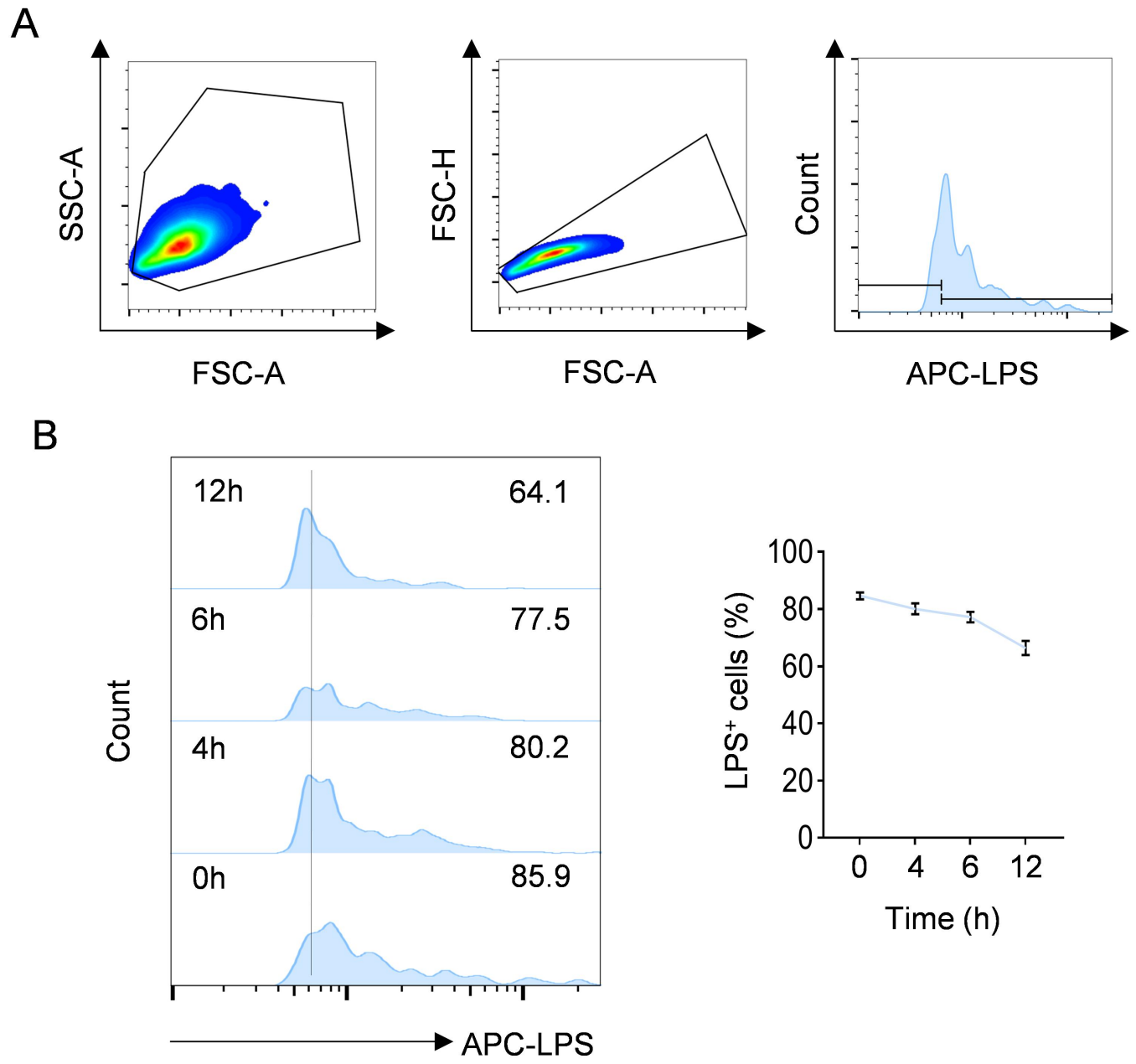

Fig S8. Residence time of OMVs on cell membranes. (A) Gating strategy. (B) Representative flow cytometry histograms and quantification of LPS^+ B16 cells at the indicated time points. Data are mean ± SD (n = 3).

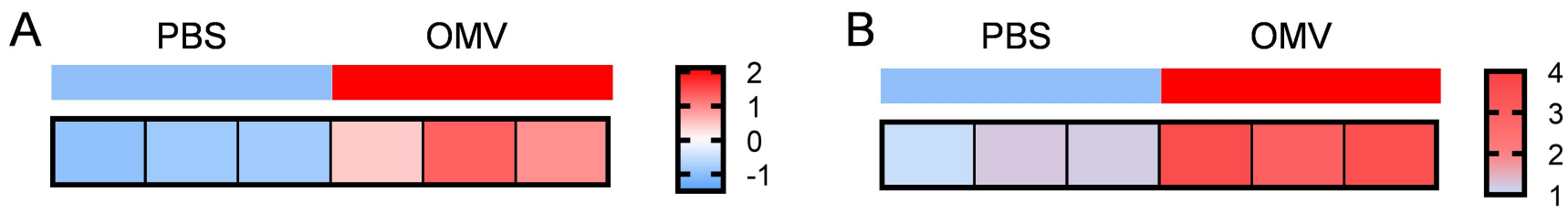

Fig S9. Heat maps of CCL5 genes detected by RNA-seq (A) and qPCR (B).

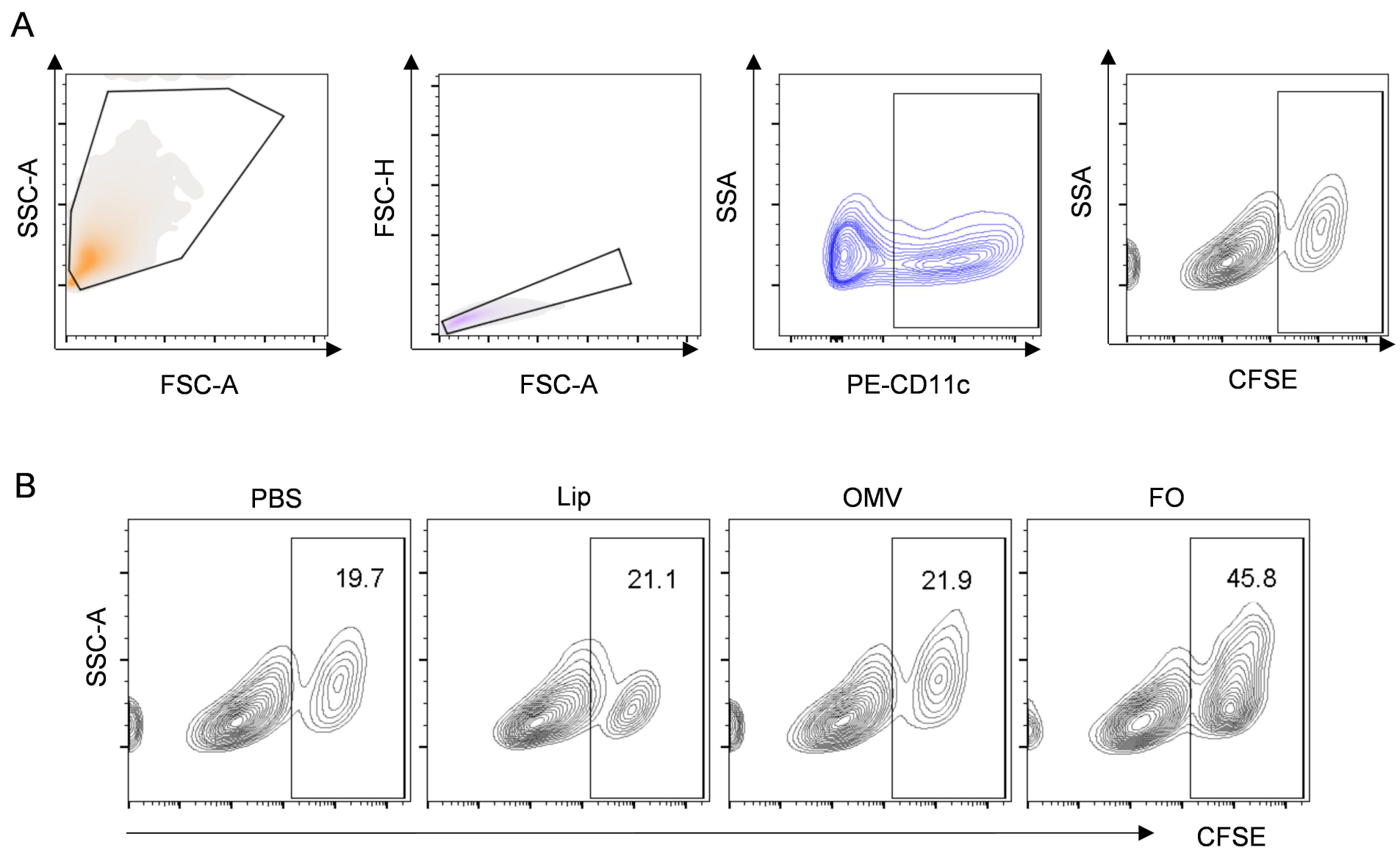

Fig S10. (A) Gating strategy and (B) representative plots for flow cytometry analysis of phagocytosis of CFSE-labeled tumor cells by DCs ($CD11c^+$) .

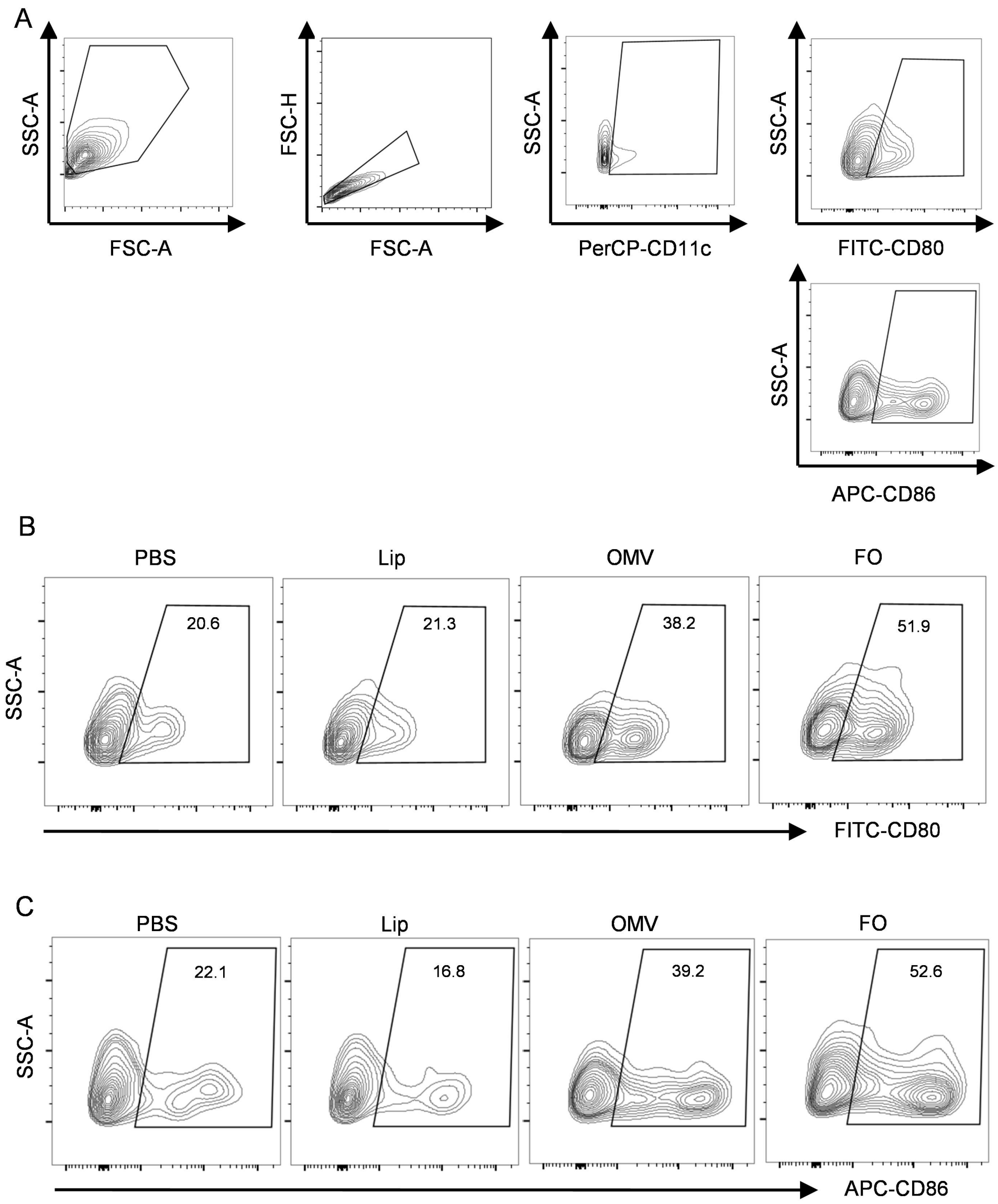

Fig S11. (A) Gating strategy and (B,C) representative plots for flow cytometry analysis of CD80⁺ and CD86⁺ DCs following the indicated treatments.

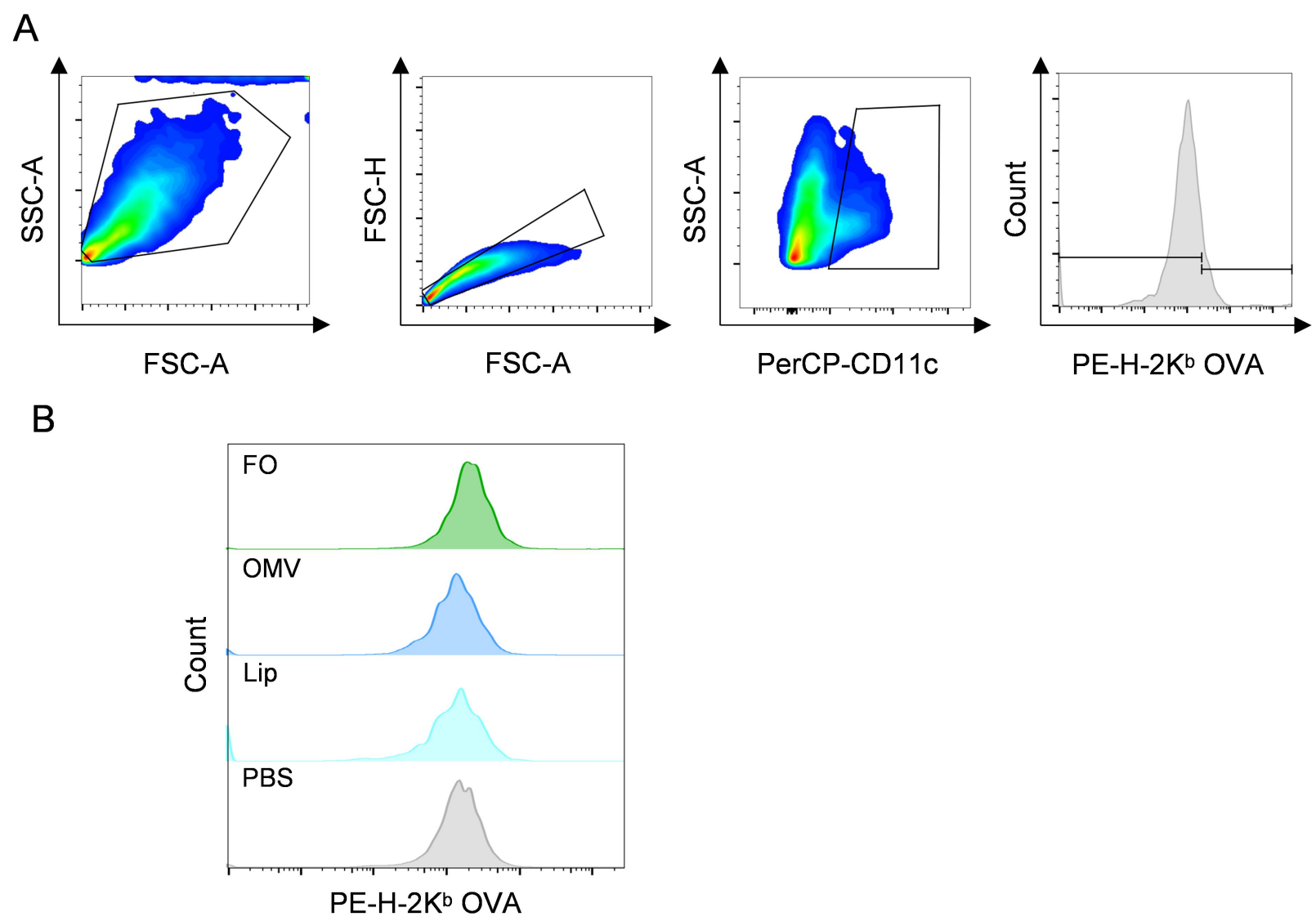

Fig S12. (A) Gating strategy and (B) representative histograms for flow cytometry analysis of H-2K^b OVA$^+$ DCs following the indicated treatments.

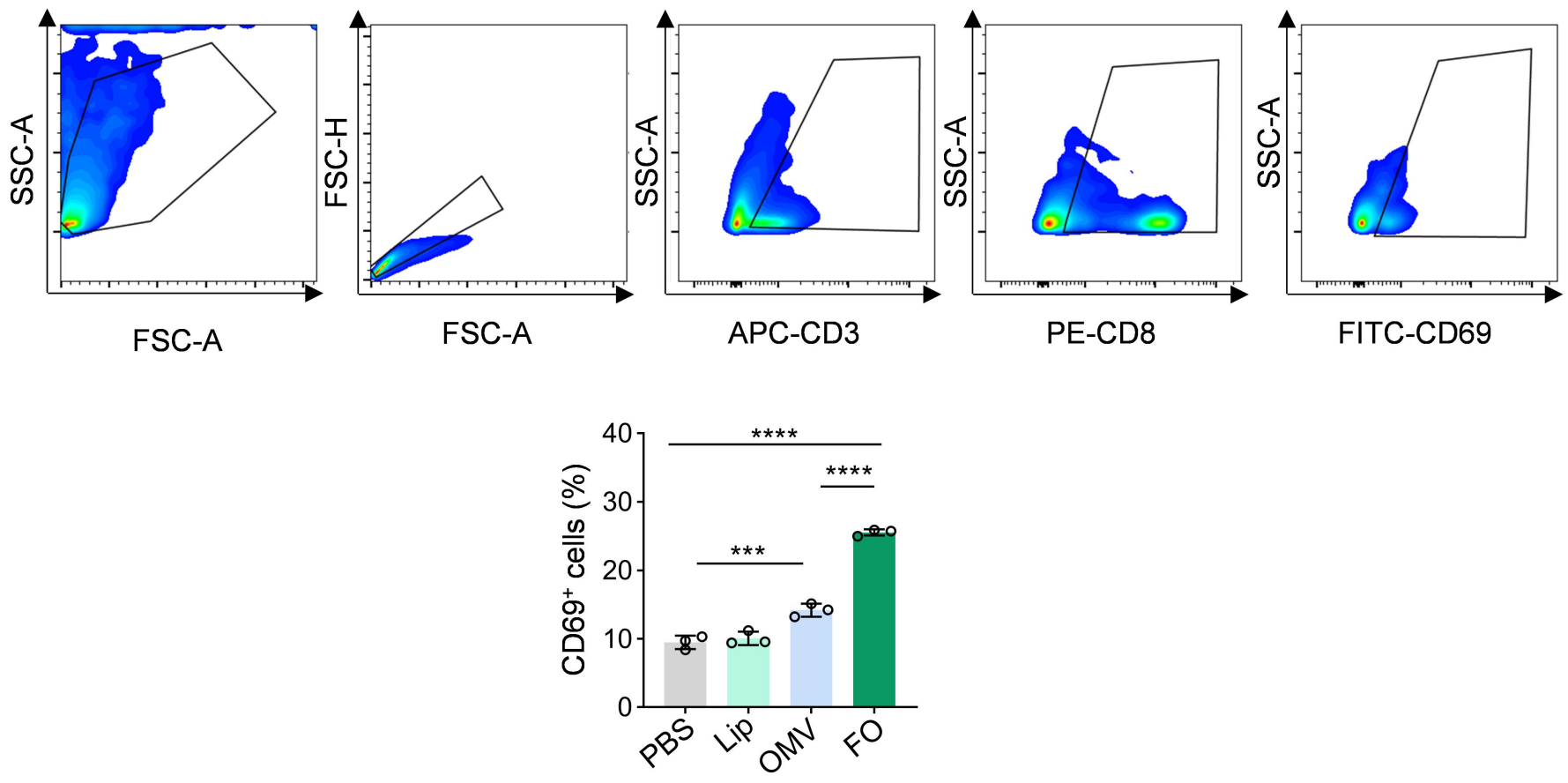

Fig S13. Gating strategy and quantification for flow cytometry analysis of CD69⁺ T cells. Data are mean ± SD (n = 3). Statistical significance was determined using One-way ANOVA. ****P < 0.0001, ***P < 0.001.

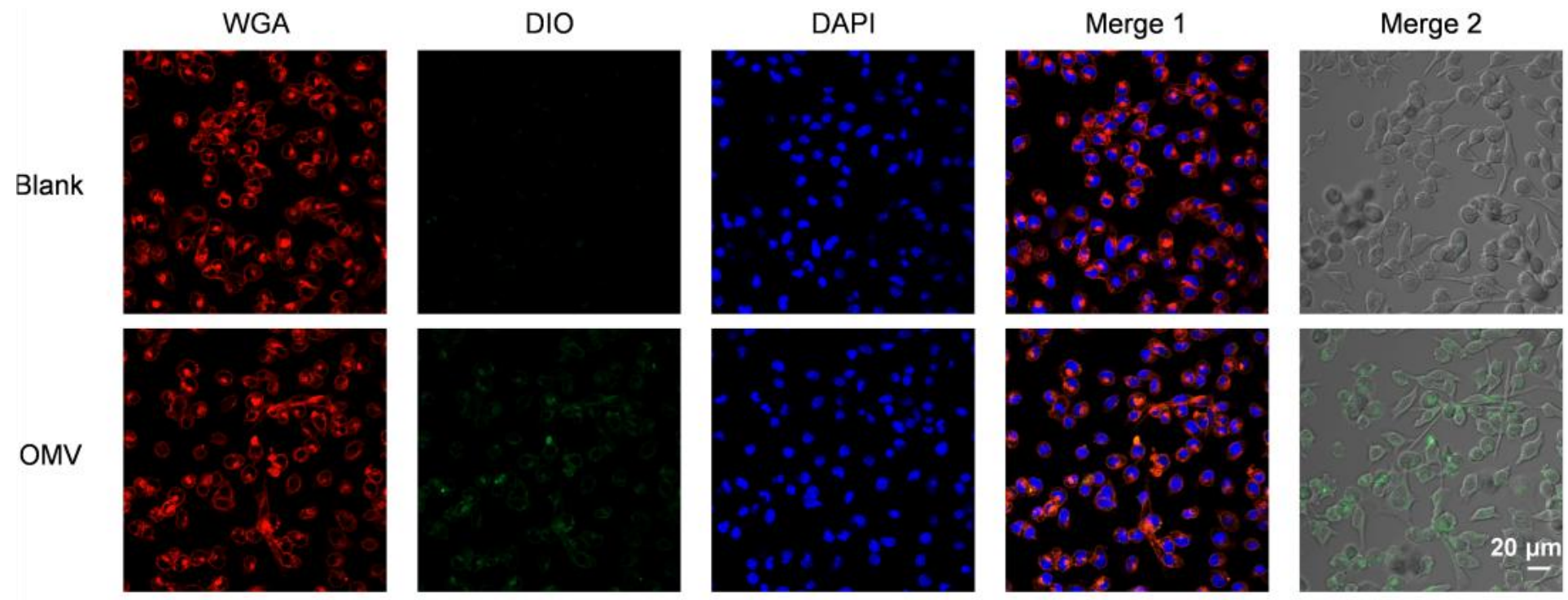

Fig S14. CLSM images of B16 cells treated with OMVs for 20 minutes. Red, WGA-labeled cell membranes; green, DiO-labeled OMVs; blue, DAPI-labeled nuclei.

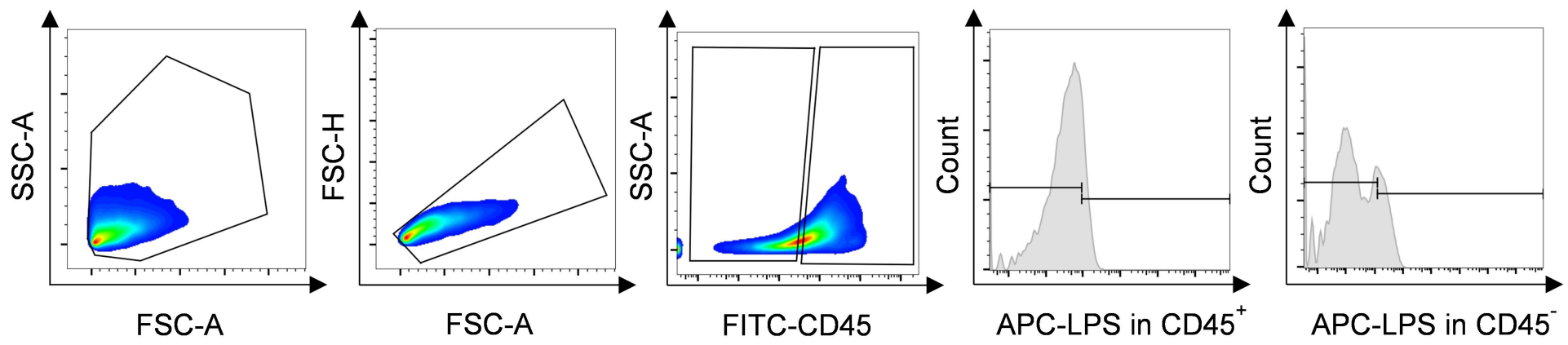

Fig S15. Gating strategy for flow cytometry analysis of LPS+ tumor cells following the indicated treatments.

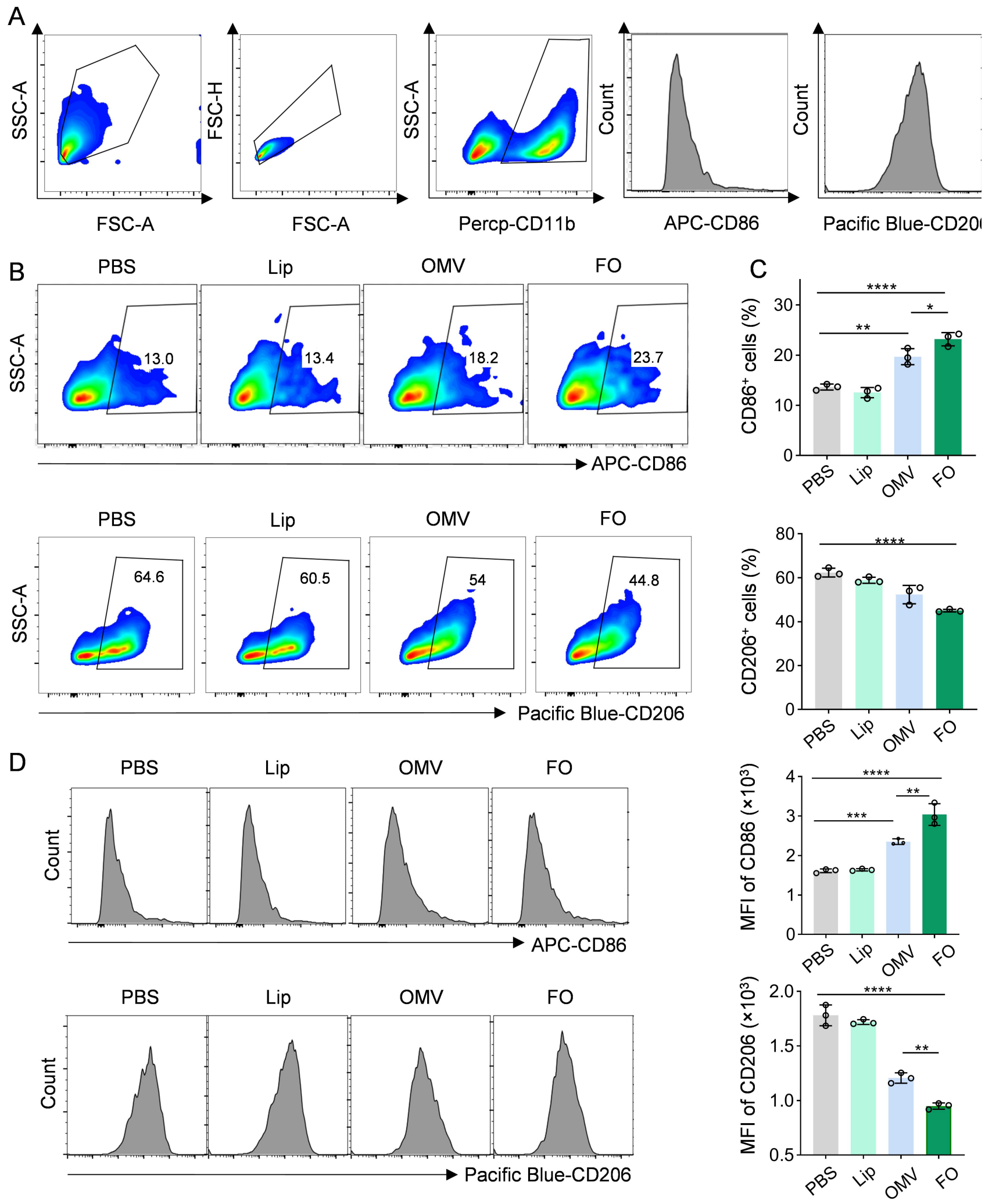

Fig S16. (A) Gating strategy, (B) representative plots, and (C) quantification for flow cytometry analysis of CD86⁺ and CD206⁺ macrophages, and the corresponding MFI (D) of CD86 and CD206 on tumor-associated macrophages following the indicated treatments. Data are mean ± SD (n=3). Statistical significance was determined using Student's t-test or One-way ANOVA. ****P<0.0001, ***P<0.001, **P<0.01, *P<0.05.

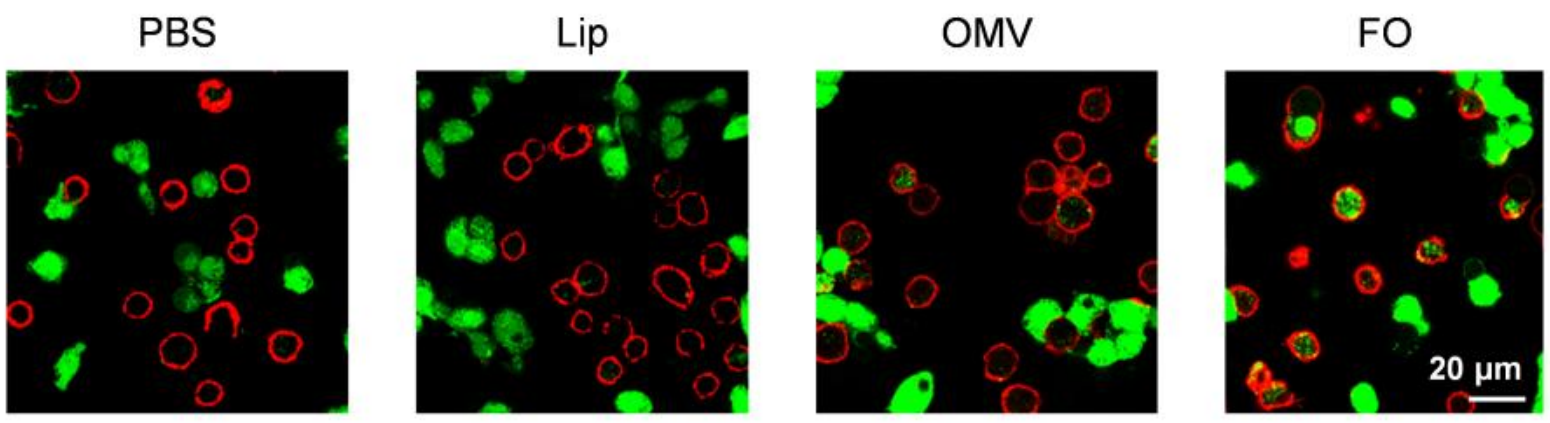

Fig S17. Fluorescence images of the phagocytosis of tumor cells (green, CFSE-labeled) by macrophages (red, CD11b antibody-labeled) .

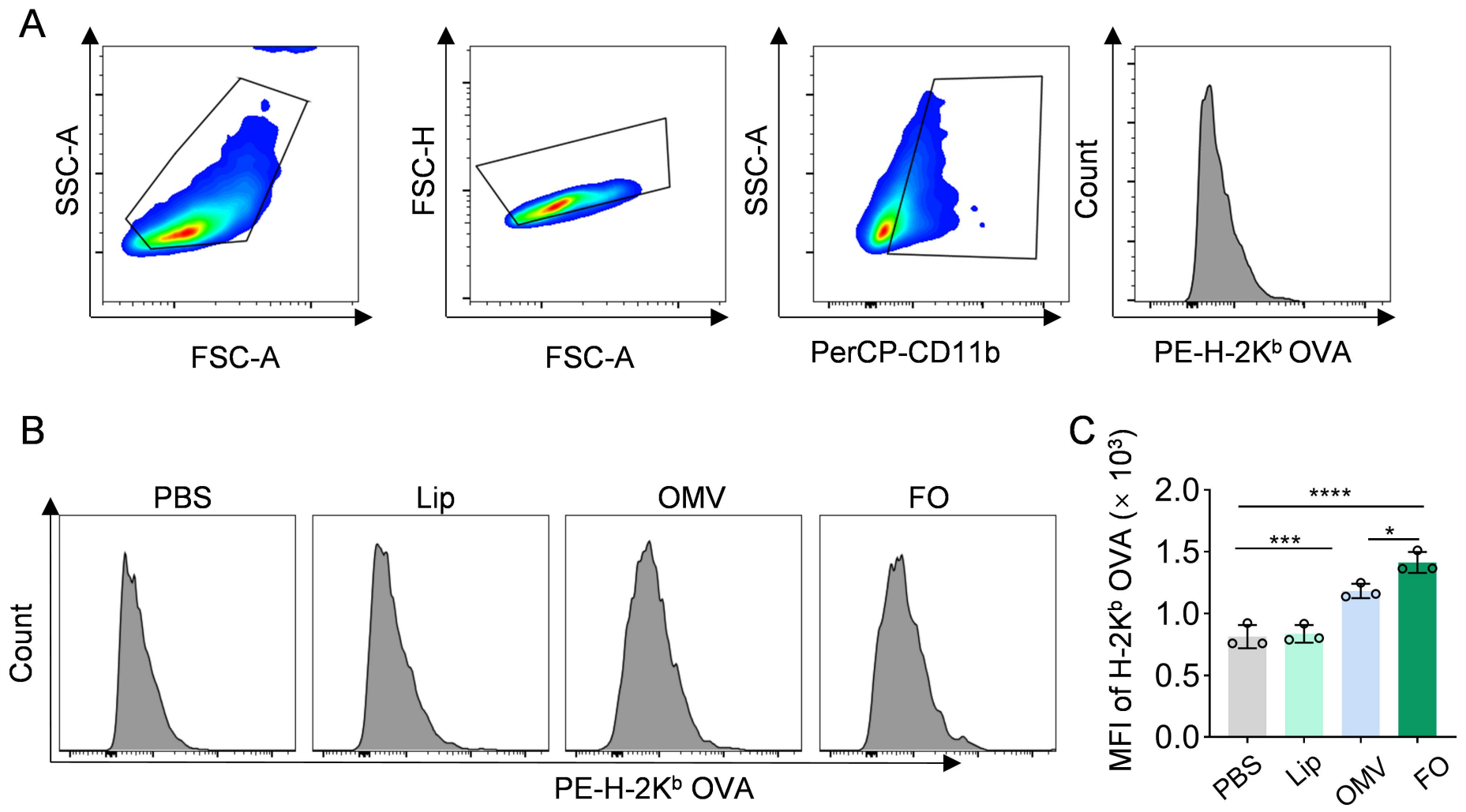

Fig S18. (A,B) Gating strategy and representative histograms for flow cytometry analysis of tumor antigen presentation by macrophages following the indicated treatments. (C) Quantification of the MFI of H-2K^b OVA on CD11b+ macrophages. Data are mean ± SD (n=3). Statistical significance was determined using One-way ANOVA. ****P<0.0001, ***P<0.001, *P<0.05.

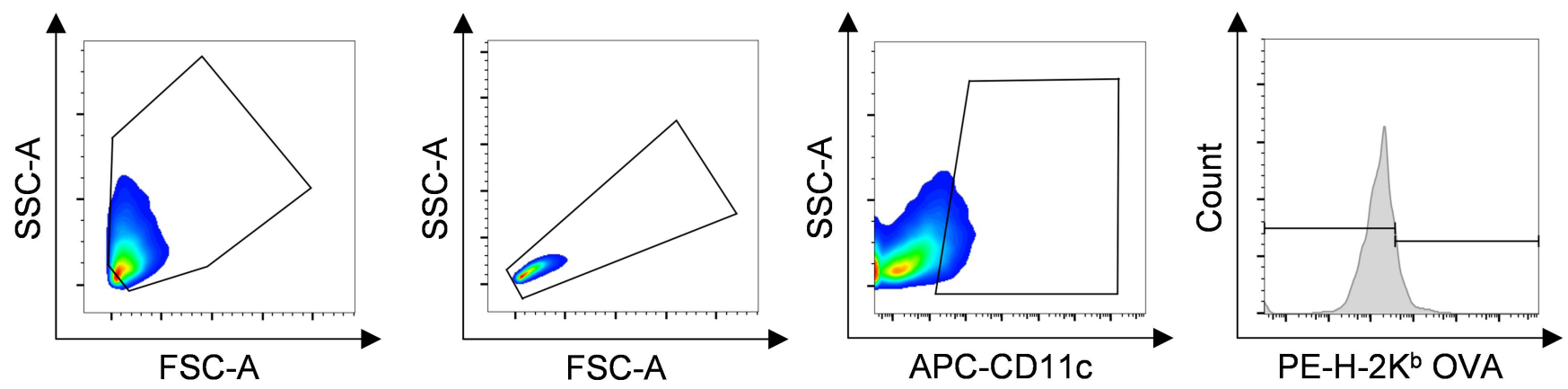

Fig S19. Gating strategy for flow cytometry analysis of DC antigen presentation after M2 macrophage co-culture.

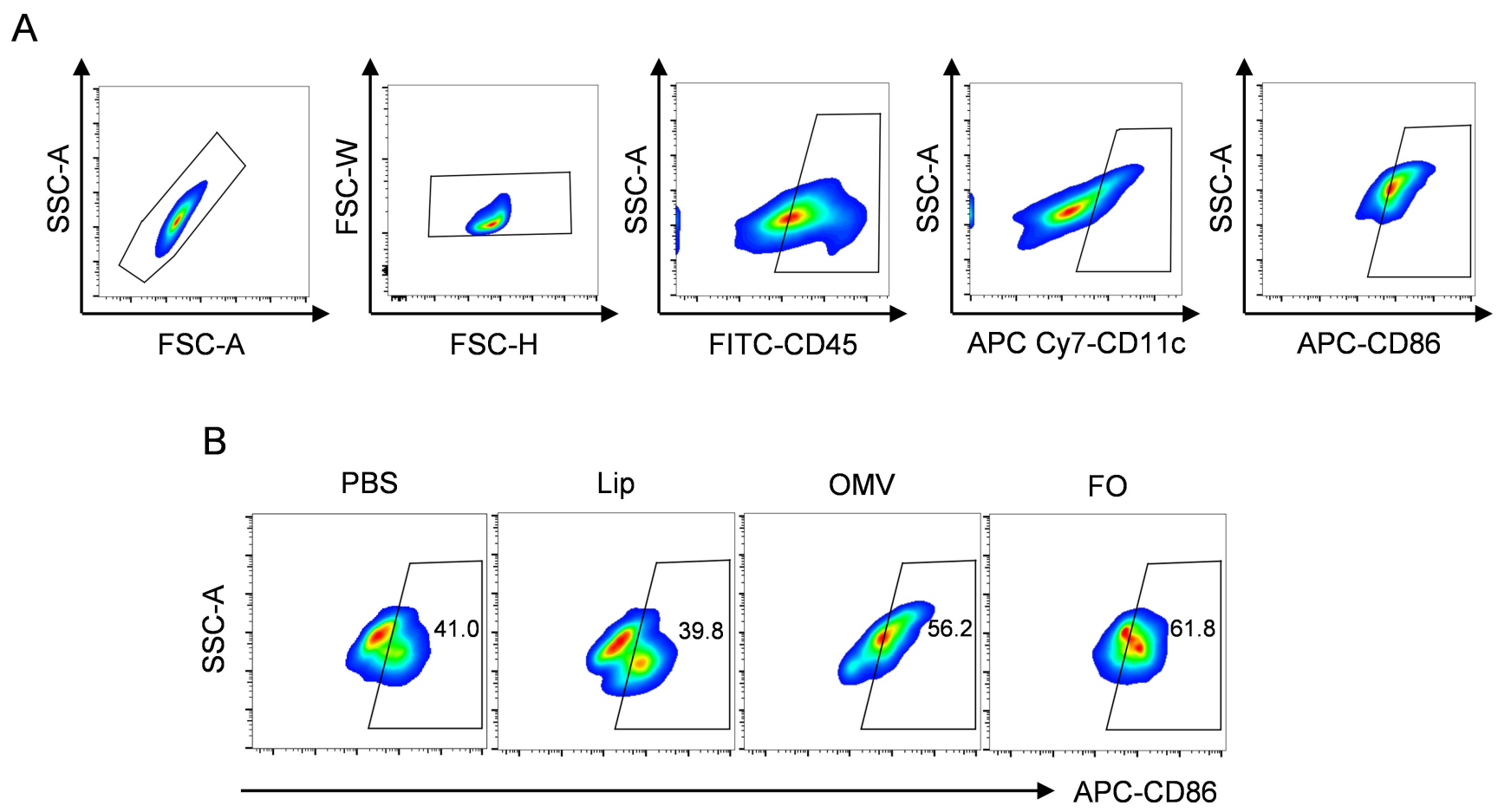

Fig S20. (A) Gating strategy and (B) representative plots for flow cytometry analysis of CD86^{+} DCs (CD11c^{+}) in the TME following the indicated treatments.

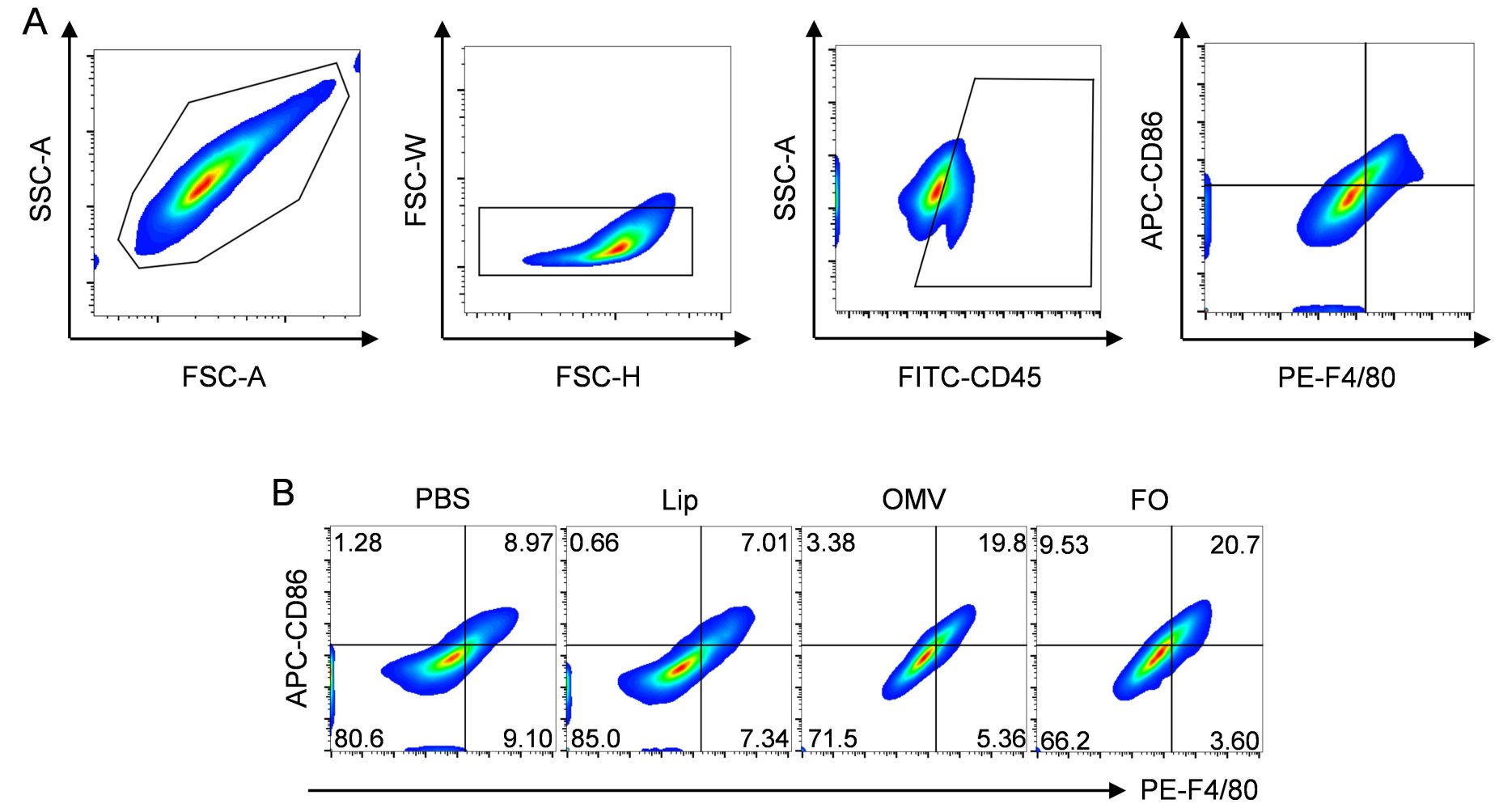

Fig S21. (A) Gating strategy and (B) representative plots for flow cytometry analysis of F4/80⁺CD86⁺ macrophages in the TME following the indicated treatments.

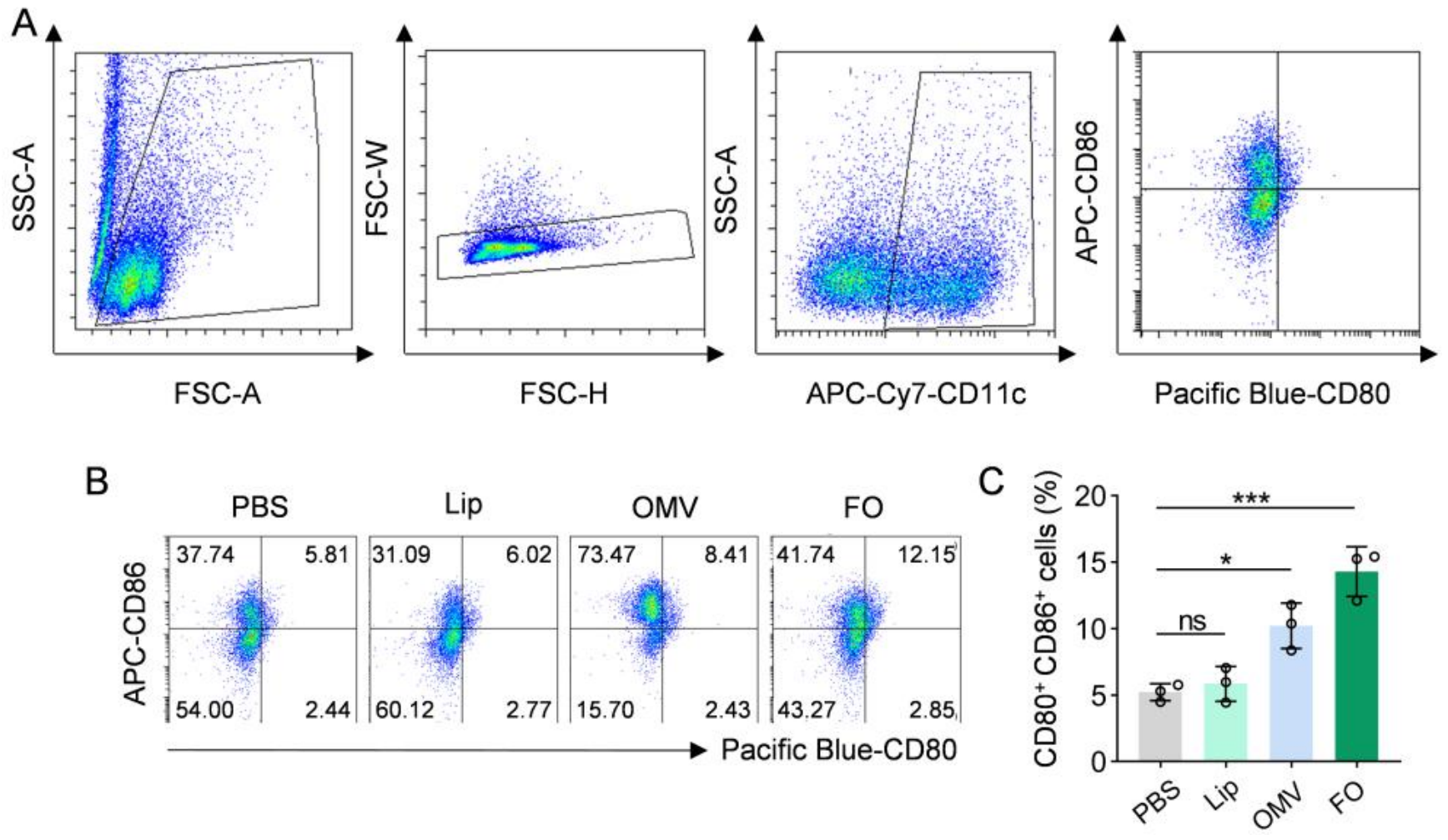

Fig S22. (A–C) Gating strategy, representative plots, and quantification for flow cytometry analysis of $CD80^{+}CD86^{+}$ DCs in lymph nodes following the indicated treatments. Data are mean ± SD (n = 3). Statistical significance was determined using Student's t-test or One-way ANOVA. ***P < 0.001, *P < 0.05; n.s., not significant.

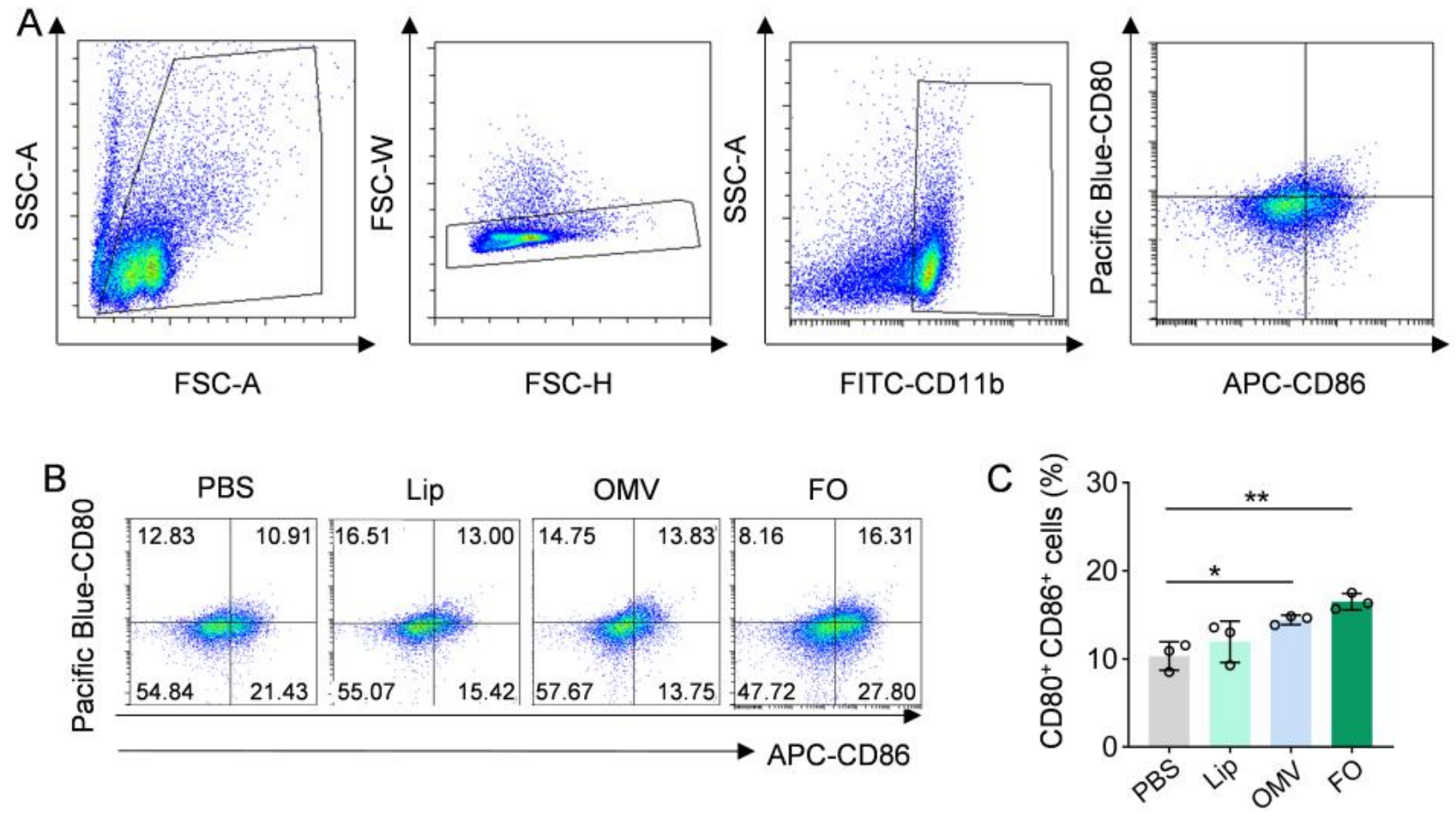

Fig S23. (A–C) Gating strategy, representative plots, and quantification for flow cytometry analysis of $CD80^{+}CD86^{+}$ macrophages in lymph nodes following the indicated treatments. Data are mean ± SD (n = 3). Statistical significance was determined using One-way ANOVA. **P < 0.01, *P < 0.05.

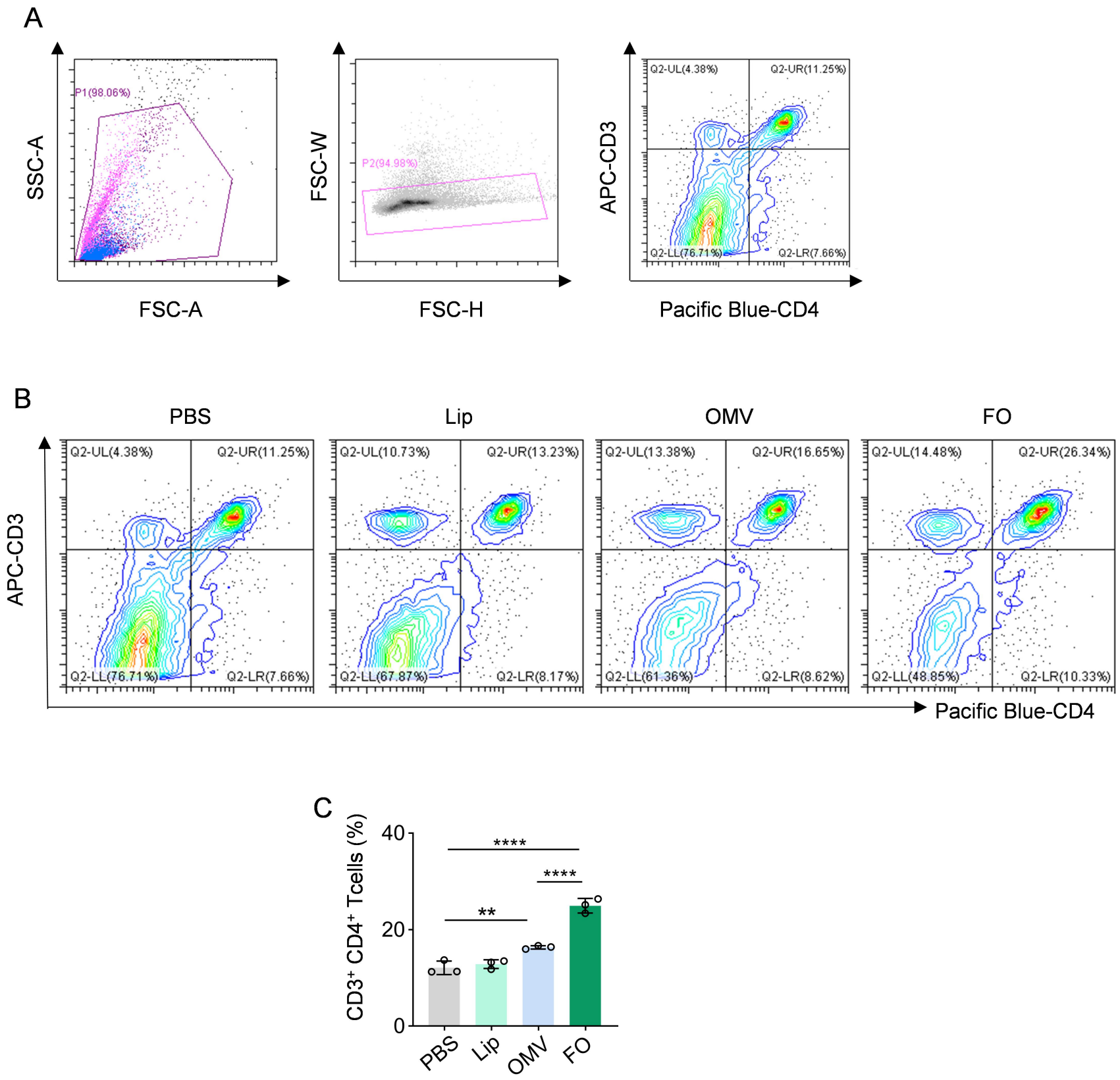

Fig S24. (A–C) Gating strategy, representative plots, and quantification for flow cytometry analysis of $CD3^{+}CD4^{+}$ T cells in lymph nodes following the indicated treatments. Data are mean ± SD (n = 3). Statistical significance was determined using One-way ANOVA. ****P < 0.0001, **P < 0.01.

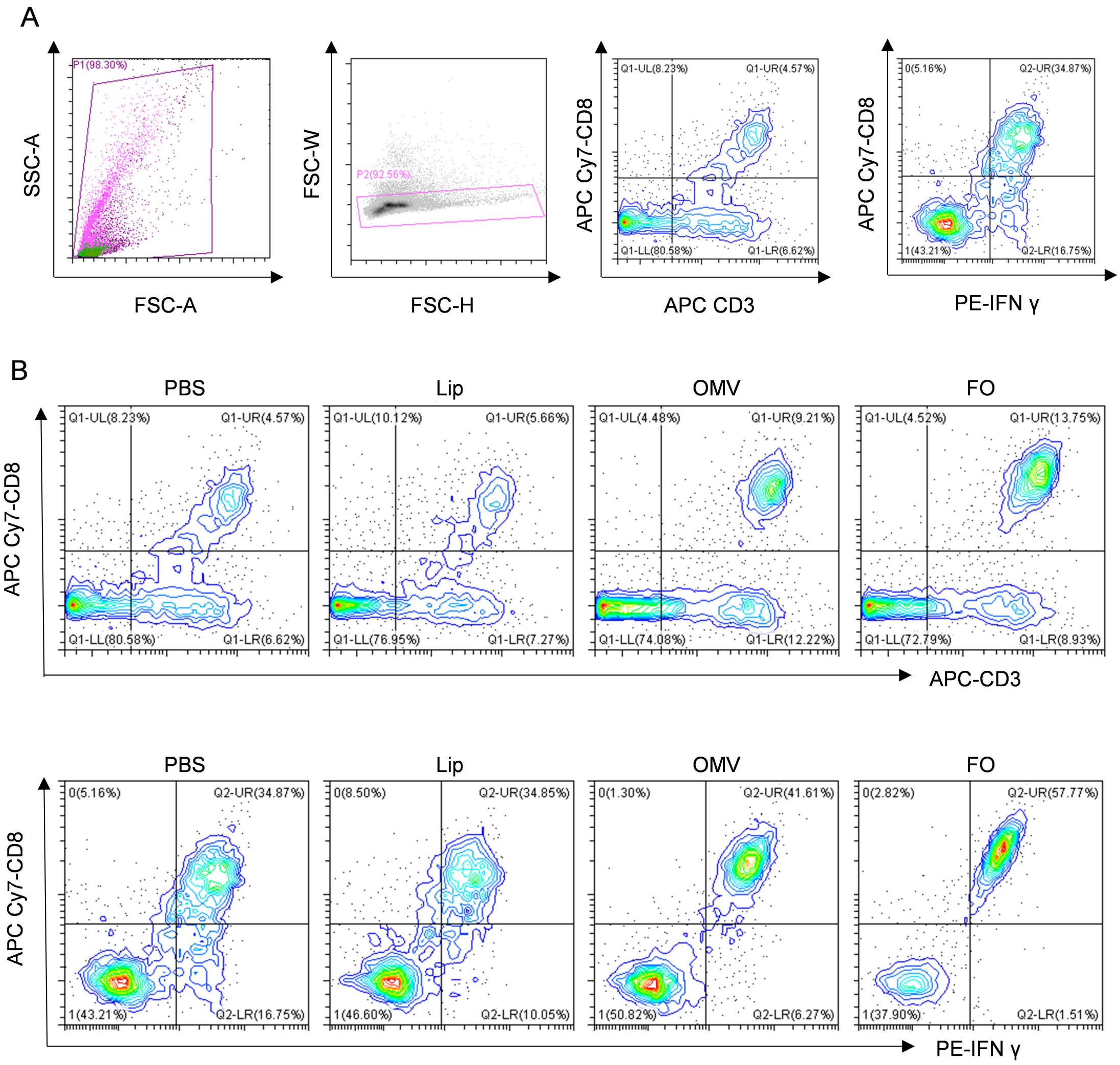

Fig S25. (A–B) Gating strategy and representative plots for flow cytometry analysis of $CD3^{+}CD8^{+}$ and $CD8^{+}IFN\gamma^{+}$ T cells in lymph nodes following the indicated treatments.

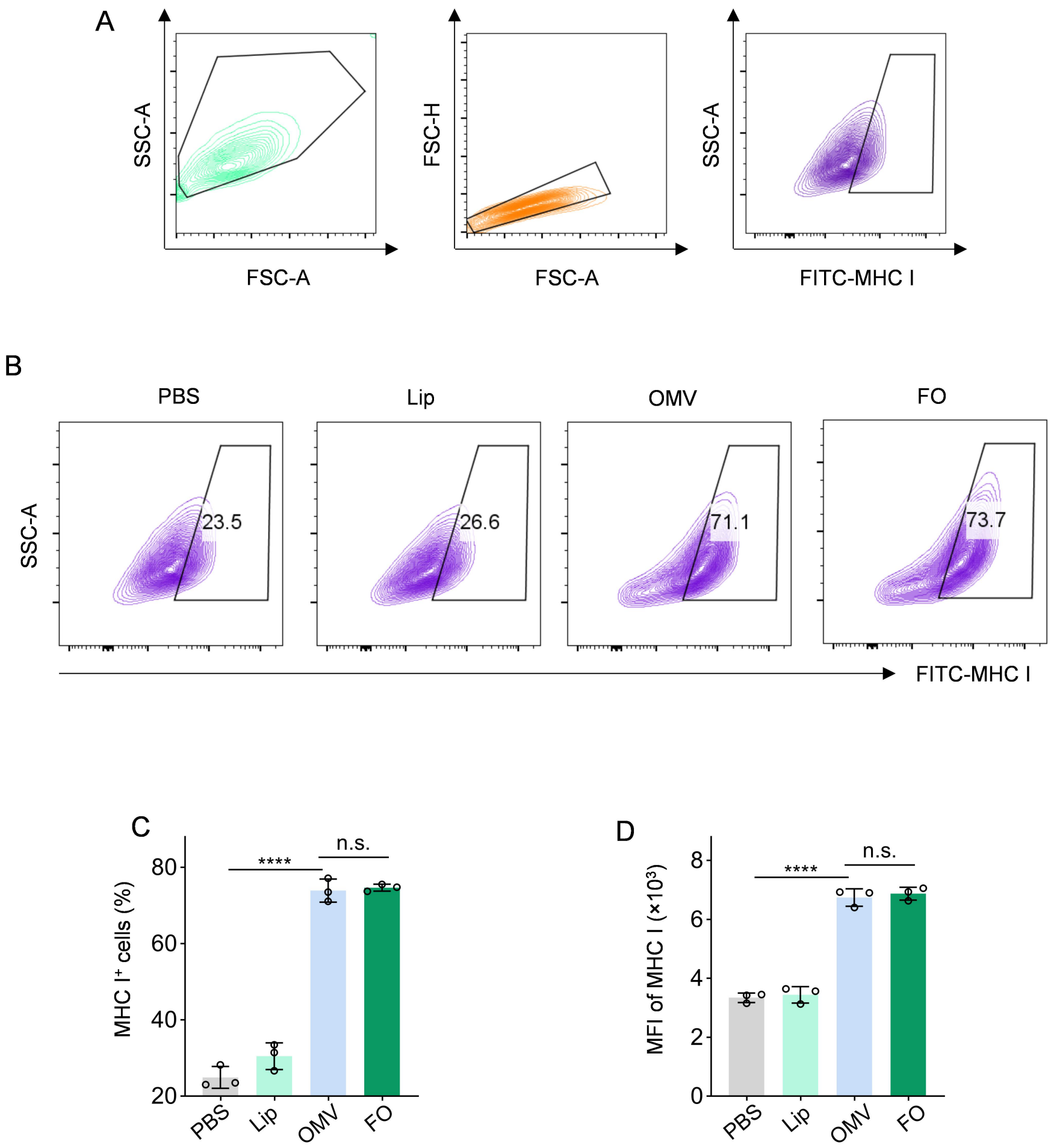

Fig S26. (A–C) Gating strategy, representative plots, and quantification for flow cytometry analysis of MHC-I$^+$ tumor cells. (D) MFI of MHC-I on tumor cells. Data are mean ± SD (n=3). Statistical significance was determined using One-way ANOVA. ****P<0.0001; n.s., not significant.

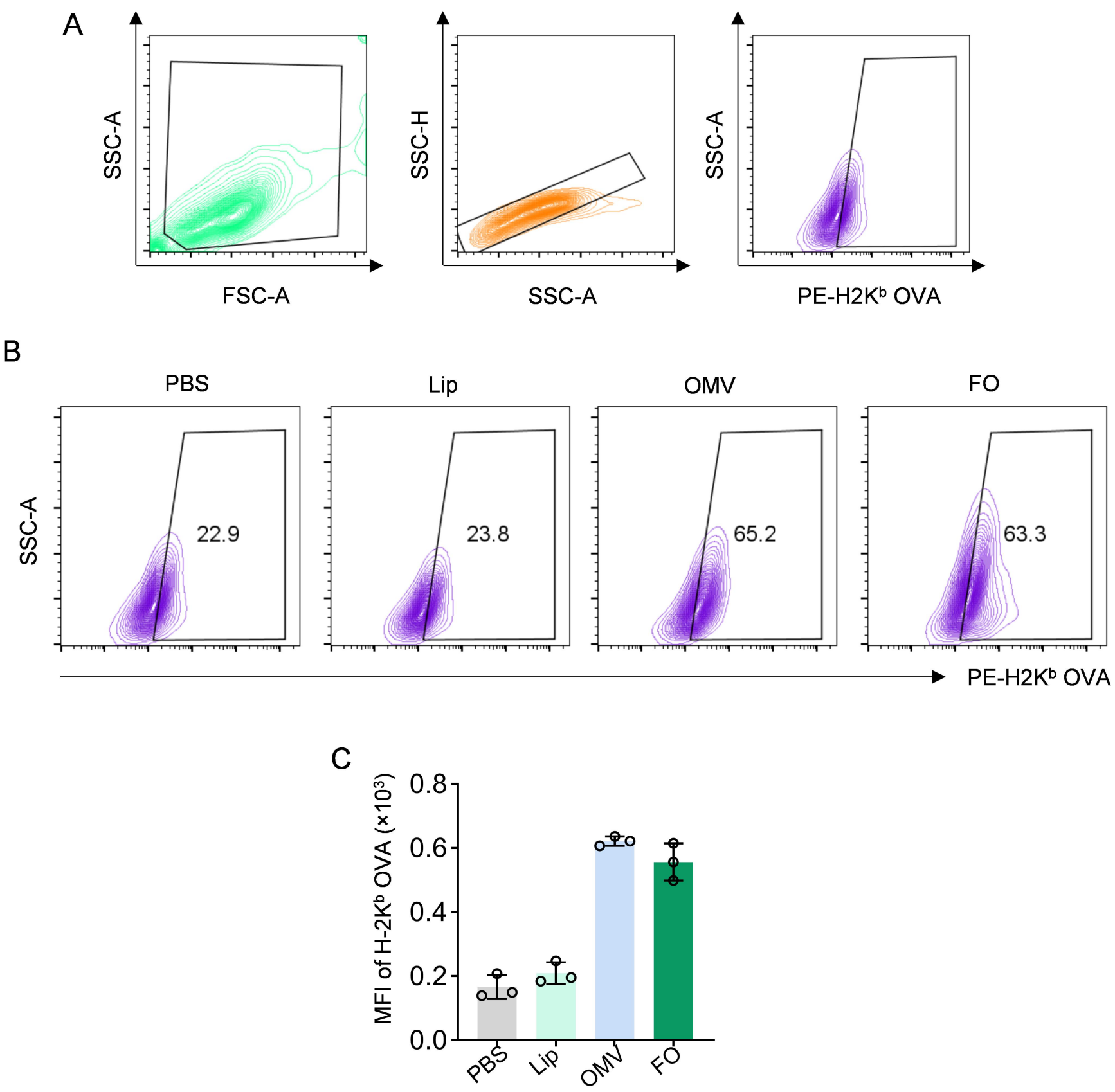

Fig S27. (A–B) Gating strategy, representative plots, and quantification for flow cytometry analysis of H-2K^b OVA$^+$ tumor cells. (C) MFI of H-2K^b OVA on tumor cells. Data are mean ± SD (n=3).

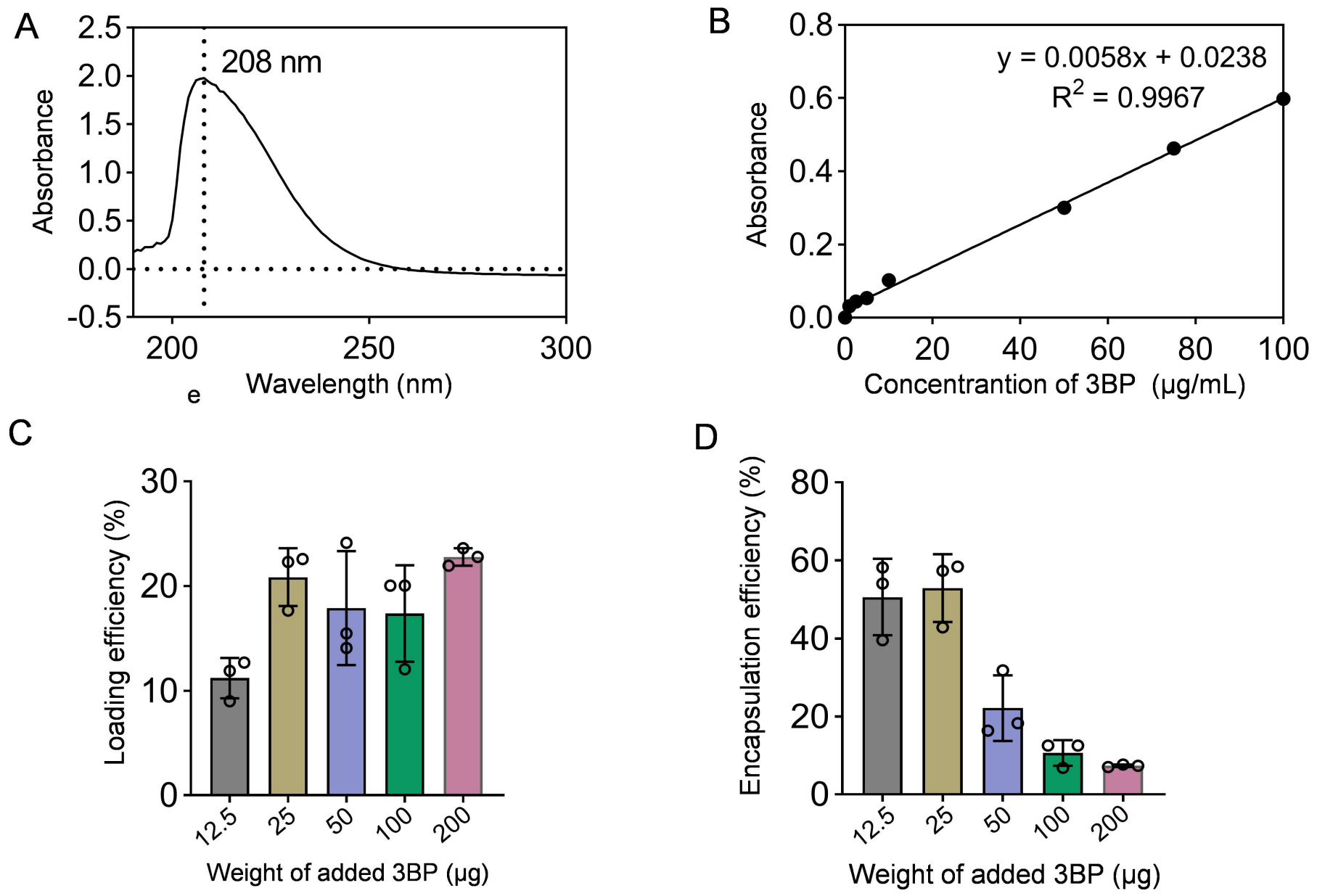

Fig S28. (A,B) UV–Vis absorbance spectra and standard curve of 3BP; (C,D) Loading and encapsulation efficiency of 3BP in FOs. Data are mean ± SD (n = 3).

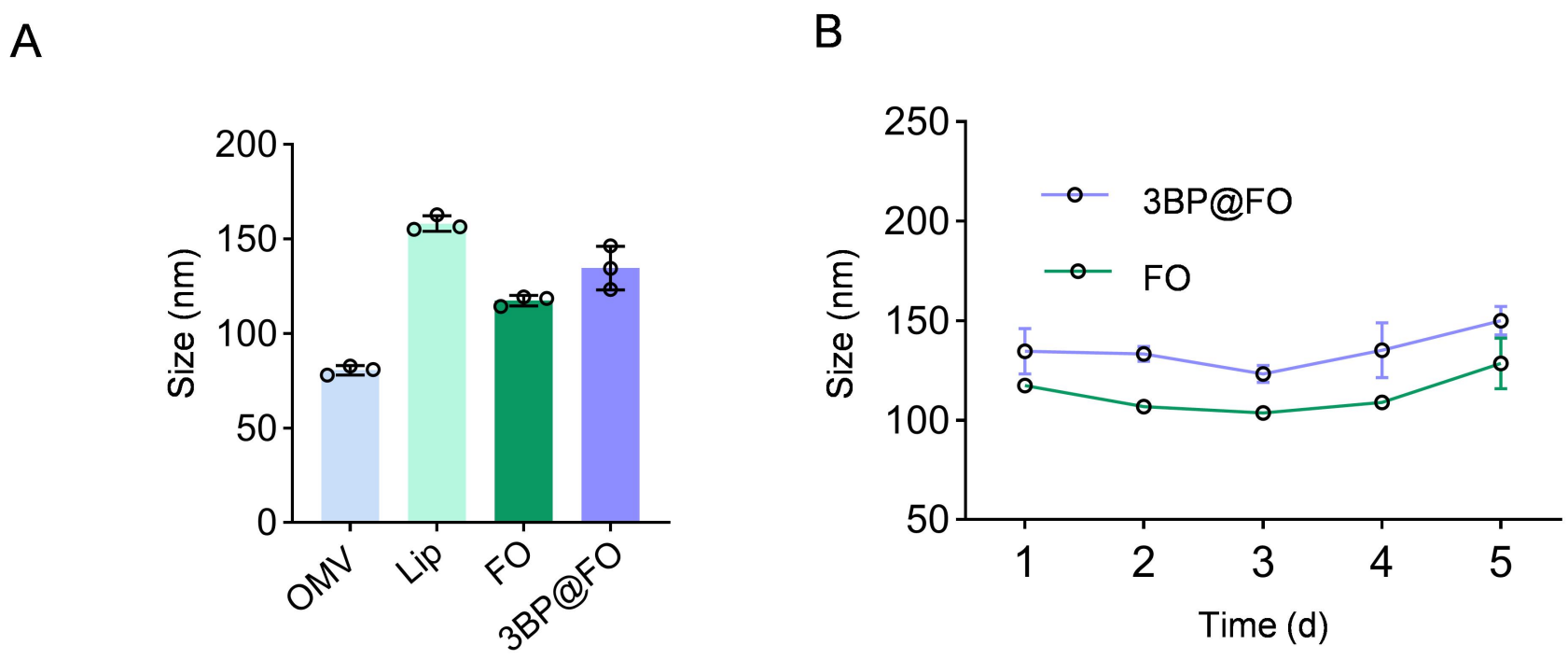

Fig S29. Size distribution(A) and stability(B) of different nanovesicles. Data are mean ± SD (n = 3).

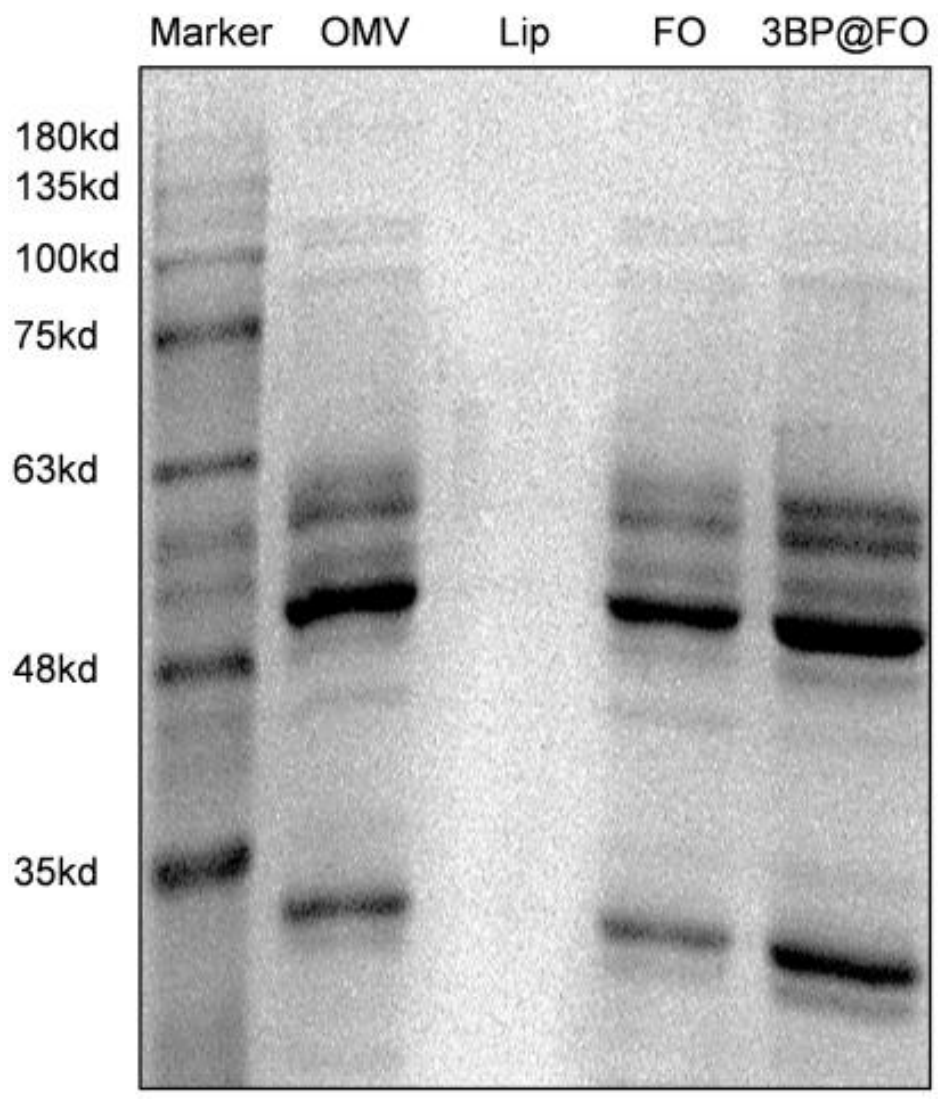

Fig S30. Protein bands of different nanovesicles analyzed by SDS-PAGE.

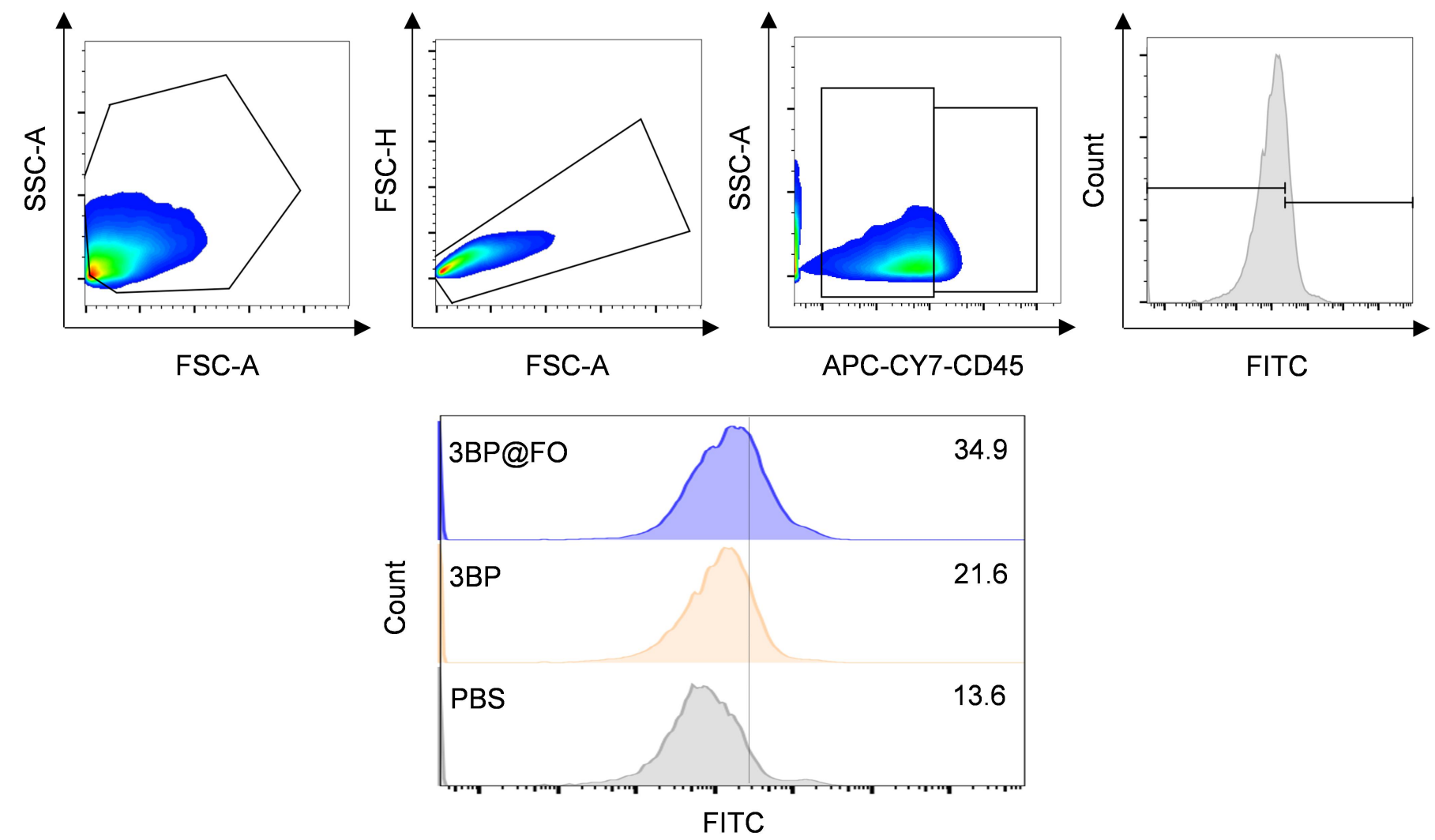

Fig S31. Gating strategy and representative histograms for flow cytometry analysis of FITC–pyruvate^{+} cells in tumor cells (gated on CD45^{-} cells). Data are mean ± SD (n=3).

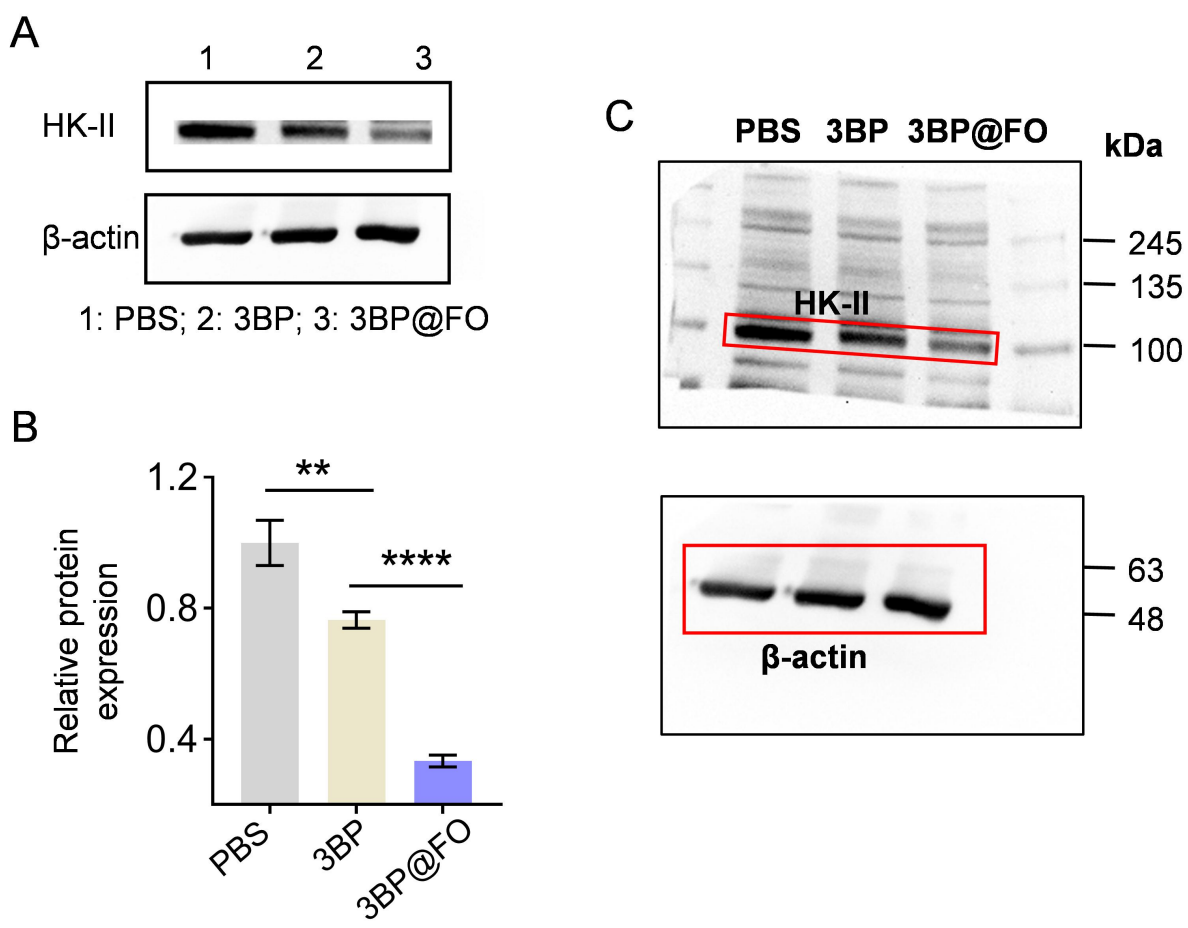

Fig S32. (A)Western blot analysis of HK-II in B16 cells following the indicated treatments. (B) Relative densitometric quantification of HK-II. Protein levels were normalized to β-actin and expressed relative to the control group. Data are presented as mean ± SD (n = 3). Statistical significance was determined using One-way ANOVA. ****P<0.0001, **P<0.01. (C) Representative Western blot images of HK-II and β-actin in B16 cells.

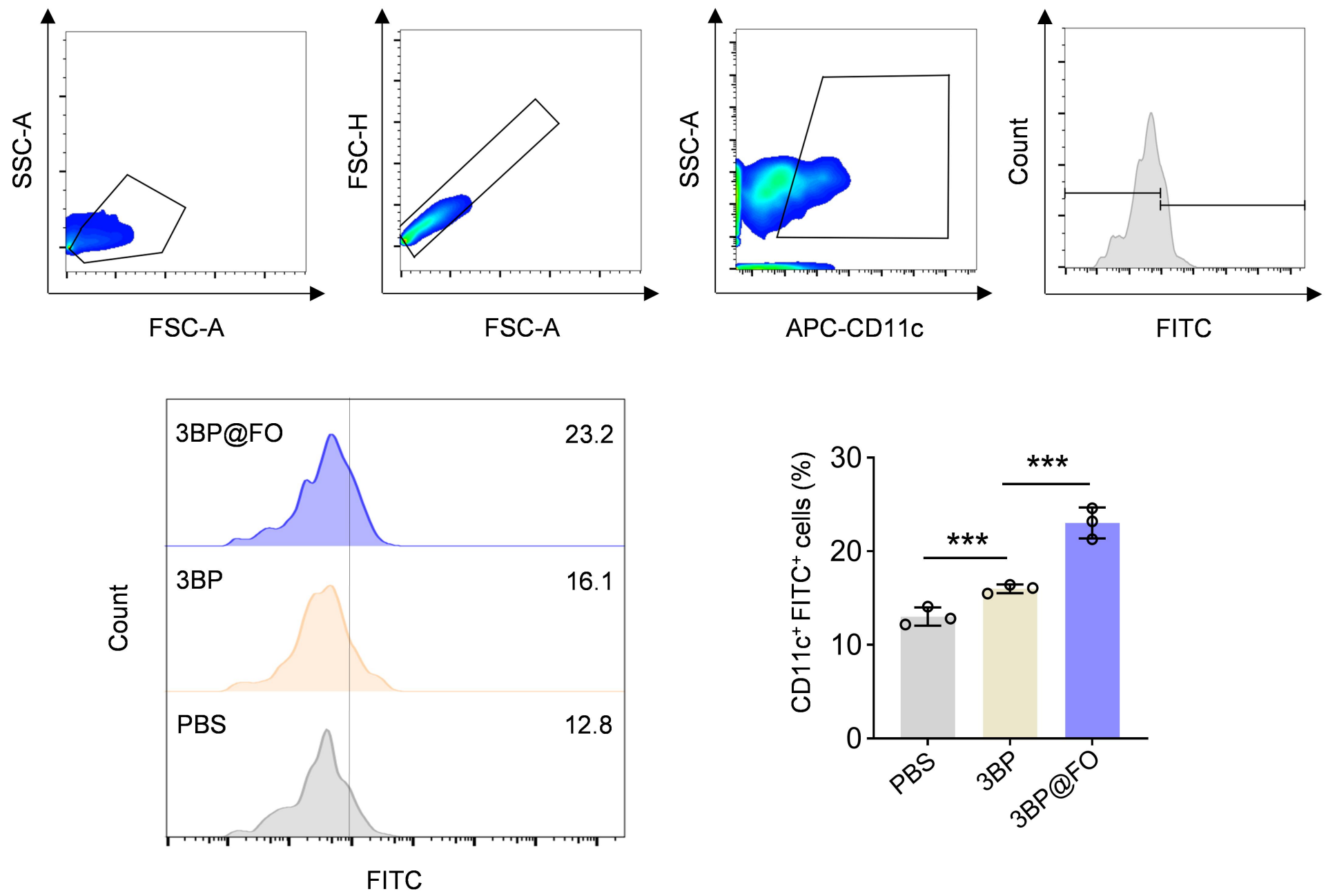

Fig S33. Gating strategy, representative plots, and quantification for flow cytometry analysis of FITC–pyruvate⁺ cells in DCs. Data are mean ± SD (n=3). Statistical significance was determined using One-way ANOVA. ***P<0.001.

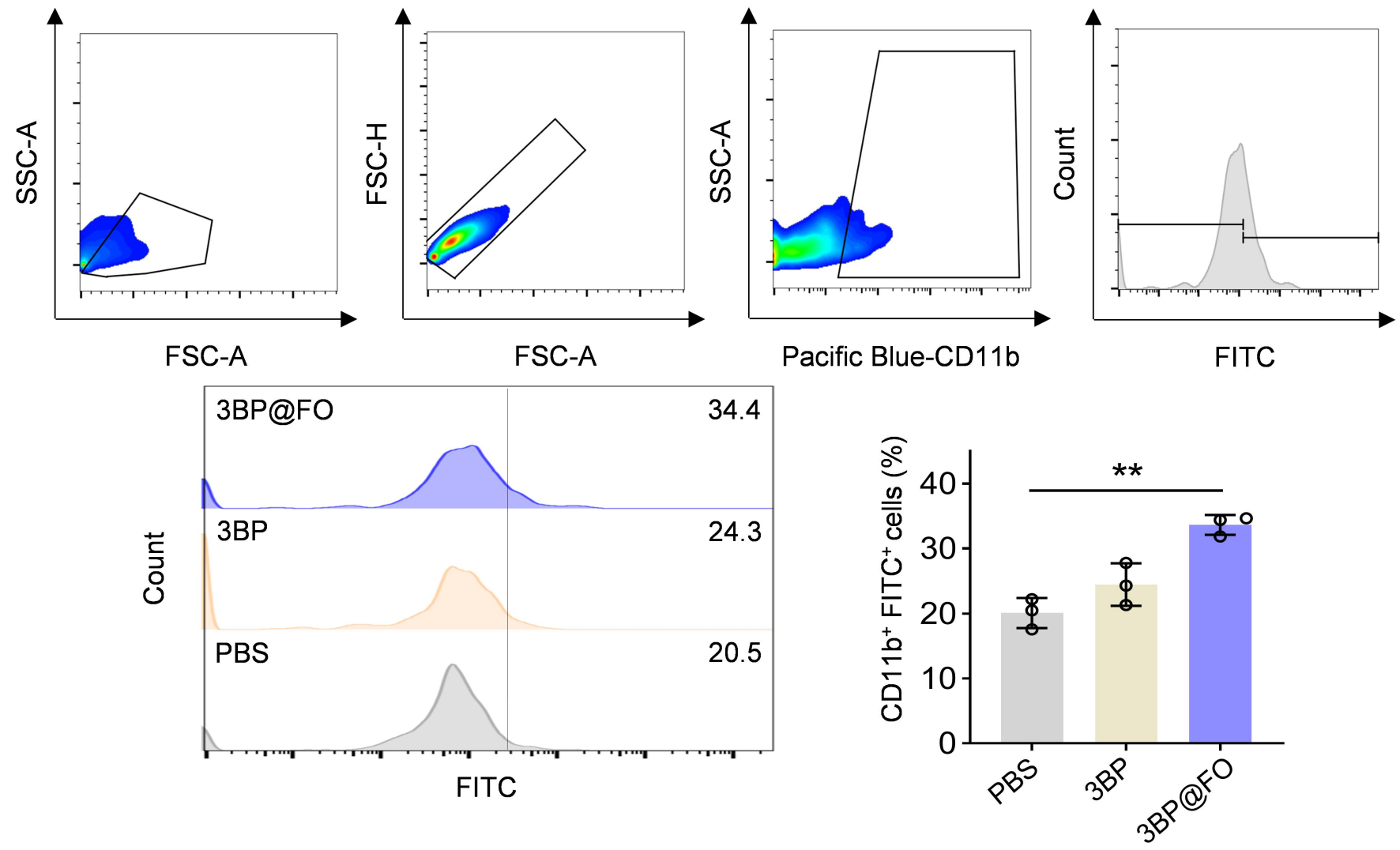

Fig S34. Gating strategy, representative plots, and quantification for flow cytometry analysis of FITC–pyruvate⁺ cells in macrophages. Data are mean ± SD (n=3). Statistical significance was determined using Student's t-test. **P<0.01.

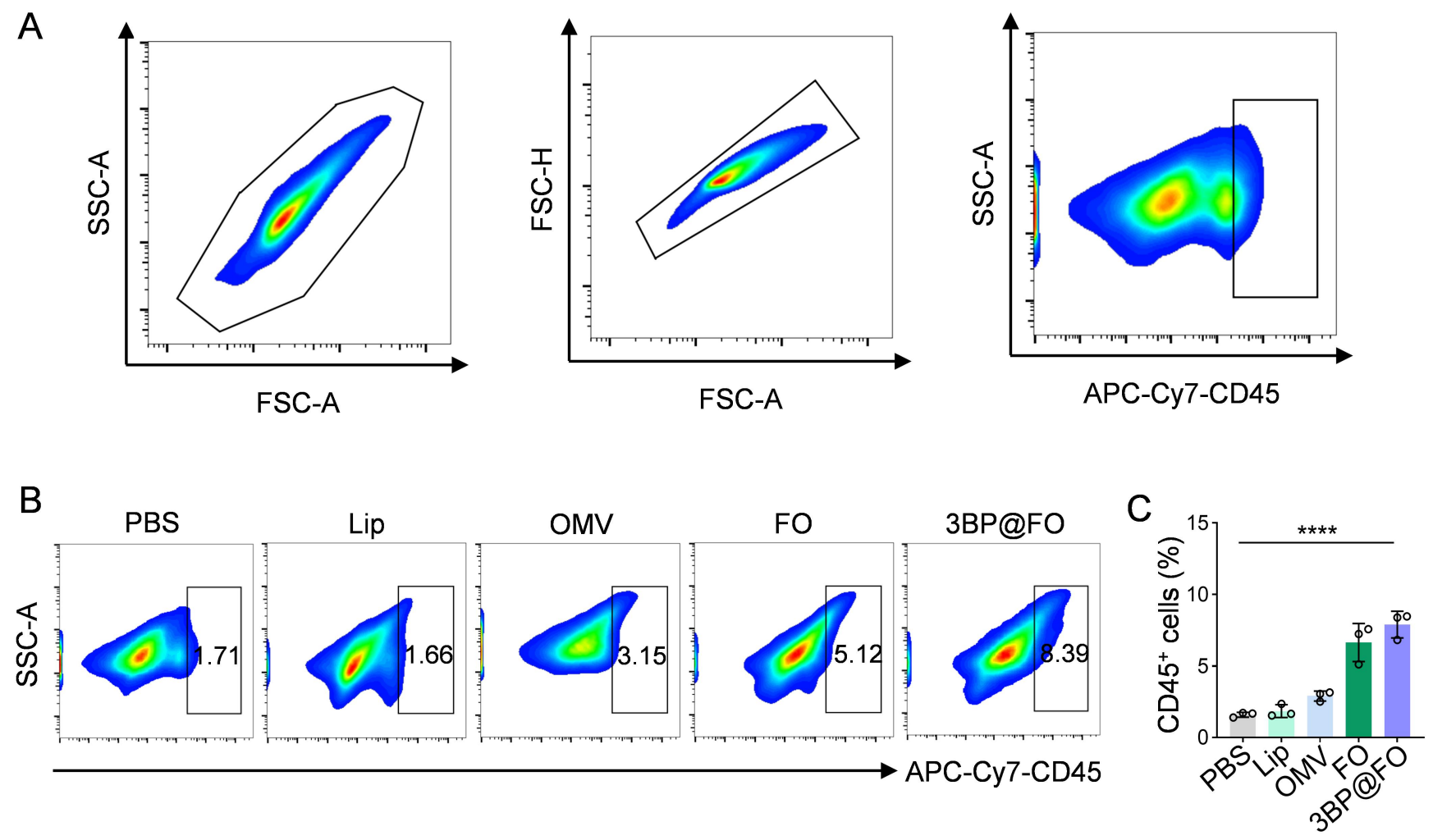

Fig S35. (A-C) Gating strategy, representative plots, and quantification for flow cytometry analysis of $CD45^+$ cells in the TME. Data are mean ± SD (n=3). Statistical significance was determined using Student's t-test. ****P<0.0001.

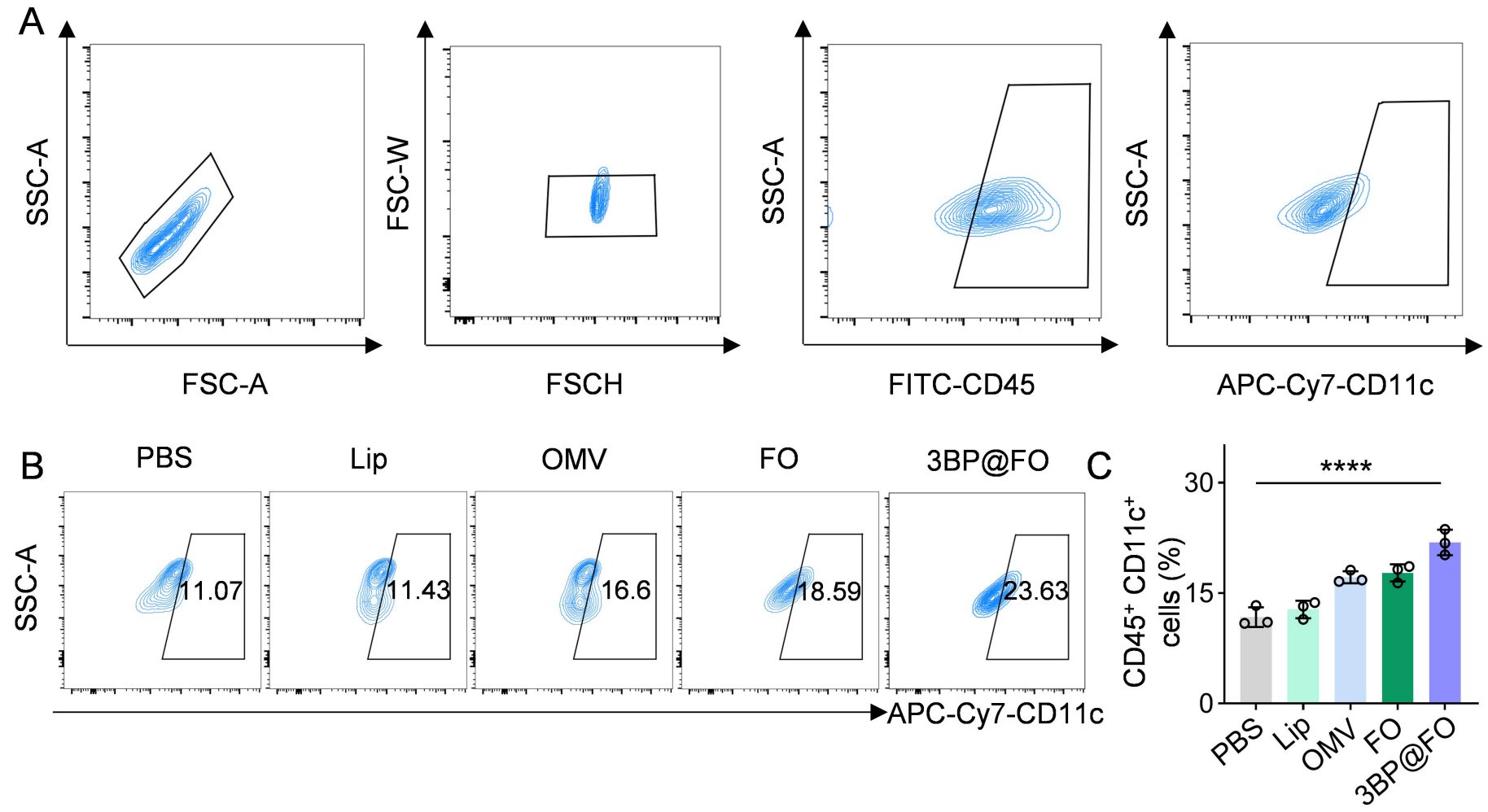

Fig S36. (A-C) Gating strategy, representative plots, and quantification for flow cytometry analysis of DCs ($CD45^{+}CD11c^{+}$) in the TME. Data are mean ± SD (n=3). Statistical significance was determined using Student's t-test. ****P<0.0001.

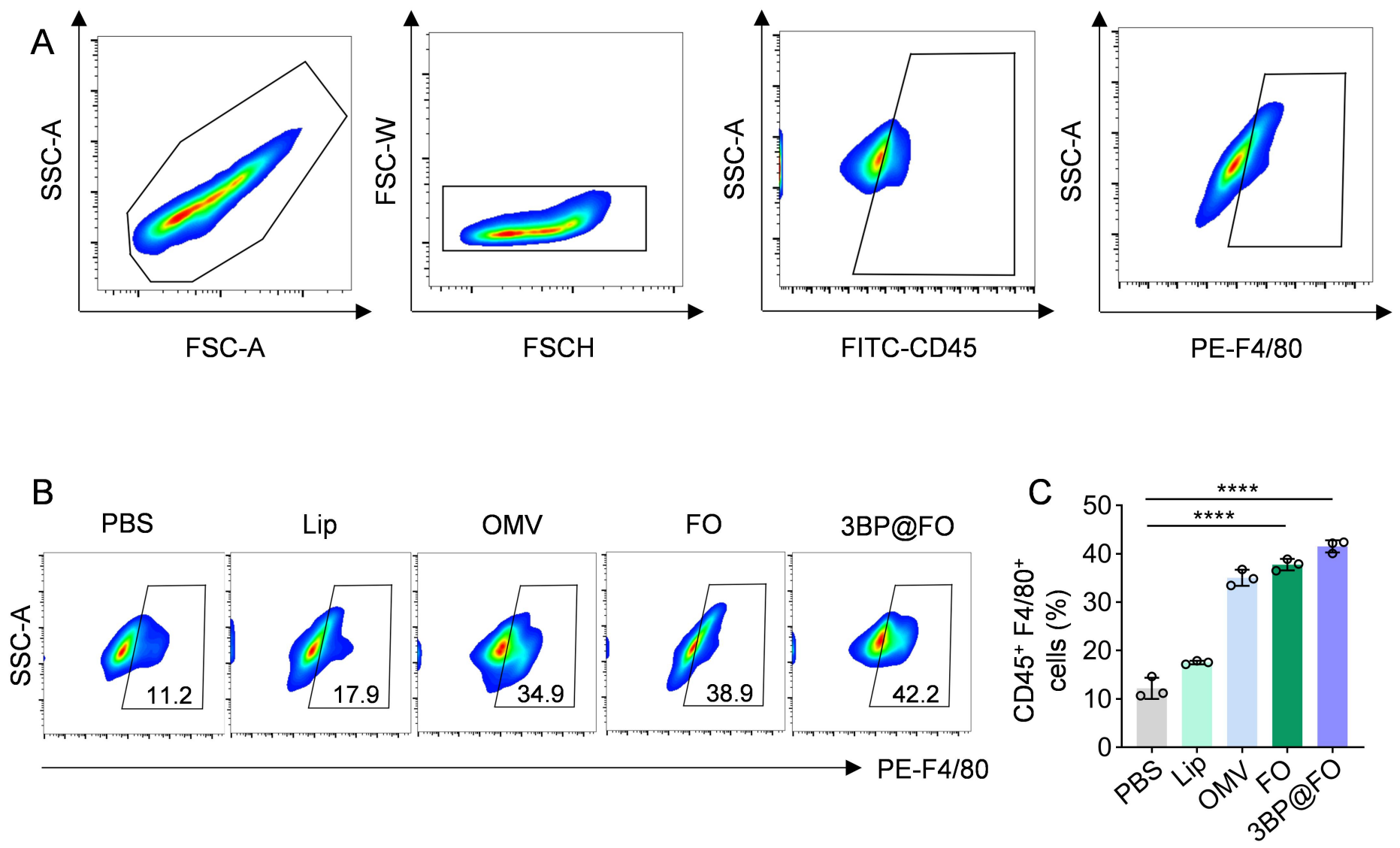

Fig S37. (A-C) Gating strategy, representative plots, and quantification for flow cytometry analysis of macrophages ($CD45^{+}F4/80^{+}$) in the TME. Data are mean ± SD (n=3). Statistical significance was determined using One-way ANOVA. ****P<0.0001.

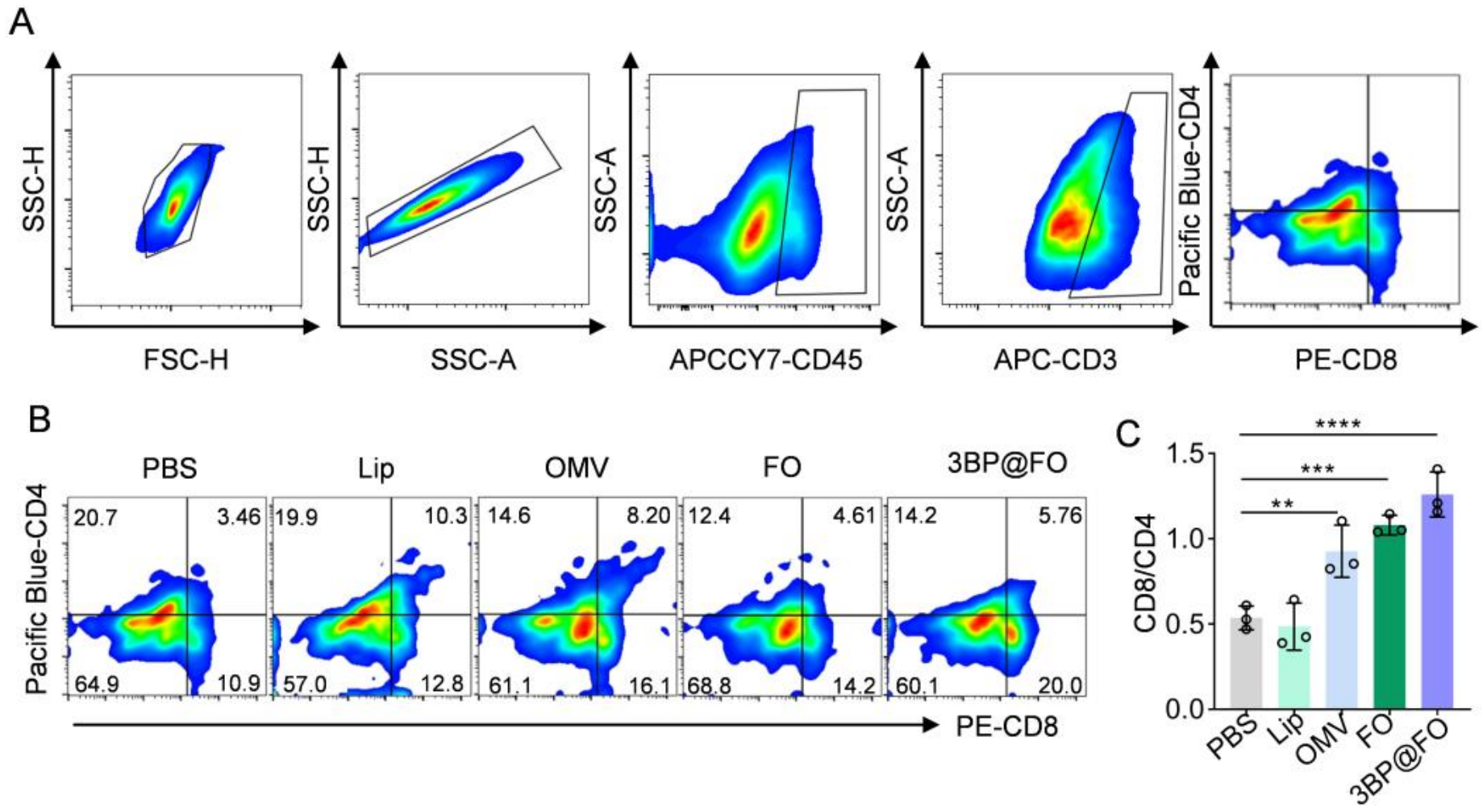

Fig S38. (A-C) Gating strategy, representative plots, and quantification for flow cytometry analysis of $CD3^{+}CD8^{+}$ and $CD3^{+}CD4^{+}$ cells in the TME. Data are mean ± SD (n=3). Statistical significance was determined using One-way ANOVA. ****P<0.0001, ***P<0.001, **P<0.01.

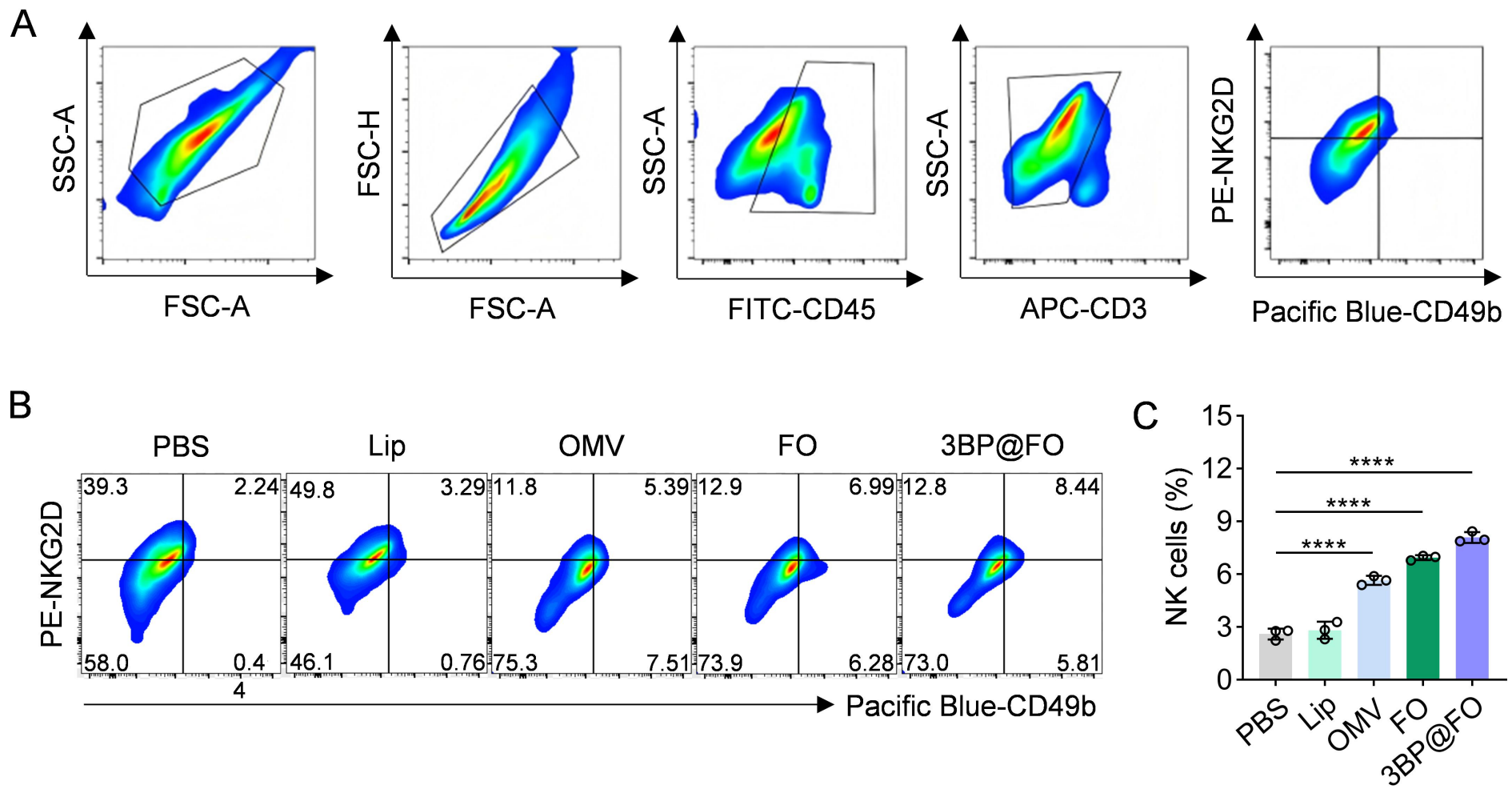

Fig S39. (A-C) Gating strategy, representative plots, and quantification for flow cytometry analysis of NK cells ($CD45^+CD49b^+NKG2D^+$) in the TME. Data are mean ± SD (n=3). Statistical significance was determined using One-way ANOVA. ****P<0.0001.

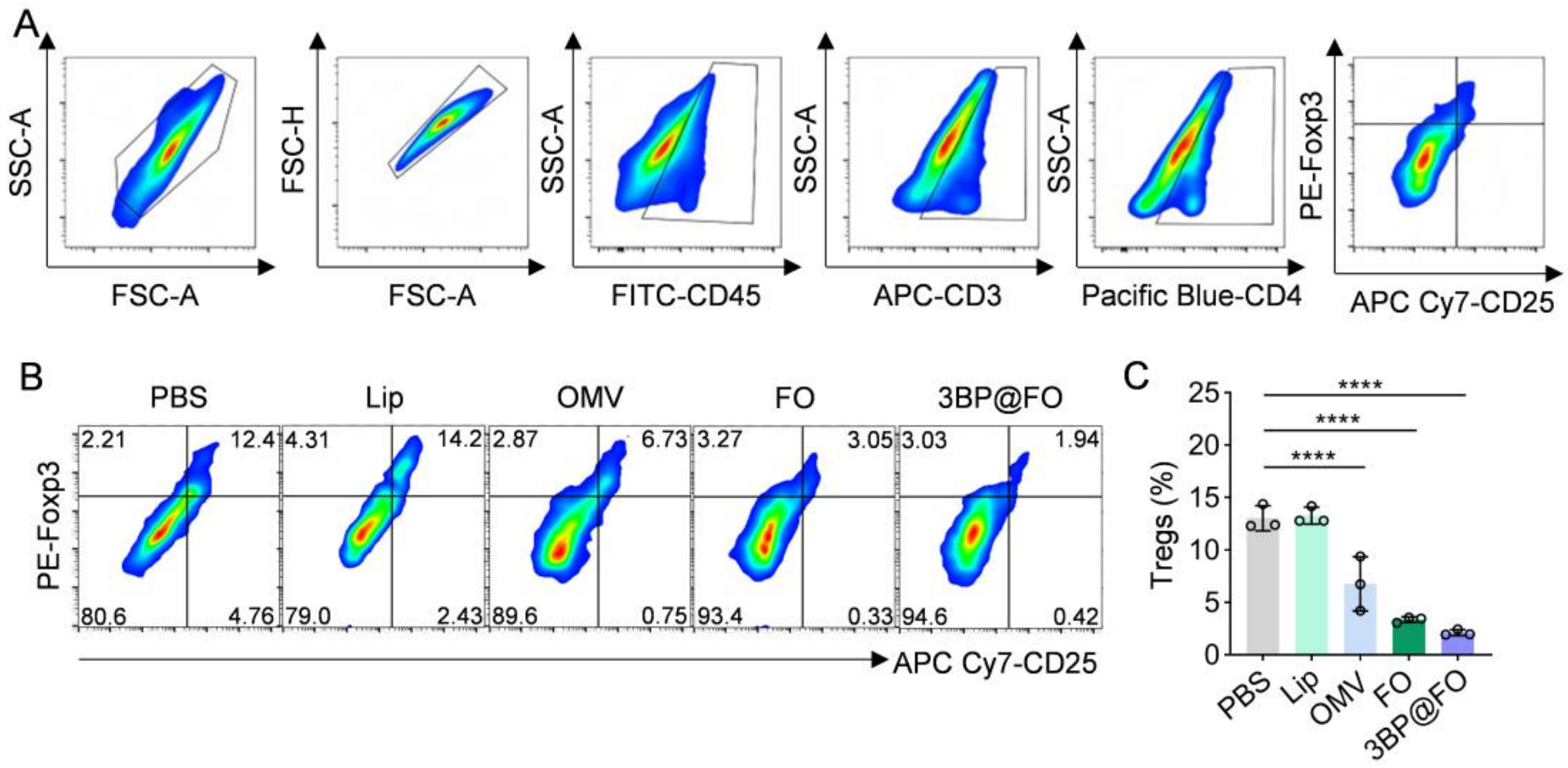

Fig S40. (A-C) Gating strategy, representative plots, and quantification for flow cytometry analysis of Treg cells ($CD45^+CD3^+CD4^+CD25^+Foxp3^+$) in the TME. Data are mean ± SD (n=3). Statistical significance was determined using One-way ANOVA. ****P<0.0001.

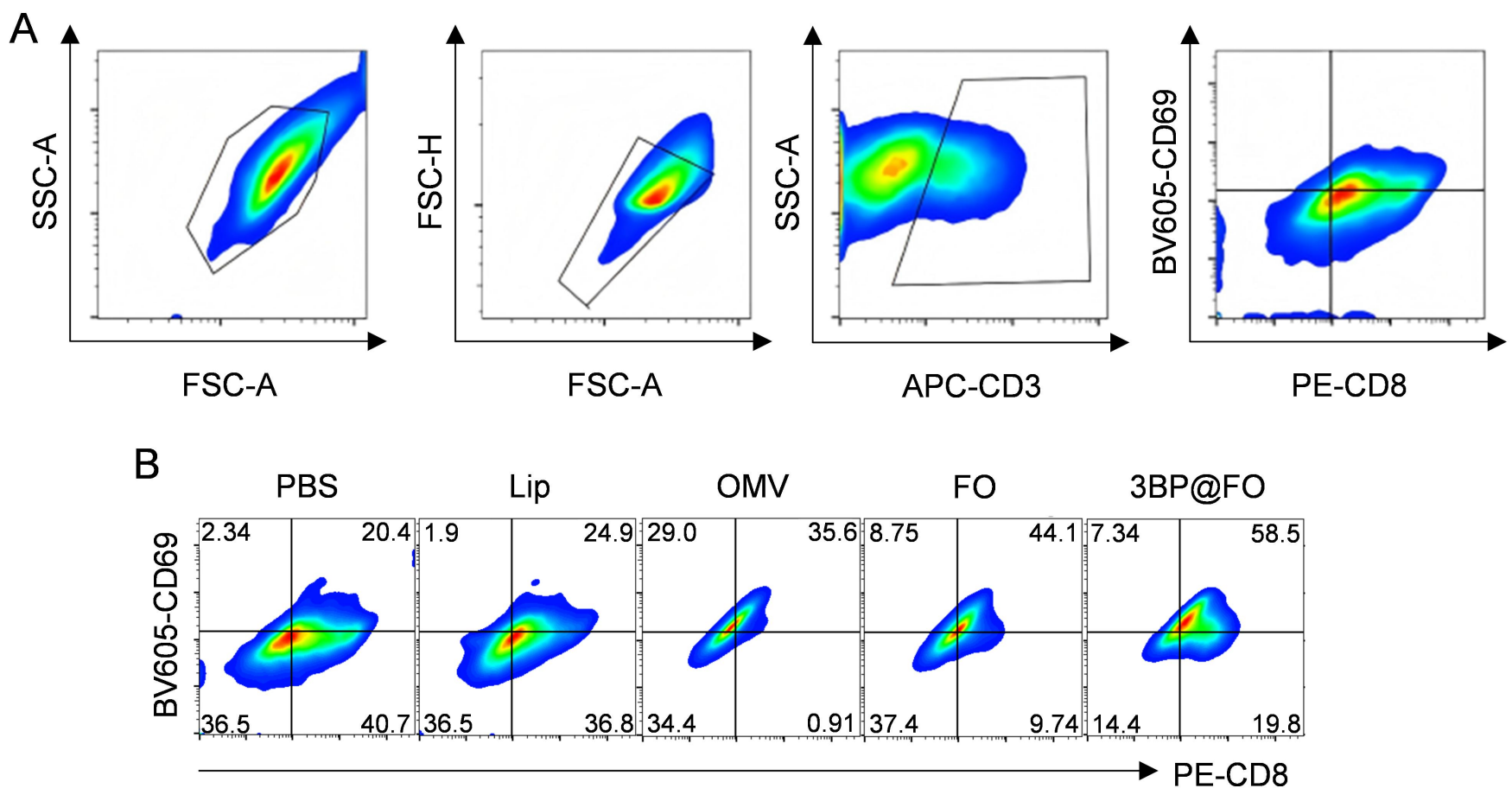

Fig S41. (A-B)Gating strategy, and Representative plots for flow cytometry analysis of $CD3^{+}CD8^{+}CD69^{+}$ cells in the TME.

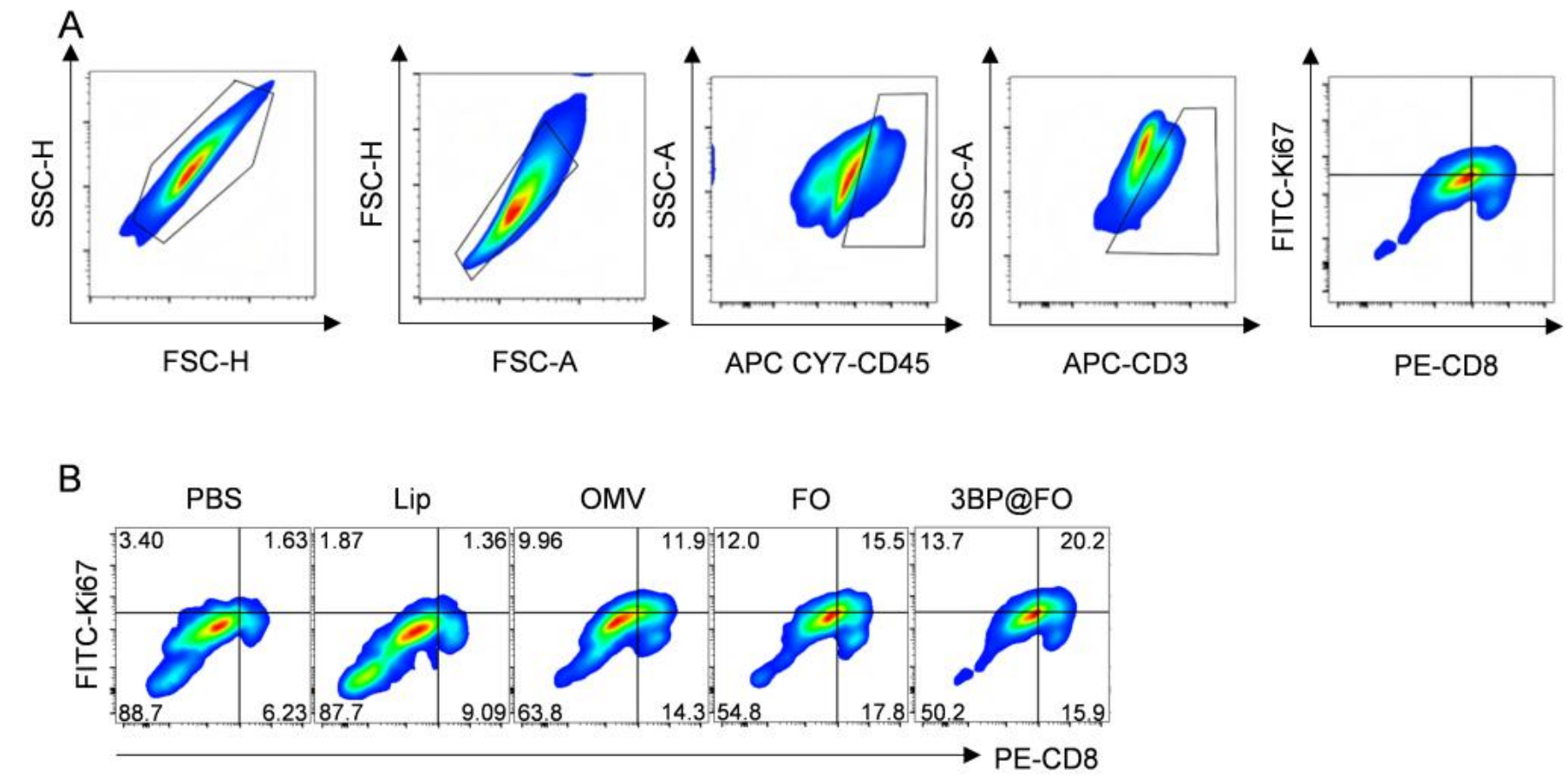

Fig S42. (A,B) Gating strategy and representative plots for flow cytometry analysis of CD3$^+$CD8$^+$Ki67$^+$ cells in the TME.

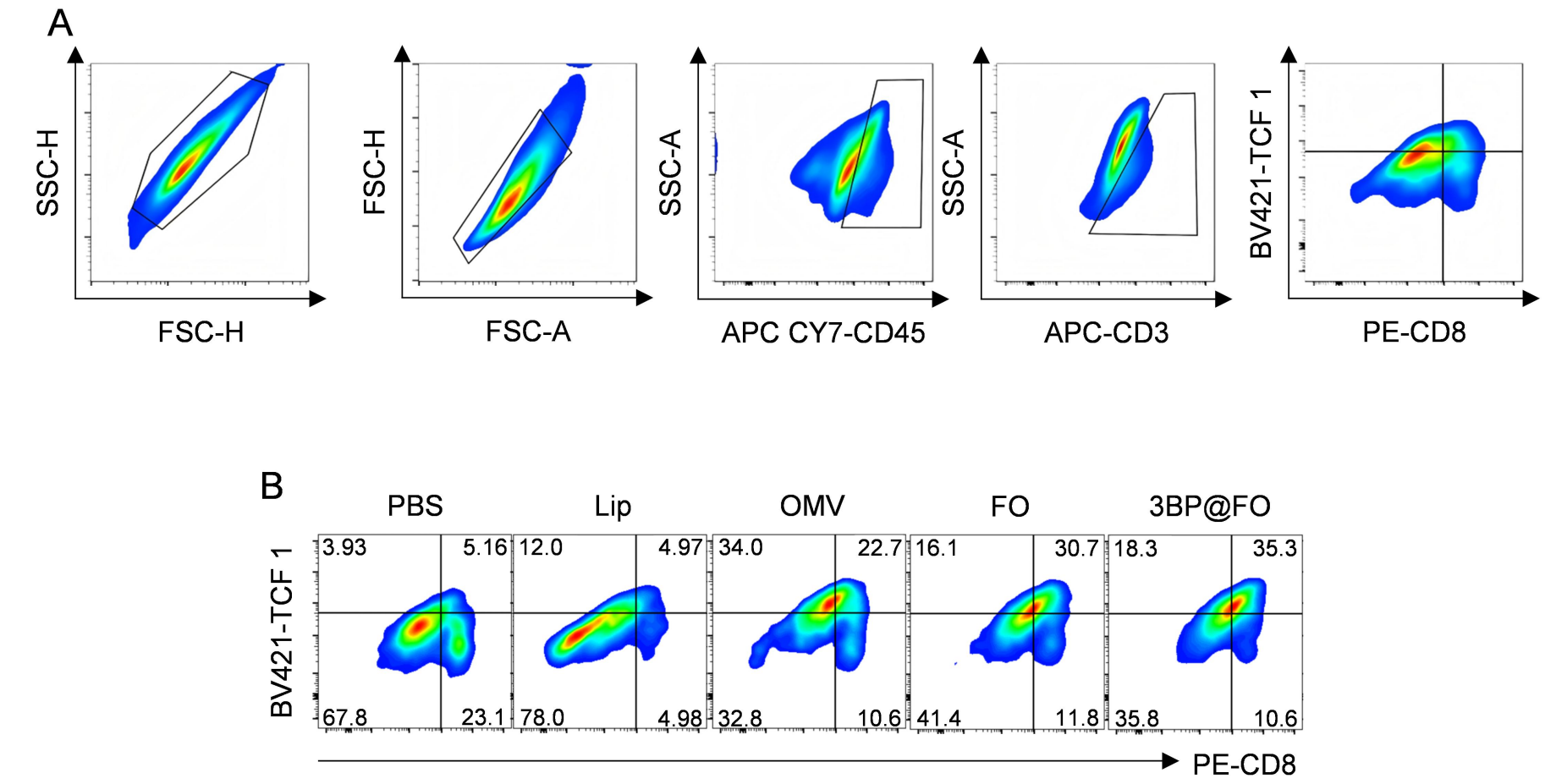

Fig S43. (A,B) Gating strategy and representative plots for flow cytometry analysis of $CD3^{+}CD8^{+}TCF1^{+}$ cells in the TME.

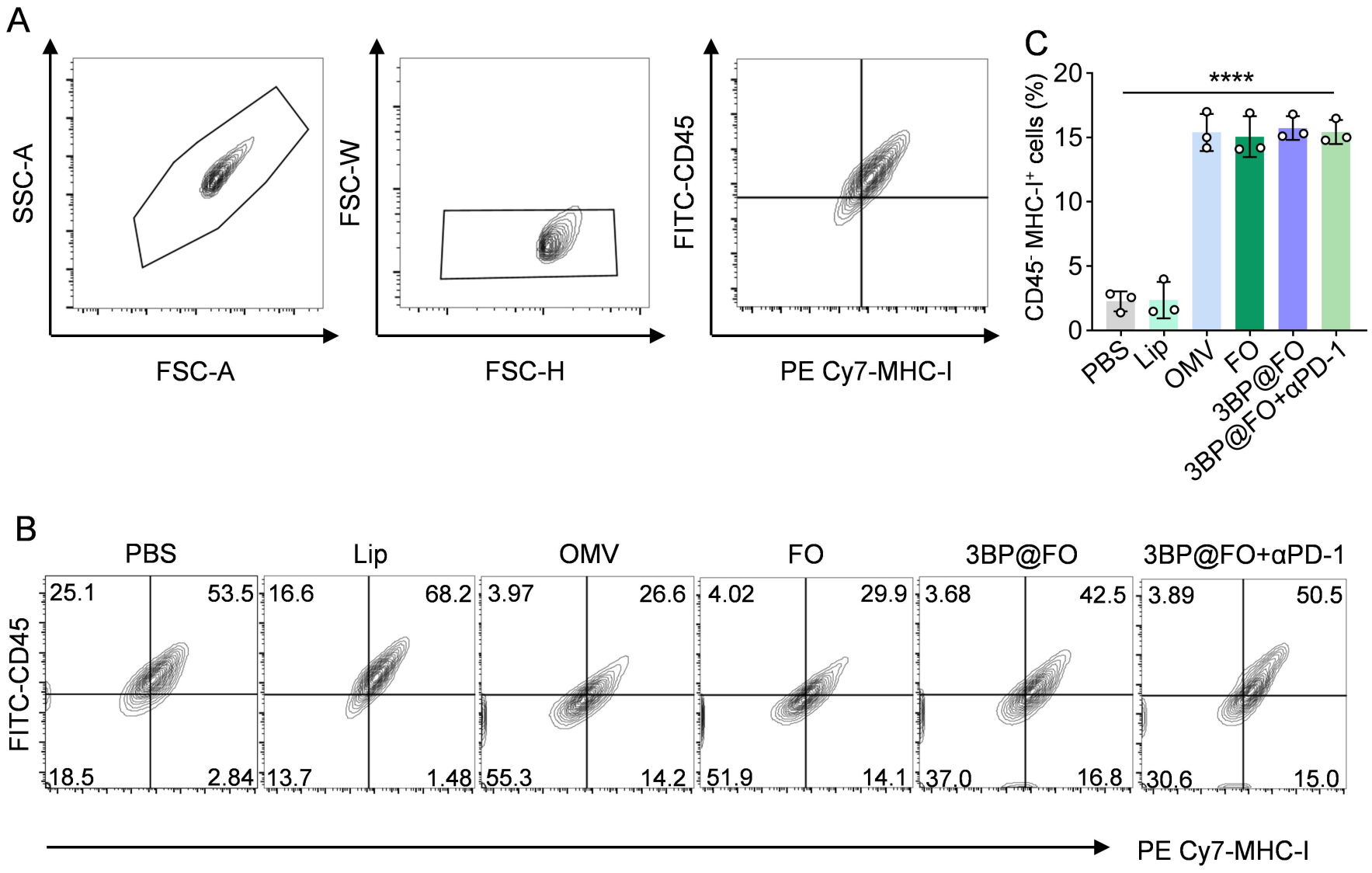

Fig S44. (A-C) Gating strategy, representative plots, and quantification for flow cytometry analysis of MHC-I$^+$ tumor cells (CD45$^-$) in the TME. Data are mean ± SD (n=3). Statistical significance was determined using Student's t-test. ****P<0.0001.

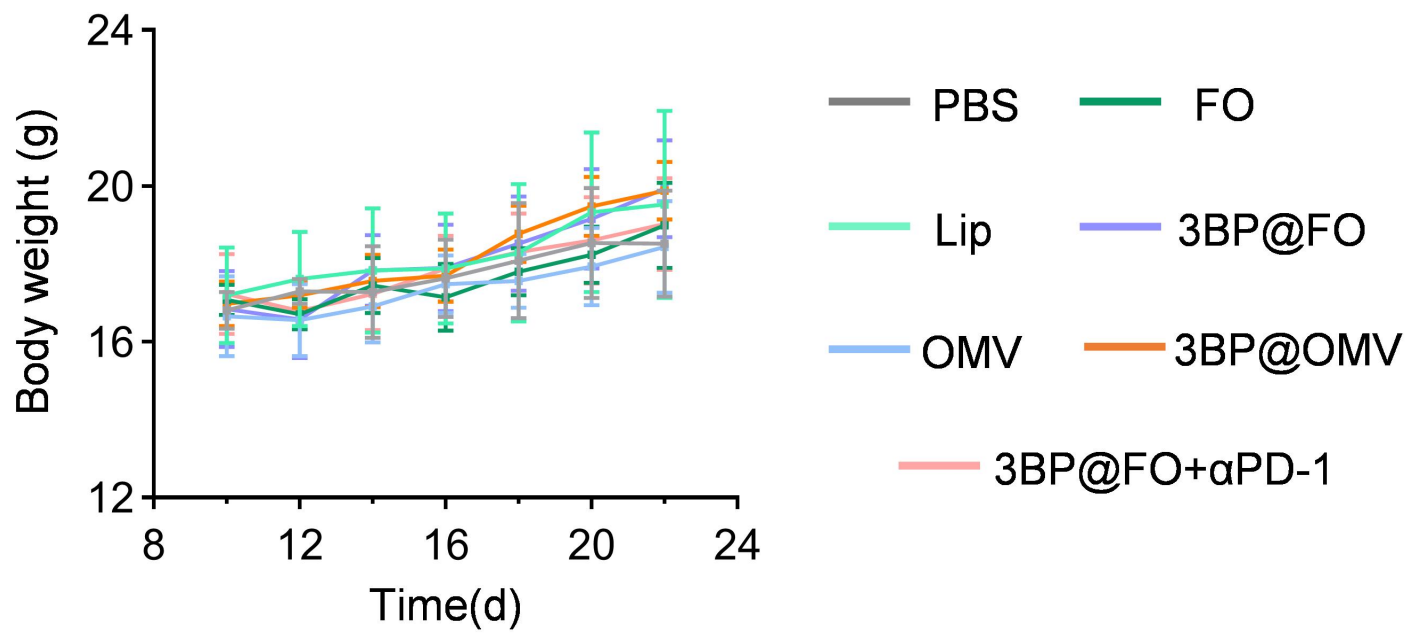

Fig S45. Body weight changes of B16-bearing mice during the treatment. Data are mean ± SD (n=8).

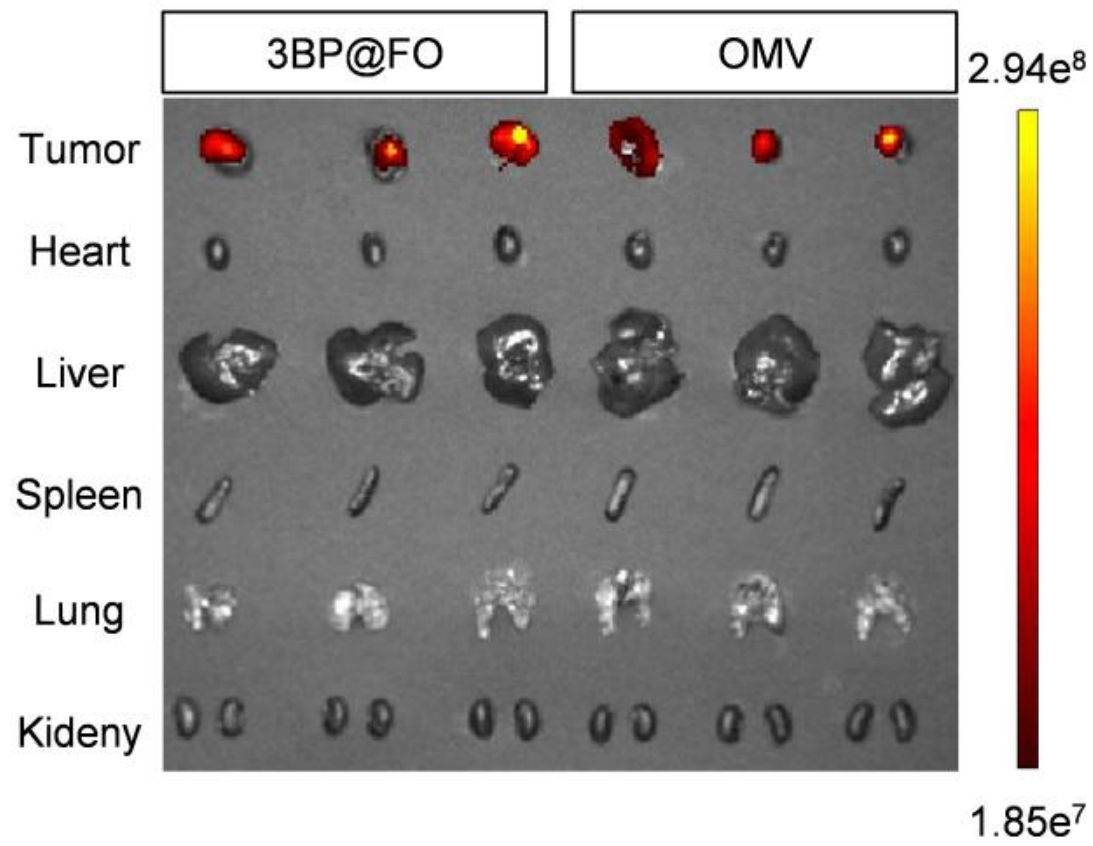

Fig S46. *Ex vivo* fluorescence imaging of major organs and tumors at 72 h post intratumoral administration

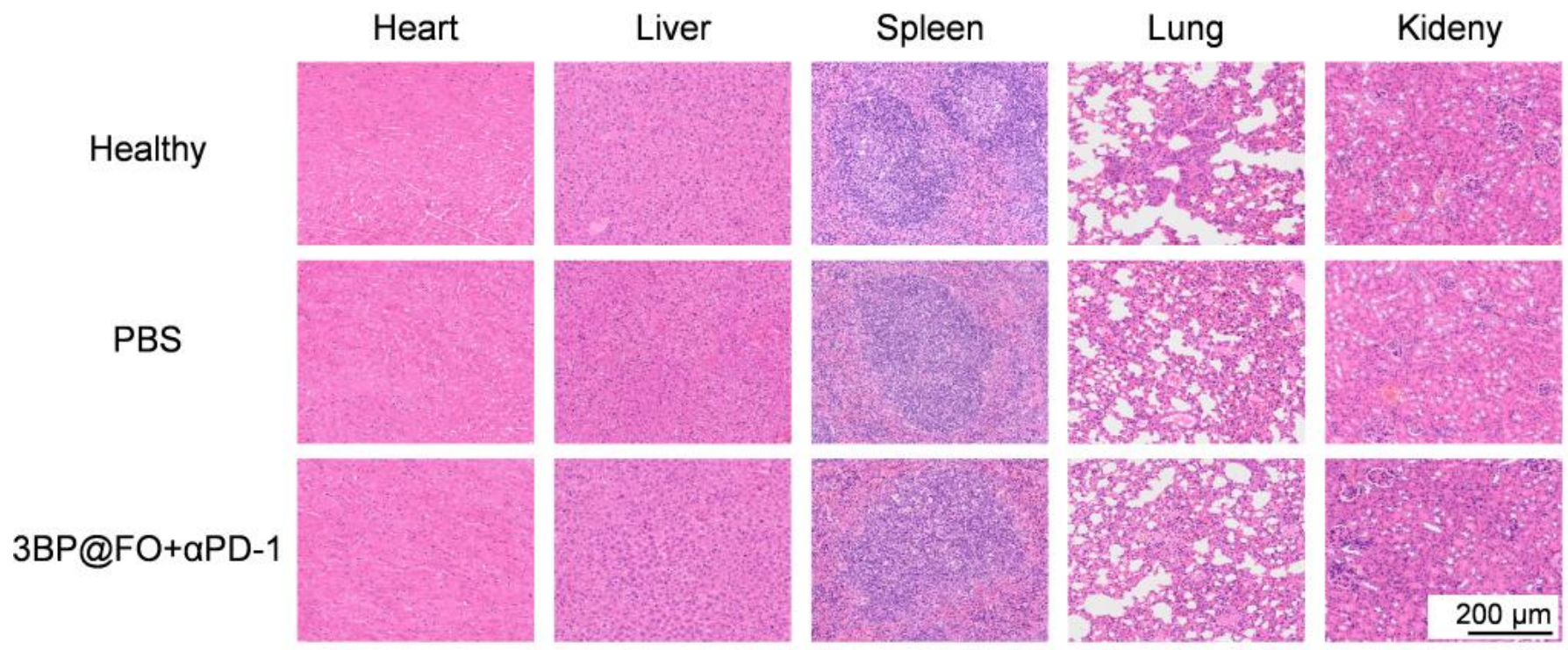

Fig S47. H&E staining of major organs at the endpoint of the antitumor experiment against B16 tumors.

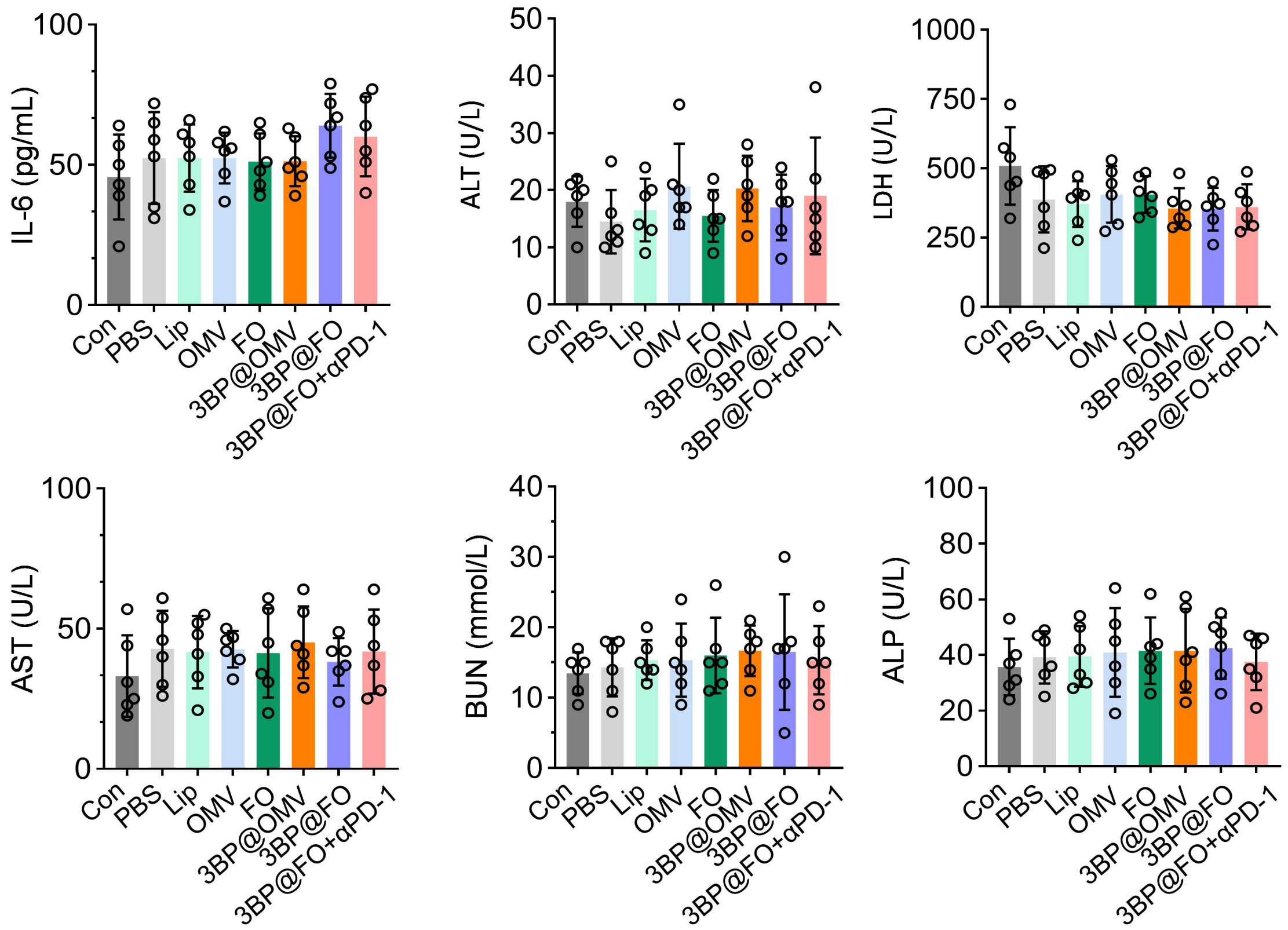

Fig S48. Serum IL-6 levels and biochemical indices of alkaline phosphatase (ALP), aspartate transaminase (AST), alanine transaminase (ALT), lactate dehydrogenase (LDH), and urea nitrogen (BUN) in different groups at the endpoint.

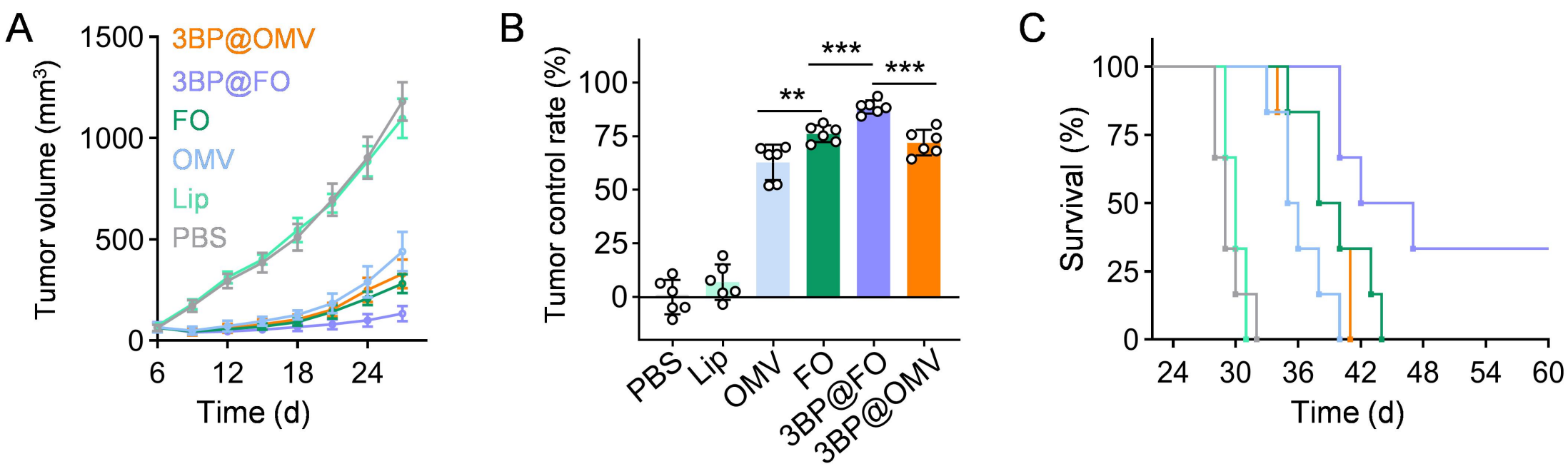

Fig S49. Tumor growth curves (A), tumor inhibition rates (B), and survival percentages (C) of CT26-bearing mice. Data are mean ± SD (n=6). Statistical significance was determined using One-way ANOVA. ***P<0.001, **P<0.01.

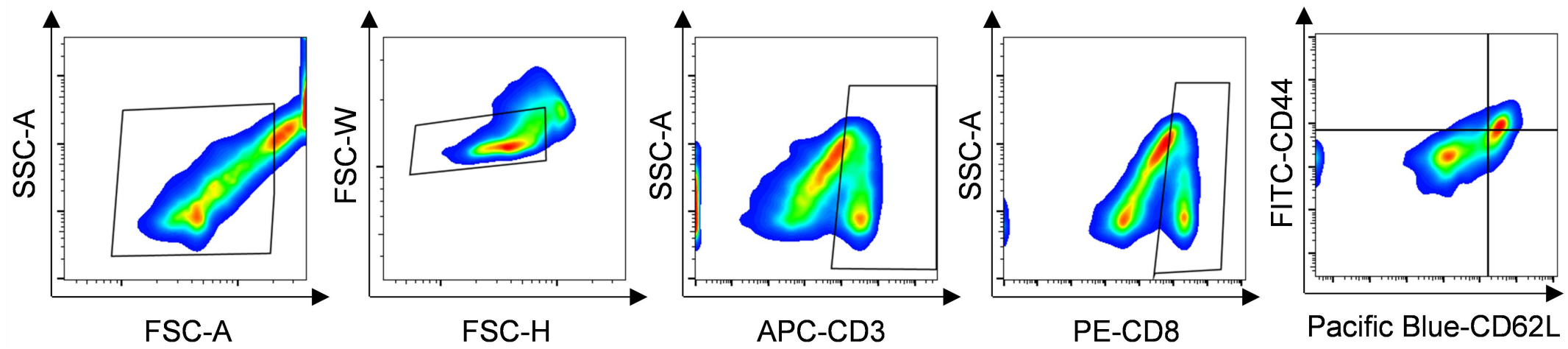

Fig S50. Gating strategy for flow cytometry analysis of effector memory T cells in lymph nodes.

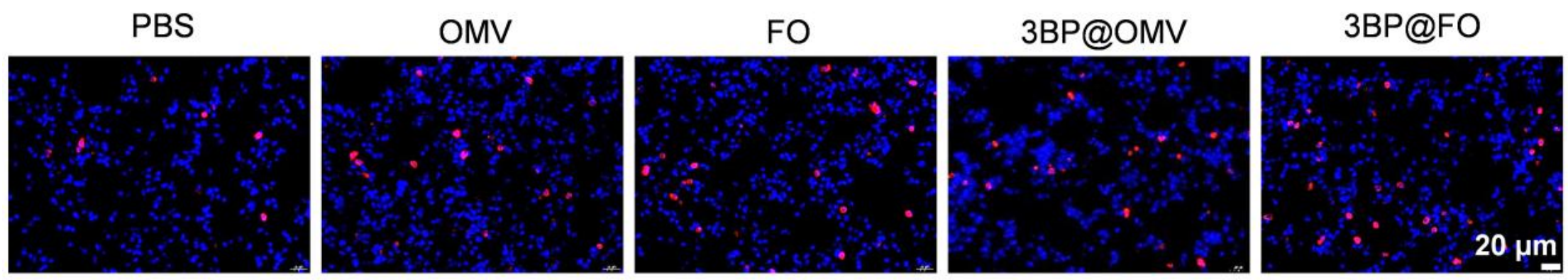

Fig S51 . Distribution of $CD8^{+}$ T cells in the lungs of different treatment groups in the lung metastasis model.

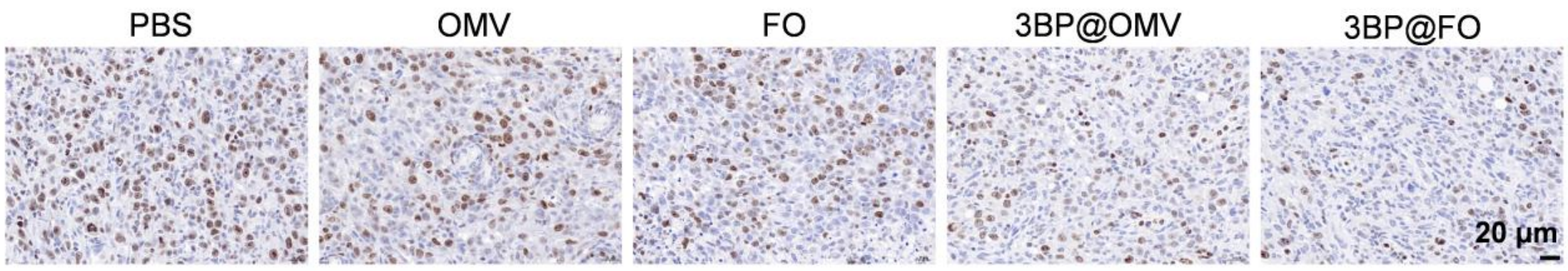

Fig S52. Representative Ki67 staining images of tumor tissues collected at the experimental endpoint.

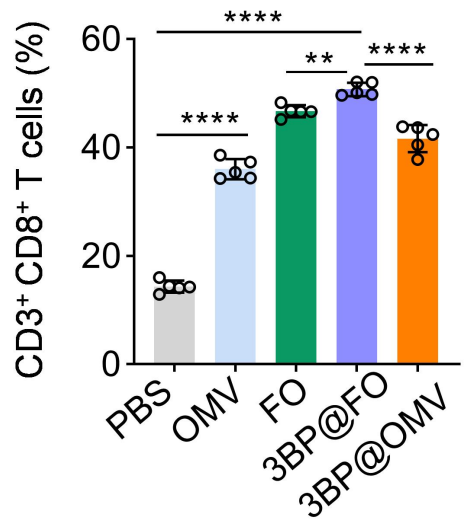

Fig S53. Quantification of $CD3^{+}CD8^{+}$ T cells in the spleens of mice receiving the indicated treatments. Data are mean ± SD (n = 5). Statistical significance was determined using One-way ANOVA. ****P < 0.0001, **P < 0.01.

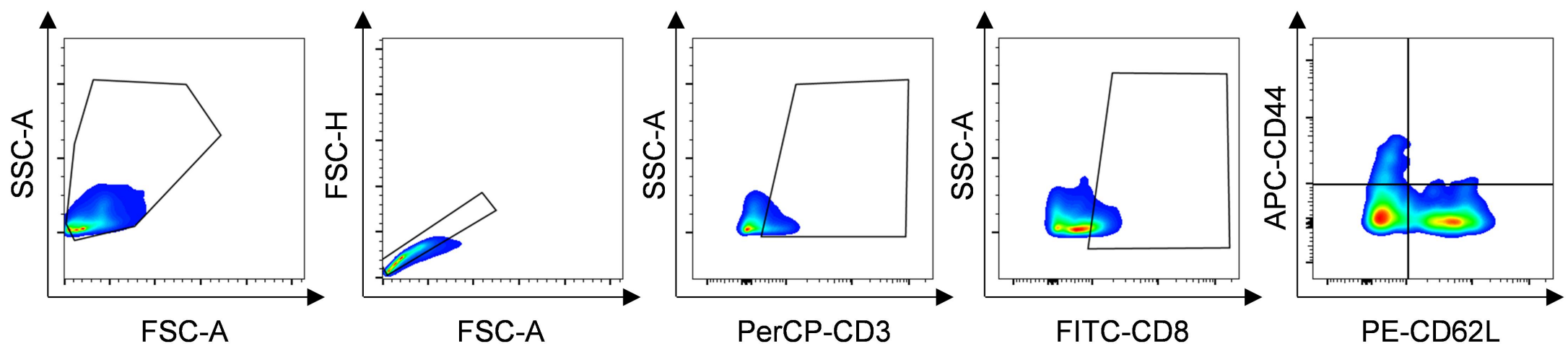

Fig S54. Gating strategy for flow cytometry analysis of long-term effector memory T cells in the spleens of mice receiving the indicated treatments.

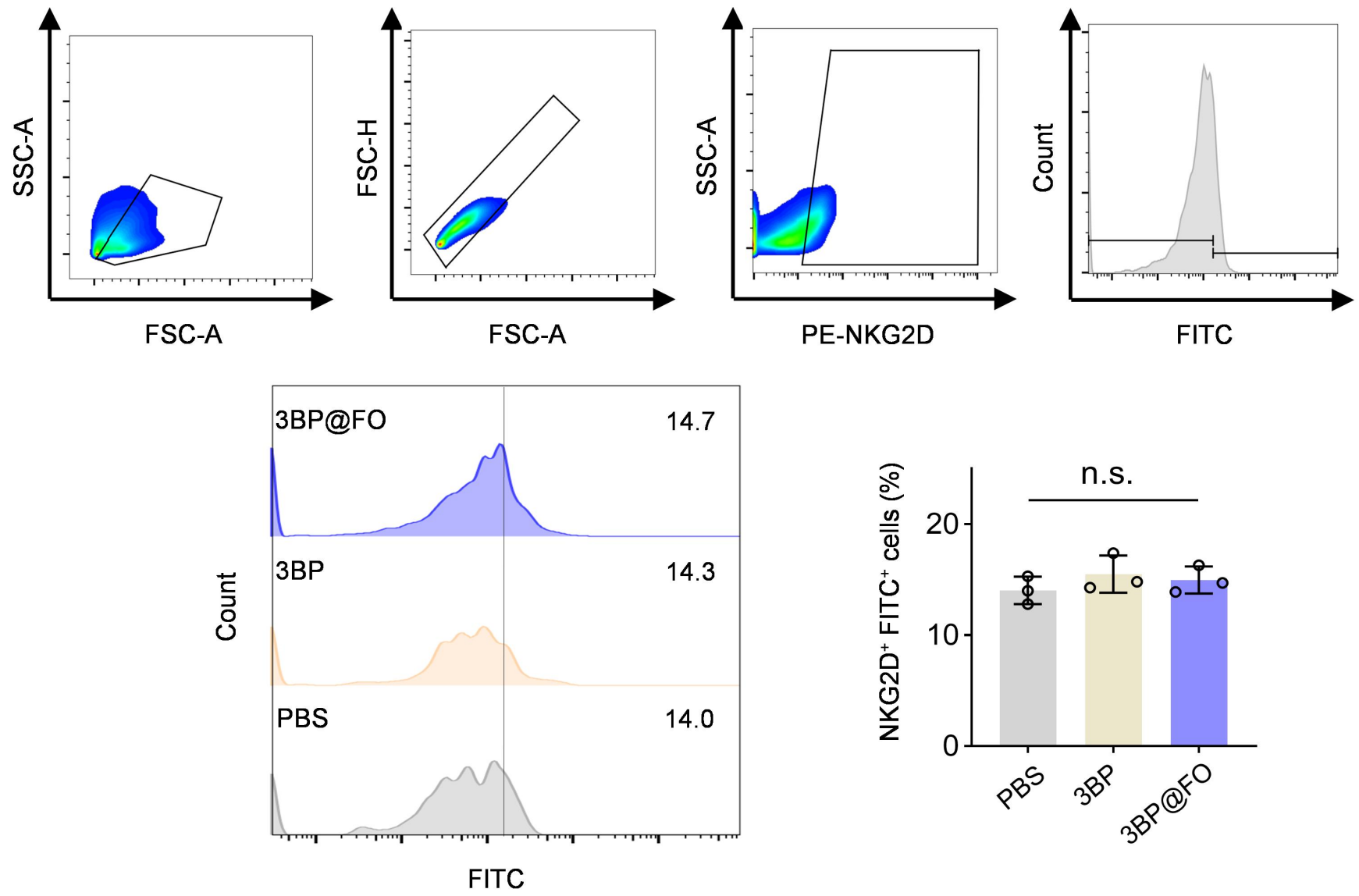

Fig S55. Gating strategy, representative plots, and quantification for flow cytometry analysis of FITC–pyruvate⁺ cells in NK cells. Data are mean ± SD (n=3). Statistical significance was determined using Student's t-test. n.s., not significant.

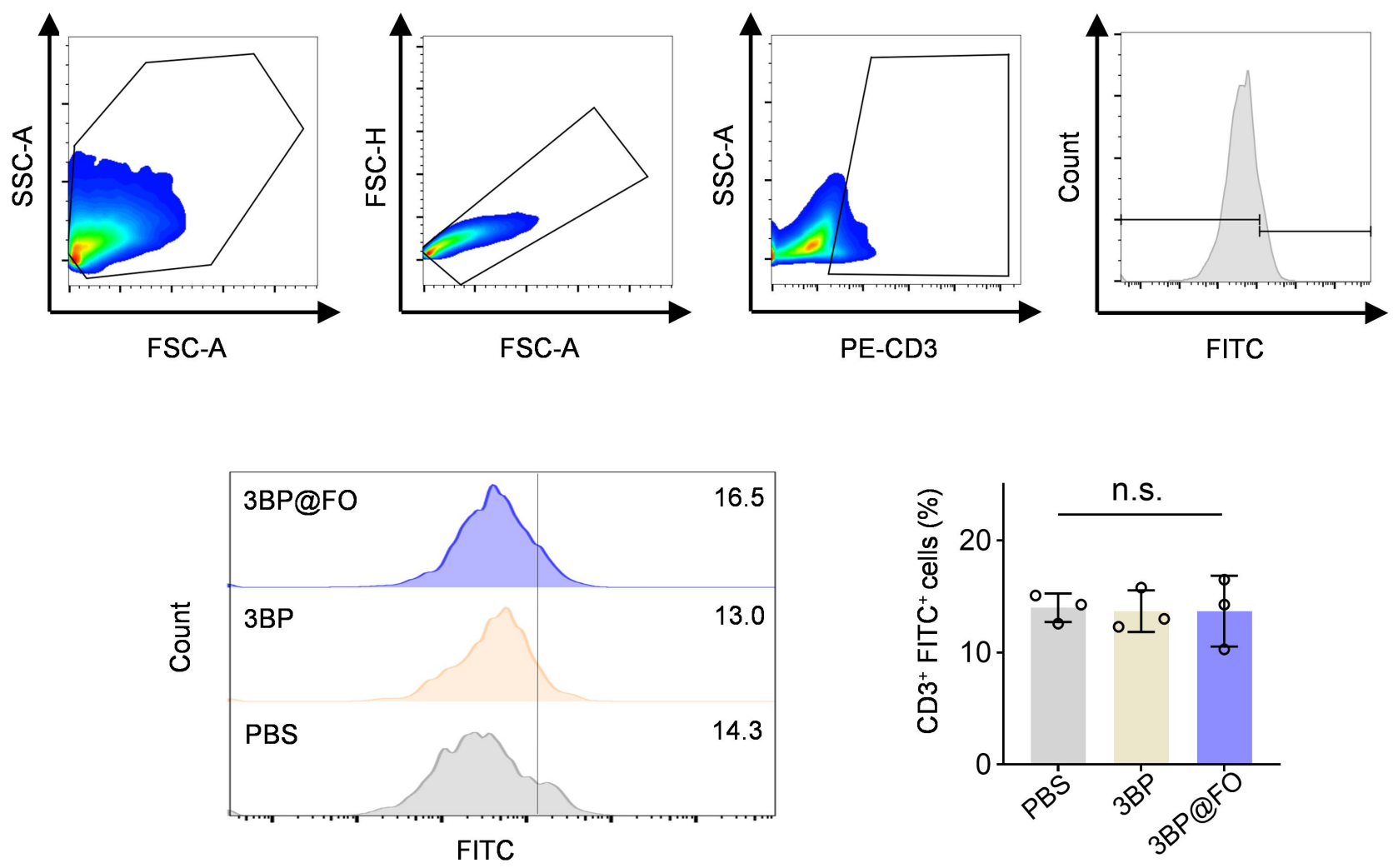

Fig S56. Gating strategy, representative plots, and quantification for flow cytometry analysis of FITC–pyruvate⁺ cells in T cells. Data are mean ± SD (n=3). Statistical significance was determined using Student's t-test. n.s., not significant.

Supplementary Table 1 Corresponding primer list.

Gene	Primers	
	Forward (5'-3')	Reverse (5'-3')
Tlr4	AGCTTCTCCAATTTTTCAGAACTTC	TGAGAGGTGGTGTAAGCCATGC
Nlrc5	TCAGCCCAGAACAAGTATCC	TGGGCACAGACTTCCATTAG
Nfkb	AGAGGATTTGCTGAGGGTTG	TGCTGAGGATTCTGTCGTGT
Ccl5	AGATCTCTGCAGCTGCCCTCA	GGAGCACTTGCTGCTGGTGTAG
GAPDH	CATCACTGCCACCCAGAAGACTG	ATGCCAGTGAGCTTCCCGTTCAG
